# Host Iron Binding Proteins Acting as Niche Indicators for *Neisseria meningitidis*

**DOI:** 10.1371/journal.pone.0005198

**Published:** 2009-04-08

**Authors:** Philip W. Jordan, Nigel J. Saunders

**Affiliations:** The Bacterial Pathogenesis and Functional Genomics Group, The Sir William Dunn School of Pathology, University of Oxford, Oxford, United Kingdom; The Research Institute for Children at Children's Hospital New Orleans, United States of America

## Abstract

*Neisseria meningitidis* requires iron, and in the absence of iron alters its gene expression to increase iron acquisition and to make the best use of the iron it has. During different stages of colonization and infection available iron sources differ, particularly the host iron-binding proteins haemoglobin, transferrin, and lactoferrin. This study compared the transcriptional responses of *N. meningitidis*, when grown in the presence of these iron donors and ferric iron, using microarrays.

Specific transcriptional responses to the different iron sources were observed, including genes that are not part of the response to iron restriction. Comparisons between growth on haemoglobin and either transferrin or lactoferrin identified changes in 124 and 114 genes, respectively, and 33 genes differed between growth on transferrin or lactoferrin. Comparison of gene expression from growth on haemoglobin or ferric iron showed that transcription is also affected by the entry of either haem or ferric iron into the cytoplasm. This is consistent with a model in which *N. meningitidis* uses the relative availability of host iron donor proteins as niche indicators.

Growth in the presence of haemoglobin is associated with a response likely to be adaptive to survival within the bloodstream, which is supported by serum killing assays that indicate growth on haemoglobin significantly increases survival, and the response to lactoferrin is associated with increased expression of epithelial cell adhesins and oxidative stress response molecules. The transferrin receptor is the most highly transcribed receptor and has the fewest genes specifically induced in its presence, suggesting this is the favoured iron source for the bacterium. Most strikingly, the responses to haemoglobin, which is associated with unrestricted growth, indicates a low iron transcriptional profile, associated with an aggressive phenotype that may be adaptive to access host iron sources but which may also underlie the lethal features of meningococcal septicaemia, when haemoglobin may become a major source of iron.

## Introduction

*Neisseria meningitidis* is normally a commensal of the nasopharynx, present in 5–15% of the population [1]. However, in some cases, colonization can progress to disseminated infection, septicaemia, and/or meningitis. Almost all organisms require iron for growth, but for bacteria that colonize humans this is challenging because free iron concentrations are restricted to between 10^−9^–10^−18^ M in the host [2], [3], levels far below that required for growth. *Neisseria* spp. require iron and show a response to low iron conditions similar to most other bacteria, including an increase in the expression of iron uptake proteins, a decrease in iron containing proteins, including the electron transport chain (ETC) proteins, and the production of several virulence factors including the IgA protease and capsule [4], [5].

The host also requires iron for the proper functioning of its own cells and uses iron-binding proteins to restrict, use, and transport iron in different sites. These include haemoglobin (Hb), transferrin (Tf), lactoferrin (Lf), and ferritin. Each of these proteins has a particular function and as such is normally associated with a particular niche within the human body. Tf is predominantly found in serum and in intercellular serous exudates. Tf is able to bind iron very effectively at neutral pH but releases its iron at low pH and humans use this property to transport iron with Tf [6]. During infection, Tf levels in serum decrease in a process called hypoferraemia in which recycling of Tf to the cell surface is reduced [7]. Tf is also present at high concentrations in the cerebrospinal fluid (CSF) and Tf concentrations may increase in the CSF during infection [8], [9]. Lf is most commonly found in secreted fluids (tears, milk, and bile) and on mucosal surfaces [10], where it has a bacteriostatic effect through its ability to sequester iron. Lf, and its N-terminal cleavage product lactoferricin, is bactericidal, damaging outer membranes and causing lipopolysaccharide (LPS) release and pore formation in several Gram negative species, including *N. meningitidis* [11]–[14]. Other non-bactericidal mechanisms by which Lf prevents infection include: induction of twitching motility preventing biofilm formation in *Pseudomonas aeruginosa* infections, and acting as a serine protease to cleave bacterial surface proteins including the Hap adhesin and IgA protease from the surface of *H. influenzae* [15], [16].

Haem is rarely found free in serum and it is usually complexed to one of several haem-binding proteins, including Hb and haemopexin. Hb is the major oxygen and carbon dioxide transport molecule in humans and is the highest affinity haem binding protein. Hb is usually contained within erythrocytes, which prevent its interaction with extracellular bacteria, but spontaneous haemolysis of erythrocytes does occur, resulting in serum Hb concentrations of approximately 80–800 nM [17], where it is rapidly complexed by haptoglobin (Hp). Serum Hp is present at sufficiently high concentrations that it rapidly complexes free Hb. In addition, there are other reservoirs of iron within the body, of which ferritin is the largest, binding 15–20% of the total body iron intracellularly.

**Figure 1:**
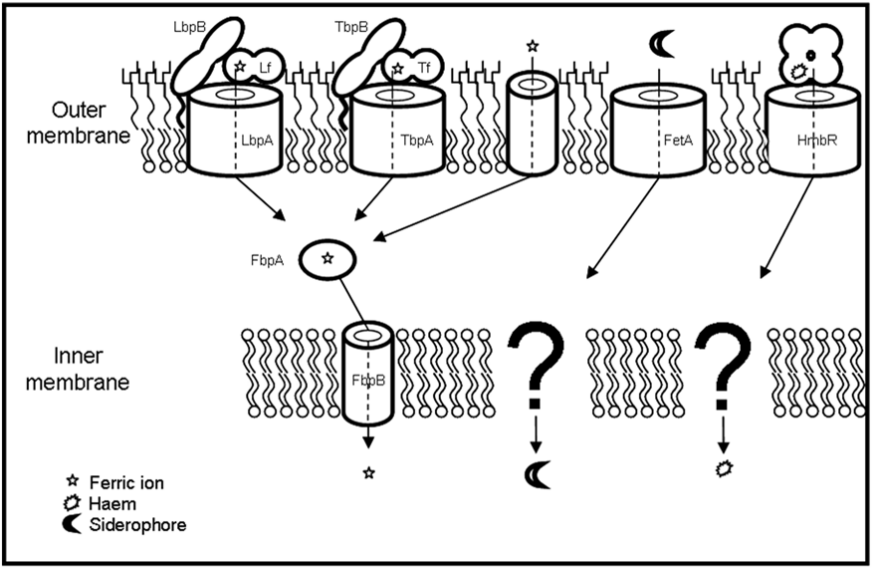
Mechanisms of iron acquisition from the host by *N. meningitidis* MC58. Ferric iron can be acquired directly, or obtained from transferrin or lactoferrin via the cognate receptors, TbpAB and LbpAB respectively [18], [99]. Haem can be acquired from haemoglobin via the haemoglobin receptor; HmbR [25]. Xeno-siderophores may also be internalized through the FetA receptor [100]. Transport through the inner membrane is via the FbpABC proteins for ferric iron [34]–[36], and by as yet undefined mechanisms for the other iron sources.

Most bacteria secrete low molecular weight compounds called siderophores that complex iron. The ferri-siderophore is then internalized by specific receptors in the outer membrane of the bacterium. *N. meningitidis* is not believed to synthesize siderophores, but produces several surface receptors that are able to bind and remove the iron or haem from host iron binding proteins: Tf, Lf, Hb, and Hb-Hp complexes [18]–[21]. *Neisseria* spp. are also able to internalize as yet undefined siderophores produced by other bacteria [22]. *N. meningitidis* strain MC58 does not possess the Hb-Hp receptor, but produces TbpAB, for Tf uptake, LbpAB, for Lf uptake, and HmbR, which binds and removes haem from Hb [23]–[26]. These proteins are all up-regulated in low-iron conditions and the expression of LbpAB and HmbR are also phase variable [27], [28]. All three receptors use energy derived from the TonB protein to strip the metal from the host iron-binding protein [29]–[33]. LbpAB and TbpAB internalize ferric iron from Lf and Tf respectively [34]–[36], while HmbR internalizes haem into the periplasm from Hb. The neisserial iron uptake systems are summarized in Figure 1.

During colonization and invasion *N. meningitidis* will encounter the different iron sources, Tf, Lf and Hb, in different niches. In the nasopharynx Lf will be the principal iron-binding molecule. However, upon dissemination into the bloodstream Lf is scarce, while Hb and Hb-Hp concentrations are much higher. Tf is present in the bloodstream, and also in the CSF. Tf may be available intracellularly, although there is some evidence that ferritin, rather than Tf, is the primary intracellular iron source [37], [38]. While previous studies have focussed upon the importance of the relative abundance of iron, we hypothesized that the specific iron source available may represent a niche indicator, signalling for the expression of genes important for adaptation and survival in the different microenvironments within the host.

The availability of different iron sources to *Neisseria* spp., and the consequent use of these, during infection has been described previously [39], although no mention was made of alternative protein expression as a result of the different iron donors used. Many species of bacteria respond to the presence or absence of iron by regulating multiple genes associated with iron uptake, as well as virulence and metabolism [4], [5], [40], [41]. There are also some reports of bacteria altering expression of proteins in response to the iron-binding protein from which iron is acquired. The best characterized example of this is the response to the binding of ferric citrate by FecA in *E. coli*. In *E. coli*, ferric citrate acts as an inducer of the *fecABCDE* operon [42], mediated through an extracytoplasmic function (ECF) sigma factor [43]–[45]. Also, when the HasA haemophore of *Serratia marescens* is bound to Hb, it can bind to the HasR outer membrane receptor and transmit a signal, via an ECF sigma factor, that activates transcription of the *hasR* gene [46], [47]. In *N. meningitidis*, one study suggests that HmbR may be up-regulated in the presence of haemoglobin [25], but this has not been characterized further.

Only one previous study, using *Pasteurella multocida*, has considered differing transcriptional responses to specific iron donor sources; using Tf, Hb, ferritin, and ferric citrate. This study reported that approximately 12% of all genes in *P. multocida* were regulated differently in the comparisons of these different iron sources [48]. The majority of genes with altered expression were of unknown function and those with an annotated function were involved in iron uptake and modification of the cell surface. However, the data and methods are not available for this study (due to citation of non-existent web pages), it is not clear that more than one biological replicate was tested, and the iron sources were acquired from a range of species. Because most iron acquisition proteins are species specific, and there were large changes in the expression of the iron stress regulator, Fur, this study is extremely difficult to interpret and much of the observed responses may simply be attributable to differing iron availability.

In this study we have tested the hypothesis that the iron donors have the potential to act as niche indicators by determining the expression profiles of *N. meningitidis* in the presence of different human iron donor molecules and, using the same methods, have also compared these to the responses to iron restriction, in order to characterize the responses specific to each. In order to isolate the effects of the individual iron donors this has been done using comparisons of a series of pair-wise comparisons using concentrations that support similarly unrestricted growth. We have observed differences that are consistent with the model that the iron donor molecules act as specific niche indicators, and confirmed that these differences are mostly dependent upon the iron donor and not the iron molecule that would be imported across the inner membrane. The expression profiles predicted increased serum resistance in the presence of haemoglobin, which was tested using serum killing assays, which confirmed the presence of a more serum resistant phenotype.

## Results and Discussion

### Growth of *N. meningitidis* on RPMI-based medium agar

An iron-free medium was used which enabled the study of responses to low iron without the use of iron chelators, which we wished to avoid because these also alter the availability of other metal ions. RPMI-based medium (RBM) was essentially iron-free with RPMI containing no iron, and the agar containing less than 0.006% iron. *N. meningitidis* strain MC58 was able to grow on this medium for one passage, but not two, without an iron supplement, although more poorly than if an iron supplement is added. The ability to grow for one passage without iron indicates a capacity for storage and use of intracellular iron reserves. To ensure that bacteria were exclusively using the specific intended iron sources and to remove the bacterial iron stores, the bacteria were therefore passaged on RBM agar containing desferal prior to growth on the supplemented medium (without desferal).

### Findings based upon the pair-wise comparisons of individual iron donors

**Figure 2:**
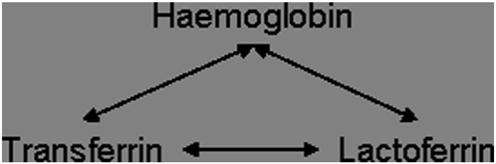
Microarray experimental design to identify the transcriptional effects of growth on the iron sources haemoglobin, transferrin, and lactoferrin.

Comparison of meningococcal transcriptional differences during growth on the three iron sources Tf, Lf, and Hb was performed as shown in Figure 2.

Pair-wise comparisons using single iron donors were performed so that the effects of individual donors could be distinguished from the nature of the iron imported, and so that overlapping responses could be identified. Six independently processed biological replicates, grown on different days from a common stock, were used for each arm of the experiment. The numbers of genes that differed by more than 1.5 fold in each comparison are shown in Table 1. A parallel study, using the same methods and media, was performed to identify the responses to iron availability and is described in Dataset S1. This was done so that the effects of the specific donor molecules could be distinguished from those associated with differences in the efficiency of iron acquisition from them.

**Table 1:** Numbers of genes changed greater than 1.5 fold in comparisons between Hb, Tf and Lf.

	Cyber-T *p*<0.05^a^		
Comparison†	Total	of which not present in the + / − iron response*	Consistent¥
Tf *vs.* Hb: Tf	97	42	36
: Hb	180	82	58
Hb *vs.* Lf: Hb	105	51	21
: Lf	88	63	25
Tf *vs.* Lf: Tf	35	28	16
: Lf	11	5	1

† The numbers correspond to genes up-regulated in the presence of the particular iron source in each comparison.

a These genes had a significance of *p*<0.05 in the CyberT test.

* This column shows the genes that are only changed in response to the iron source. The genes that are regulated as a result of differential iron uptake are excluded from this column. This is described in more detail in the text.

¥ This column shows the number of genes that were changed in one direction in all biological replicates.

Despite comparable and good growth on solid media (and in liquid media (data not shown)), bacteria grown with Hb as a sole iron donor always showed a transcriptional profile that includes changes typical of growth in low iron, e.g. increased transcription of iron uptake proteins, while those grown on Tf always showed a transcriptional profile typical of growth in high iron, e.g. increased transcription of iron storage proteins. Expression profiles from equivalent growth on Lf showed an intermediate phenotype suggesting that the iron availability was greater than from Hb, but less than from Tf. Therefore, there is not a simple relationship between the iron required to achieve similar bacterial growth and the cell’s sensing of available iron. Changes in transcription in a direction characteristic of responses to low iron were excluded from the analysis directed at identifying genes that are specifically regulated in response to each of the iron donors. This does not mean that these are not regulated differently in response to a particular iron source, but in this experiment it is impossible to distinguish whether they are primarily responsive to the iron availability, or the iron donor. Following exclusion of these genes, 124 and 114 genes were found to be differentially regulated in the comparisons of Hb with Tf and Lf respectively, and 33 genes were differentially regulated in the comparison of Tf and Lf.

The results of the microarray experiments can be interactively interrogated in an on-line graphical GBrowse database at http://www.compbio.ox.ac.uk/data.shtml (for purposes of review username: MC58_iron; password: GBReview), where the fold ratio, number of observations for each gene, Cyber-T *p*-values, and the microarray probe locations can be visualized, and searched using chromosomal locations, gene names, and annotations. The results from each experiment can be viewed individually or in combination to make user-selected comparisons.

The results of the individual comparisons will be described before summarizing those genes that are responsive to the presence of each individual iron source. In each case, for simplicity, genes will be described in terms of the iron source with which their expression is greatest, and therefore in terms of induction. However, it should be borne in mind that it is possible that the expression of some genes may be inhibited in the particular conditions and that the mechanistic direction of regulation may be the reverse, i.e. de-repression. To aid reading, the fold ratios are contained in the Supplementary tables.

### Comparison of *N. meningitidis* grown on Haemoglobin or Transferrin

Hb and Tf are both present in serum. Hb is almost exclusively present in the bloodstream and serous exudates, whereas Tf is also present in the CSF and is possibly available intracellularly. Therefore, both Hb and Tf will be encountered once local tissue or cellular invasion has occurred. Tf might be predicted to act as an indicator of growth within the cells and tissues of the host, rather than on the epithelial surface, while Hb would be primarily indicative of a location with a serous exudate or within the vascular compartment. 42 and 82 transcripts were increased in the presence of Tf or Hb, respectively. This included 78 annotated genes, with functions predominantly associated with changes in the cell surface and the expression of secreted proteins. The changed genes are listed in Tables S1 and S2.

#### Cell surface and extracellular proteins up-regulated in the presence of Hb

Transcripts for several putative virulence genes were increased in the presence of Hb. *N. meningitidis* possesses a paralogous family of RTX protein homologues that contain a repeat motif present in exotoxins in many other Gram-negative species, including HlyA in *E. coli* and RtxA in *Vibrio* spp. [49], [50]. Most of these have altered expression in the presence and absence of iron, but two members of this family show changes solely in the presence and absence of Hb: *frpC* (NMB1415) and *nmb1409*. The Hb response was also associated with an increase in the transcription of a haemagglutinin/haemolysin related protein (NMB1768), and *fhaB* (NMB0511), which encodes a filamentous haemagglutinin. If these predicted functions are correct this suggests an increase in erythrocyte binding and lysis, which may play a role in releasing additional Hb, although no haemolytic activity has been previously associated with *frpC* [51], [52].

*pilE*, the major constituent of pilus, and at least one member of the *opa* family, form part of the Hb-associated response. The meningococcal pilus is the primary initial adhesin due to its ability to project beyond the capsule [53]. However, the expression, assembly, retraction, and modification of pili is complex and is dependent upon the coordinated expression of multiple genes, such that an increase in the expression of any one of these genes, even *pilE*, cannot be directly interpreted to specifically indicate a change in pilus abundance. There are four *opa* loci in the meningococcal genome and the probe is unable to differentiate between these loci. The Opa proteins have different binding affinities to heparan sulphate and CD66-family proteins and are able to cause intimate adhesion between meningococci and several cell types, as well as influencing binding of capsulate bacteria [54]–[60]. There was also up-regulation of a putative cell-binding factor (NMB0345).

There were several other Hb-associated changes in the expression of cell surface components, including increased expression of *rfaC*, which encodes heptosyltransferase I, and a glycosyltransferase (NMB0624). An *rfaC* mutant is attenuated in an animal model potentially through affecting complement binding and serum-mediated killing [61]. The genes in the capsule locus were also up-regulated, which would be predicted to increase serum resistance, and is functionally consistent with expression of the pilus which extends through it to act as an adhesin. Because the capsule genes were also up-regulated in the absence of iron they are not included in the results table.

#### Responses of Fur and Fur-associated genes with Hb

Regulators showing differential expression in comparisons of all of the iron sources tested are summarized in Table 2. Fur, which is the most studied iron-responsive regulator in *Neisseria* spp., was up-regulated in the presence of Hb. At 1.7-fold, it is induced more in the comparison of Hb and Tf than it is in the presence and absence of iron (Dataset S1). This is an unexpected finding, which is discussed later. Transcription of the nitric oxide reductase (*norB*) was increased, which is normally controlled by NsrR in response to nitric oxide [62]. It has been shown that *norB* is activated by Fur [63] and the Hb-associated response is generally associated with genes repressed by Fur, so the underlying basis for this change is unclear.

**Table 2:** Transcriptional regulators changed in one or more comparisons.

Regulator	Fold Ratio Hb/Tf	Fold Ratio Hb/Lf	Fold Ratio Tf/Lf
Nitrogen regulation protein NtrY (NMB0114)	No data	NS	2.7, *p = *0.008
Transcriptional regulator, TetR family (NMB0810)	0.5, *p*<0.001	0.6, *p = *0.002	NS
Transcriptional regulator, AraC family (NMB1967)	2.7, *p*<0.001	NS	NS
Fur (NMB0205)	1.7, *p = *0.010	1.8, *p = *0.008	NS
FarR (NMB1843)	1.7, *p = *0.002	1.3, *p = *0.037	NS
NarQ (NMB1249)	1.6, *p = *0.011	1.6, *p = *0.034	NS
Repressor protein (NMB0556)	0.5, *p*<0.001	0.5, *p = *0.003	NS
Transcriptional regulator (NMB1007)	NS	1.5, *p = *0.040	NS

NS: Not significant.

#### Cell surface and extracellular proteins up-regulated in the presence of Tf

The responses to the presence of Tf, compared to Hb, are shown in Table S2. These included increases in transcription of major surface proteins *porB* and *hsf*. *pilT-1* was up-regulated, while *pilE* was down regulated, suggesting decreased surface pilus expression conducive to closer bacterial-host cell interactions. There was also up-regulation of a different haemolysin (NMB2091) to that seen in response to the presence of Hb, so there appears to be a reciprocal expression of specific haemolysins / membrane toxins in response to these two iron donors.

In the presence of Tf there was up-regulation of the O-antigen acetylase (NMB0285), which is implicated in preventing phosphoethanolamine (PEA) binding to the β-chain of neisserial LPS [64]. PEA is important for preventing binding of C4 binding protein to LPS [65], and it has been suggested that this substitution may also alter the ability of the bacterium to adhere to cells [64].

#### General metabolism transcripts up-regulated in the presence of Tf

There was a general increase in the components of the TCA cycle including: citrate synthase (*gltA*) and 2-oxoglutarate dehydrogenase (*lpd*), and also in the ETC protein *cytC* ; suggesting greater oxidative respiration. *adhP*, a propanol preferring alcohol dehydrogenase which is thought to be involved in non-oxidative respiration was also increased. Pathways by which alcohols may be synthesized or utilized have not been defined in *Neisseria* spp., but *adh*P is one of the most highly transcribed genes in the cell, with transcript abundances often similar to those of the ribosomal RNA operons, suggesting that this protein may have an ancillary function.

#### Summary of Hb *vs.* Tf comparison

The presence of Hb induces genes that, based upon their currently understood functions, are likely to aid intravascular survival and increase local cellular damage that may be central to the toxicity of this species during septicaemia. There was increased transcription of the adhesins: pilus, Opa, and *fhaB*; and toxins: haemolysin/haemagglutinin (NMB1768), and the RTX toxin homologues *nmb1409* and *nmb1415*. There was also induction of genes that may foster intravascular survival through evasion of complement: including increased transcription of *rfaC* which synthesizes a sialylatable structure, and reduction of the complement targets PorB and surface-bound PEA (NMB0285).

Tf is associated with a more metabolically active physiological state that generally reflects a lack of iron limitation, including: a more active TCA cycle and respiratory chain. The phenotype also suggests a closer association with cells through reduced pilus expression and alterations in adhesins and LPS.

### Comparison of *N. meningitidis* grown on Haemoglobin or Lactoferrin

While Hb is present in blood and serous exudates Lf is present on the mucosal surface, and is not normally encountered in the bloodstream except following granular release by neutrophils. If Lf acts as a niche indicator, one might predict an association between the presence of Lf and genes required for colonization of the nasopharynx. The genes differentially regulated by Hb and Lf are shown in Tables S3 and S4. 51 transcripts were more abundant in the presence of Hb and 63 in the presence of Lf.

#### Cell surface and extracellular proteins up-regulated in the presence of Hb

Consistent with the findings in the Hb *vs.* Tf comparison, Hb was associated with increased transcription of the FrpA/C gene *nmb1409*, the haemagglutinin NMB1768, and a putative cell-binding factor (NMB0345) suggesting a role for these proteins in the intravascular niche. In addition, transcripts for a type IV pilin-related protein and the major surface protein RmpM were increased.

#### RpoH response induced in the presence of Hb

Transcripts for *rpoH* and other transcripts within its regulon, as defined in *N. gonorrhoeae* [66] were increased, including: NMB0977, a putative modulator of drug activity, *secY*, and the protein folding chaperones: *grpE* and *groES*. Transcripts for 10 ribosomal proteins were increased, including six from the ribosomal operon, suggesting that there is increased protein biosynthesis in the presence of Hb. This is consistent with the expression of *secY* and the chaperones regulated by *rpoH*. We interpret the alteration in *rpoH* to be associated with increased normal protein processing, rather than a stress response, as other components of the stress response, such as *htpX*, remain unchanged.

#### Cell surface and extracellular proteins up-regulated in the presence of Lf

Six genes considered to effect bacteria-host interactions were induced in the presence of Lf compared to Hb. These include *hsf* and *porB*. *hsf*, also called NhhA, is a homologue of the *hia* and *hsf* genes from *Haemophilus influenzae* [67]. Hsf is a member of a family of trimeric autotransporters that bind to the extracellular matrix, including laminin and heparan sulphate [68], and host receptors, allowing intimate attachment to epithelial cells (reviewed by Cotter *et al.* [69]). PorB is important in epithelial cell adhesion and immune system evasion. When expressed in *N. gonorrhoeae* PorB can cause actin rearrangement in epithelial cells leading to increased epithelial cell adhesion [70]. PorB can also inhibit the actions of neutrophils by preventing granule release and phagocytosis [71], but it is also targeted by the complement activator mannose binding lectin [72], suggesting its expression needs to be minimal within blood, except in the presence of neutrophils. *mafB* and NMB1909, which encode Maf family proteins, were also up-regulated in the presence of Lf. The Maf family proteins are a family of adhesins, with multiple silent loci, that bind to glycolipids on host cells [73]. Expression of *pilP* was up-regulated, which is important for PilQ multimerization and PilC-mediated pilus assembly [74], [75]. Finally, up-regulation of the phase variable *lgtG* gene was observed, which adds a glucose to the second heptose of meningococcal LPS [76]. Expression of *lgtG* has differing effects upon serum susceptibility depending upon the rest of the LPS phenotype and encapsulation [77]. Taken together, these changes appear to be adaptive for the surface niche, and the presence of neutrophils, with which Lf would be associated.

#### DNA modification and repair transcripts up-regulated in the presence of Lf

There are several DNA repair-associated genes that were up-regulated in the presence of Lf, suggesting a functional role for the gene products in the presence of Lf. The transcript for *xseA*, which encodes the large subunit of exodeoxyribonuclease, a mismatch repair enzyme, was increased. A role for this enzyme in host interaction is suggested by induction of *xseB* in *N. meningitidis* strain Z2491 in response to cell contact, but in this previous study *xseA* was unchanged [78]. However, in this study *xseB* is unchanged. Two other DNA repair genes were also up-regulated, *xthA*, which encodes exodeoxyribonuclease III, and *recN*. The up-regulation of *recN* suggests a pre-adaptive response to DNA damage, perhaps related to oxidative stress. Stohl *et al.* reported that *recN* was the only DNA recombination and repair gene up-regulated in the presence of hydrogen peroxide [79]. Since *recN* was down-regulated in the presence of Hb compared to both Tf and Lf, it may be that this reflects hydrogen peroxide reacting with ferric ions to produce free-radicals, which can subsequently cause double-strand DNA breaks. Alternatively, it may represent an adaptive response to a greater oxidative stress in the niches in which these iron-binding proteins are found. Delany *et al.* [80] reported that *recN* is Fur-regulated, but it should be noted that in this study the change was opposite to that of the rest of the indicators of iron restriction / Fur activity previously reported, indicating specific and additional regulation. Lf would not be expected to directly affect DNA, but clearly there is a coordinated response of these chromosomally remote, functionally related genes.

#### Metabolic transcripts up-regulated in the presence of Lf

Broadly, the metabolic changes are similar to those seen in association with Tf when compared to Hb, including induction of genes associated with glycolysis, TCA cycle, and electron transport, as well as *adhP*. This suggests that the cell is more metabolically active with an increase in oxidative respiration in the presence of Lf.

#### Summary of Hb *vs.* Lf comparison

The response to Hb is indicative of a phenotype adaptive for intravascular survival, which is broadly similar to that seen in the comparison of Hb and Tf. The presence of Lf produces a response that is probably adaptive for cell surface interactions, including factors affecting increased adhesion to and remodelling of epithelial cells: *hsf*, *maf*, *porB*, and *pilP*. DNA modification and repair genes, such as *xthA*, *recN*, and *xseA*, are also up-regulated, but their functional significance is currently unknown.

### Comparison of *N. meningitidis* grown on Transferrin or Lactoferrin

Tf is likely to be present in most niches once invasion has occurred, while Lf is largely confined to the mucosal surfaces, including the nasopharynx, and neutrophil granules. Tf- and Lf-associated transcription profiles are broadly similar, with only 33 genes that were differentially regulated, of which only 17 were similarly changed in all biological replicates in which they were measured. The differentially regulated genes are shown in Tables S5 and S6. The specific response to either of these iron sources is far smaller than that associated with Hb.

The differences are predominantly in metabolic genes. In addition, the transcript for the IgA protease was increased in the presence of Tf; which cleaves both IgA and LAMP-1, and is potentially adaptive to either surface or intracellular niches depending upon which of these substrates is targeted [81]–[83]. Tf was associated with the expression of a TonB-dependent receptor (NMB1497), which has homology to haem and Tf binding proteins from several species, and may be indicative of acquisition of an additional iron source in a niche associated with Tf. If this is associated with an intracellularly adaptive phenotype this receptor might also be hypothesized to be related to acquisition from bacterially induced ferritin turnover [38]. Note, this is a different TonB-dependent receptor to TdfF, required for intracellular iron uptake, as described by Hagen and Cornelissen [84].

The expression profiles associated with Lf and Tf may be broadly similar because they both donate ferric ions into the cytoplasm rather than haem, which is addressed below. However, there are a substantial number of differences in the comparisons of Hb-Lf and Hb-Tf that are not seen when comparing Lf and Tf directly. A proportion of these differences will be attributable to the fact that one or other iron source is associated with intermediate phenotypes so that the differences in a single comparison do not achieve the arbitrary fold-ratio cut-offs used in analysis. Other differences are likely to be attributable to interacting effects of the transporter used, the iron molecule imported, and other more complex primary and secondary responses, for example due to changes in major surface porins such as PorB and RmpM.

### Comparison of *N. meningitidis* grown on Haemoglobin *vs.* Ferric ions

Ferric iron, as stripped from Tf and Lf, is taken up by the Fbp ABC transporter system, and is thought to be the major mechanism by which iron from Tf and Lf enters the cell, while haem is transported into the cytoplasm by a different currently unknown mechanism [34]. In order to determine whether the distinct responses associated with Hb were due to the uptake of ferric iron or iron bound in haem into the cytoplasm, rather than Tf-, Lf-, and Hb-specific uptake systems, the transcriptional profiles of *N. meningitidis* MC58 grown on Hb and ferric chloride were compared. The results of this comparison are shown in Tables S7 and S8.

Twenty-three genes were significantly up-regulated in the presence of Hb compared to ferric chloride, which is considerably fewer than the 82 and 51 genes up-regulated in the presence of Hb in the Hb-Tf and Hb-Lf comparisons, respectively. Only three of these 23 genes (NMB0036, NMB1844, and NMB1768 (haemagglutinin/haemolysin-related protein)) were up-regulated in the presence of Hb in both Hb-Lf and Hb-Tf comparisons. A further three of the 23 genes (NMB0120, *mafB*, NMB1746) were up-regulated in the Hb-Tf comparison but not the Hb-Lf comparison. In contrast, 55 transcripts were increased in the presence of ferric chloride compared to Hb; nine of which were decreased in the presence of Hb when compared to Tf and Lf (*porB*, *atpF*, *hisS-1*, NMB0786, NMB1221, NMB1436, NMB1898, and an unannotated gene between NMB1000 and NMB1001). There are four transcripts seen up-regulated by ferric iron that were also up-regulated when grown on Tf compared to Hb (*leuC*, *argB*, *fixS*, and NMB0820) and five transcripts that were up-regulated when grown on Lf compared to Hb (*rpsL*, NMB0048, NMB0247, NMB1049, and NMB2137).

Expression of both of the twitching motility proteins, *pilT-1* and *pilT-2*, was up-regulated in the presence of ferric iron. These genes encode proteins involved in pilus retraction, associated with movement [85], and twitching motility is induced by Lf in some bacterial species, preventing biofilm formation [15]. *pilT-1* was down-regulated in the presence of Hb, compared to Tf (1.5-fold, *p = *0.02), while *pilT-2* was down-regulated in the presence of Hb compared to Lf (1.5-fold, *p = *0.04). In contrast, both of these genes were down-regulated in response to Hb compared to ferric chloride (*pilT-1*; 1.8-fold, *p*<0.01; *pilT-2*; 1.6-fold, *p*<0.01). This suggests that the availability of ferric iron or Hb may influence both *pilT* genes and also that Tf and Lf have specific inducing effects for the individual alleles.

Only a small minority of the responses in the Hb vs. Lf and Hb vs. Tf comparisons could be attributable to the form of iron taken up to cell. A similar picture is seen when the comparison is reversed. Only twenty-four (31%) of the differences seen comparing Hb and ferric iron are also different in the comparisons between Hb and either Tf or Lf. This indicates that the majority of differences seen in the donor comparisons are specific to the donor molecule, rather than to the effects of importing ferric iron. This suggests that there must be at least two points at which signalling occurs to indicate the presence of an iron source. At the outer membrane there must be recognition of the iron donor molecule with signalling occurring via the outer membrane channel or an accessory protein. In addition, there must be recognition of the ferric iron or haem either in the periplasm or cytoplasm with the concomitant control of some of the same genes and many extra genes. This second form of signalling may be through Fur as well as other unidentified proteins.

### Comparison of *N. meningitidis* grown on Haemoglobin *vs.* Haemoglobin and Transferrin

If Tf, or ferric ion donors, were preferred iron donors, it could be argued that the Hb response may represent a metabolic or stress response to the lack of a preferred donor, rather than being a specific response to the Hb or haem taken up. To address this, the transcriptional profiles of *N. meningitidis* MC58 grown on RBM agar supplemented with Hb alone or both Hb and Tf were compared (Hb *vs.* Hb&Tf).

The results of these comparisons are presented in Tables S9 and S10. A summary is displayed in Table 3. This Table summarizes the numbers of transcripts differing by greater than 1.5-fold in the Hb *vs.* Tf, and Hb *vs.* Hb&Tf comparisons and highlights the number of genes that are similarly or differently changed between the two comparisons. Only 22% and 7% of the transcripts with higher and lower expression, respectively, had the same altered expression in association with the presence of Tf. In other words, the expression profiles of growth on Hb and growth on Hb&Tf were similar, and most of the responses associated with Hb in the pair-wise comparisons are also present when Tf is added to the medium.

**Table 3:** Summary of the numbers of genes that are changed in either the Hb *vs.* Tf comparison or the Hb *vs.* Hb&Tf comparison and the observed response in other comparison.

	Hb *vs.* Hb and Tf			
**Hb ** ***vs.*** ** Tf**		Up	Unchanged	Down
	Up	12	16	8
	Unchanged	89	-	35
	Down	28	34	5

This table highlights the limited overlap between the observed responses of the Hb *vs.* Tf comparison and the Hb *vs.* Hb&Tf comparison.

Before this comparison was preformed, the transcriptional iron restriction response might have been attributed to the 5-fold lower iron concentration in the Hb-containing medium (despite similar growth). However, both cultures include similar iron restriction responses, even when Tf is added, and the combined medium contains the same total concentration of iron as the Tf-only medium (and 5-fold more than the medium with Hb alone). This indicates that the presence of Hb inhibits the normal response to iron concentrations, or acquisition from other donors. This is consistent with our model that the majority of genes changed in the Hb *vs.* Tf comparison are responding specifically to the iron source, rather than reflecting differences in growth rate or other factors.

### Findings based upon the presence of individual iron donors

Genes that are commonly changed in response to the presence of an iron donor in the individual pair-wise comparisons represent candidates most likely to be directly responsive to each donor, and the responses were compared to determine their common components. In this combined analysis transcripts are described as increased or decreased with respect to the presence of each iron donor, and area summarized in Tables S11 and S12 for Hb, Tables S13 and S14 for Tf, and Tables S15 and S16 for Lf.

Only three genes are significantly increased in the presence of Tf (excluding those that are part of the iron presence/absence response). Two are hypothetical proteins and the other is *leuC*; part of the leucine biosynthetic pathway. The only down-regulated transcript is *fbpA*; part of the iron uptake ABC transporter and likely to be part of the iron presence/absence response. Given the level of typical inter-culture variation in gene expression, it is plausible that there is no specific response to Tf as an iron donor, which is consistent with its wide distribution within the host. One of the most consistent aspects of iron restriction responses is increased expression of transferrin acquisition systems, which would be adaptive in most or all naturally encountered host microenvironments.

Microarray data obtained using the dendrimer labelling method used in this study is semi-quantitative [86], [87], and single channel data can be obtained by correcting for between-channel cross-talk and scaling the data, to compare transcript abundance across different microarrays and experiments. Single channel analysis indicates that the transcript levels of the Tf channel are 3.4 times greater than the membrane components of either the Lf or Hb receptors. Given the widespread presence of Tf, the lack of a Tf-specific transcriptional response, the observation that when iron is limiting *tbpB* is the most up-regulated gene and that when expressed it has the highest transcript abundance of any uptake channel, we propose that *N. meningitidis* may preferentially use Tf as the primary source of iron within the host. While the dominant phenotype of Hb in the Hb vs. Hb&Tf comparison suggests that Hb, and maybe also Lf, are used primarily as niche indicators and as secondary sources of iron when Tf is not available.

Many of the surface adaptive adhesins that differed in the Hb *vs.* Lf comparison are not seen in the combined analysis because the Lf and Tf phenotypes are more similar to each other, perhaps due to the form of iron acquired, and thus are not seen in both comparison. The most striking changes in the combined analysis of the response to Lf are the up-regulation of the major surface protein PorB and the DNA repair protein RecN, which are discussed previously.

The greatest specific response (87 genes up- or down-regulated) is to the presence of Hb. Transcription of several genes affecting host interaction are consistently associated with the presence or absence of Hb. These include increases in: a putative cell binding factor (NMB0345), a haemagglutinin/haemolysin related protein (NMB1768), a member of the FrpAC operon (NMB1409), and a pilin related protein of unknown function; and decreases in: *hsf*, a Maf family gene (NMB1909) and *porB*, all of which influence epithelial cell attachment or epithelial cell actin rearrangement [68], [73], [88]. Overall, the response to Hb reduces the expression of genes associated with epithelial cell adhesion, and increases the production of toxins and alternative cell-adhesion molecules. There is also reduction of oxidative stress responses, the TCA cycle, and electron transport chains. That there is an evolved response and phenotype adaptive to Hb containing / intravascular niches is consistent with the, largely neglected, observation of meningococcal bacteraemia in about 50% of febrile case contacts, who themselves had no specific features of meningitis or meningococcal septicaemia [89].

### Serum killing assays

**Figure 3:**
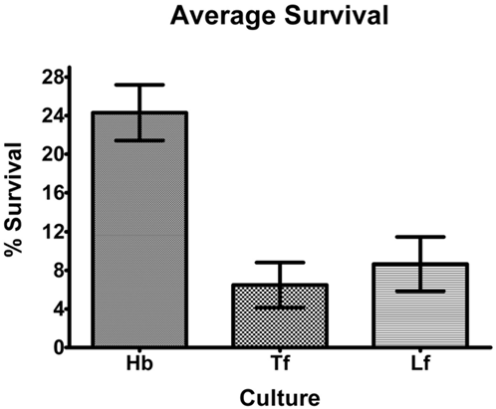
Serum bactericidal assay showing the effects of growth of *N. meningitidis* on RBM agar containing Hb, Tf, or Lf and subsequent treatment with 5% NHS. The percentage survival is the average number of CFUs remaining compared to controls in which complement was heat inactivated. Values are the mean of at least seven determinations in triplicate±SEM.

The transcriptional profile associated with Hb suggested that the bacteria synthesizes proteins that are adaptive to iron acquisition and survival within the vascular compartment; such as increased expression of toxins annotated as haemolysins, altered cell adhesion expression, and reduced expression of complement targets such as PorB and the sialylated LPS dependent upon RfaC. To test the prediction of increased serum resistance, serum killing assays were performed. *N. meningitidis* MC58 were grown on RBM agar containing: Tf, Lf, or Hb and were then incubated in 5% human serum for one hour. Each assay was repeated between seven and nine times, and the average percentage survivals are shown in Figure 3. The mean survival following growth on Hb (24.3%±8.7) was significantly higher than when grown on Tf (6.5%±6.2, *p*<0.0005) or Lf (8.7%±8.0, *p*<0.002). There was no difference between serum resistance of bacteria grown in the presence of Tf or Lf (*p* = 0.57). This confirmed the predictions of the microarray data. What is shown represents the significant growth and survival advantage of the pre-adapted phenotype, but, because the serum includes both Hb and Tf, rather than the initially defined iron source, this experiment may underestimate these differences.

### Regulation and signalling in response to iron sources

Of the eight transcriptional regulators showing differences in the comparisons (Table 2), four: Fur, *narQ*, a putative TetR family regulator (NMB0810), and a putative repressor protein (NMB0556), were associated with Hb. These regulators are good candidates for controlling the response to particular iron sources. This study does not identify a particular regulator for Lf or Tf. However, the transcript abundance of regulatory proteins is often very low and not all regulator transcripts can be reliably measured in this experimental system, so there may be additional regulators key to these responses.

Fur acts as an autorepressor when it contains its iron core [90], and increased transcription of *fur* suggests that the Fur protein is not functional / iron loaded. Therefore, even though there is sufficient iron in the presence of Hb to support approximately equivalent growth, and far better growth than on unsupplemented media, the cellular iron sensing systems are reporting a physiological lack of iron even greater than was the case in the transcriptional profile of growth in the absence of iron (see Dataset S1). Other aspects of the transcriptional profile associated with Hb confirm this, such as the induction of *tonB*, *exbB*, *exbD*, the ferric iron binding protein *fbpA*, acquisition proteins for Tf and Lf, and a reduction in the bacterioferritin iron storage proteins.

The single-channel data suggests that the levels of Fur transcript when grown in the presence of Tf or Lf are similar to the levels when grown with ferric chloride as the iron source. However, the transcript levels of *fur* when grown in the presence of Hb are higher (by 20–47%) than when grown in the absence of iron in the iron depletion experiment. The apparent inability of *N. meningitidis* to recognize haem iron is paradoxical as it encodes a functional haem oxygenase [91], which releases the iron from haem.

These findings suggest that the iron taken up by the cell from Hb cannot be sensed by the systems that normally control the iron-stringent responses, perhaps because it is unavailable to the sensing system or is in a form that cannot be incorporated into Fur. Although there are specific differences, there are global similarities between the transcriptional profiles of the iron replete *vs.* deplete data, and the Hb *vs.* Lf comparison, and the Hb *vs.* Tf comparison. The correlation coefficients (r^2^) for the log-transformed fold-ratios of genes with a *p*-value of <0.05 are 0.18 and 0.50 respectively (0.06 and 0.13 for all genes). This confirms that a component of the response to Hb may be as a result of differently recognized iron levels.

The apparently inappropriate ‘low iron’ transcriptional response in the presence of Hb may underlie the apparent hypervirulence of the Hb-associated phenotype, and possibly the aggressive nature of invasive meningococcal disease. Hb is likely to be a minor source of iron in normal transmission/colonization-associated niches, and growth under conditions in which Hb is the primary source of iron is physiologically abnormal. The iron-restricted transcriptional profile, in the presence of good growth, suggests that on a molar basis Hb is a preferred iron source, and that the normal sensing systems of the bacterium do not efficiently monitor or are confounded by iron from Hb. Normally, when the cell detects a lack of iron its growth would be seriously restricted, in which an ‘aggressive phenotype’ directed at cellular damage resulting in release of iron from the host would be adaptive, but not normally dangerous due to iron-restricted growth. However, this is not the case when Hb is being used as a significant source of iron. Perhaps the basis of aggressive meningococcal septicaemia is due to the expression of this aggressive phenotype directed to accessing host iron in a setting in which growth is abnormally unrestricted.

Overall, the findings of this study indicate that a coordinated response occurs in response to the presence of both the specific iron donor molecule at the outer membrane, and from the type of iron which is transported into the cell. The cell integrates these signals to generate the overall response to the particular iron source.

### Conclusion

This study has addressed the transcriptional responses to specific host iron donors in *N. meningitidis* strain MC58. In order to achieve this, a novel iron-free medium was used. Culture on this medium demonstrated that sufficient iron is stored intracellularly by *N. meningitidis* to support one overnight passage of the bacterium, which has important implications for studies of iron restriction. It is also consistent with the limited survival of bacteria in mouse models, which require an exogenous iron source, e.g. iron dextran, to induce bacteraemia [92].

The identification of the key factors controlling bacterial responses and host interacting phenotypes is central to understanding the host-bacterial interactions that underlie the different possible outcomes. In this context, determining the key niche-indicators, and subsequently the bacterial responses to these, are fundamental goals of pathogen functional genomics. There are very few indicators that differentiate host niches clearly, and we hypothesized that iron donors were good candidates for this role. The findings of this study are consistent with this model, and the response to Hb appears to underlie an aggressive phenotype that may be central to the severity of meningococcal septicaemia.

We interpret the expression profiles to indicate that transferrin is probably the preferred normal host iron donor, which is associated with what might be considered the ‘steady state’ neisserial phenotype. Lactoferrin serves as an indicator of location on the epithelial surface, and is associated with the expression of genes favouring close approximation of bacteria to the host cell surface. Haemoglobin supports good meningococcal growth, and its presence is associated with the expression of a range of ‘aggressins’ simultaneously likely to reduce immunological clearance and induce local tissue damage. This is supported by serum killing assays that indicate that meningococci grown on Hb, rather than Tf or Lf, survive better in human serum.

Whether this is a specific response to Hb / Hb binding / haem import, or growth when the cell is failing to detect the presence of iron acquisition from Hb and other sources, is currently undefined. Generally, the responses to both Lf and Hb are probably enhanced in the setting of a relative lack of iron acquisition by other means, and we suggest that both Lf and Hb are secondary iron sources, and their importance may be directly related to niche detection. In addition, the Hb and Lf receptors are phase variable, thereby allowing sub-populations to be present that do not respond in the same way to the rest of the population.

## Materials and Methods

### Bacterial strains and growth conditions

*N. meningitidis* strain MC58 *siaD* ^−^ [77] was passaged from the −80°C freezer on GC agar with the Kellogg supplement and ferric nitrate [93] at 37°C with 5% (v/v) CO_2_. To deplete intracellular iron stores, 10–20 colonies were transferred to RBM agar (described below) with 16.6 µM desferal (Sigma) and incubated overnight. Then, 20 colonies were passaged onto RBM agar supplemented with human iron-loaded 10 µM Tf (20 µM Fe), 10 µM Lf (20 µM Fe), 1 µM Hb (4 µM Fe), 1 µM Hb/8 µM Tf, or 20 µM FeCl_3_ (Sigma). These concentrations of iron were chosen on the basis of comparable growth of *N. meningitidis* MC58 *siaD* ^−^ after 16 hours incubation on RBM with each iron source, even though they did not supply the same molar concentration of iron. 20 µM FeCl_3_ was used as the benchmark as this was slightly higher than that required for optimal growth for *E. coli* [94], but was considered unlikely to be high enough to allow the bacteria to build up iron reserves and subsequently alter their transcriptional profile in response to using internal iron.

### Description of RPMI-based medium agar

The basal medium contained 660 ml 1× RPMI Medium 1640 with L-glutamine (Invitrogen), 10 g BBL agar Grade A (BD biosciences), and 10 ml supplementary 400 g/l glucose (to achieve a final concentration of 33 mM) made up to 1 litre with HPLC quality water. All media was prepared in glassware that had been thoroughly washed in HPLC quality water.

### RNA extraction

Cultures were grown on RBM medium, with supplements, for 16 hours at 37°C, with 5% (v/v) CO_2_, before RNA extraction. RNA extraction was performed using the TriZol/RNeasy method. Bacteria were harvested from the plate into 500 µl of RNA*later*™ (Ambion Inc.) and homogenized by vortexing. The bacteria were pelleted at 2,250 g for 15 minutes, resuspended in 1 ml of Trizol (Invitrogen), and vortexed for 10 minutes to ensure complete cell lysis. 300 µl of chloroform was added and incubated at room temperature for 2 minutes. Cells were pelleted at 13,000 g for 15 minutes at 4°C. The aqueous phase was added to an equal measure of isopropanol and incubated at room temperature for 10 minutes. The samples were then spun at 13,000 g for 15 minutes at 4°C to pellet the RNA. The pellet was resuspended in 100 µl of nuclease free water. RNA was then purified using the RNeasy® kit (Qiagen) according to the manufacturer’s instructions, except the RW1 buffer wash step was not performed. The RNA was finally eluted into 30 µl of nuclease free water to which 1 µl of SUPERase-In™ (Ambion Inc.) was added. RNA quantity and contamination was measured by absorbance at 260 nm using a NanoDrop spectrophotometer (LabTech International Ltd). RNA integrity was confirmed with the RNA 6000 assay using a 2100 Bioanalyzer (Agilent Technologies UK Ltd).

### Pan-Neisseria microarray v.2

The expanded pan-*Neisseria* microarray v.2 (PNA-2) has been described previously [95]. In summary, this is a PCR-product based array printed with 3 non-adjacent replicates, which includes probes for all of the annotated regions in the published genome sequences, plus some additional neisserial and experimental-tool genes. The microarrays were printed with a Genetix QArray Mini microarray printer using 150 µm solid aQu pins onto Genetix Amine slides. The array is described more fully on the Saunders group web page via www.path.ox.ac.uk and is available on an academic collaborative basis. A complete Array Definition File is available from ArrayExpress accession number: A-MEXP-219. Interpretation is based primarily upon the annotations from TIGR with additional corrections for genes for which subsequent functions have been defined, based upon literature searches of potentially significantly changed genes. A re-annotation of this genome is currently in progress (by NJS), and the revised annotation will be backwardly compatible with this data, and will be added to the existing GBrowse databases supporting this study.

### Microarray hybridization

2–3 µg of total RNA, from bacteria grown on two different iron sources, was reverse transcribed and labelled using the 3DNA Array 900MPX kit (Genisphere) with the following modifications: Reverse transcription was performed with SuperScript III (Invitrogen). Purification of cDNA and purification of tagged cDNA was performed using the DNA clean and concentrate-5 kit (Zymoclean) according to the manufacturer’s instructions. The tagged cDNAs were pooled and pipetted under a 22×50 LifterSlip (Erie Scientific) on a prehybridized PNA-2 (3.5× SSC, 0.1% SDS and 10 mg/ml bovine serum albumin for 20 minutes at 65°C). Arrays were pre-warmed on a SlideBooster (Advalytix) and the first hybridization was at 60°C with mixing for 16 hours. Slides were washed in 2×SSC, 0.2% SDS at 60°C for 10 minutes and then 2×SSC and 0.2×SSC at room temperature for 10 minutes each. Slides were dried using an airbrush. The second hybridization was performed in the same way at 55°C for 4 hours. Washing was the same except the initial wash was at 55°C. Biological replicates were labelled in a dye-balanced fashion to exclude dye labelling biases. The slides were scanned with a ScanArray Express HT microarray scanner (Perkin-Elmer) at 5 µm pixel resolution, using automated calibration to 98% saturation for the most intense features. Six pair-wise biological replicate arrays were performed for each comparison, except the Hb vs.ferric chloride and Hb vs. Hb&Tf comparisons that used seven biological replicates.

### Microarray data analysis

Image analysis, data extraction, and manual flagging to exclude local artefacts and non-reporting features, was performed using BlueFuse v.3.0 (BlueGnome), with replicate spot data being combined using the fusion algorithm.

The MIAME compliant experiment description, raw extracted image data, as well as the flagged fused files, are available from ArrayExpress (http://www.ebi.ac.uk/arrayexpress/) as experiment: E-MEXP-1143.

The spot intensity data was analyzed within BASE [96]. Normalization of the data was performed using a global median normalization excluding features with intensity in either channel lower than 200 and the 10% most intense and least intense reporters. The data reported for each gene represents the median fold ratio obtained from at least three slides. The Cyber-T test [97] was used to determine the significance of the fold ratio change. The Cyber-T test considers the rank order of genes in each biological replicate and uses a Bayesian probabilistic approach to determine the significance of changes based upon the consistency of this rank order between replicates. This approach is less susceptible to dynamic range differences and the type 1 and type 2 errors associated with t-tests with this type of data. Transcripts that were reliably detected on at least 3 arrays that differed by a fold-ratio of >1.5 were considered to be potentially biologically important, and of these, those with a *p*-value of <0.05 in the CyberT test (with the parameters in Table 4) were considered to be significant. The ‘consistency’ values (shown in the result tables) indicate the number of replicates in which a similar direction of change was seen for each transcript, in the replicates in which the transcript was measured. The single channel data (using a 3% cross-channel intensity correction of unnormalized data, and the 51^st^ to 551^st^ most abundant genes, scaled to a common factor) exported from BASE was analyzed for selected genes using Microsoft Excel. The BASE plug-ins for the normalization, Cyber-T, single channel data export, and consistency analyses, are available to other BASE users from the authors on request.

**Table 4:** CyberT values for analysis of individual comparisons.

Comparison	Sliding window size	Bayes confidence estimate
Hb *vs.* Tf	77	12
Hb *vs.* Lf	77	15
Tf *vs.* Lf	91	15
Hb *vs.* FeCl_3_	71	12
Hb *vs,* Hb&Tf	101	12

### Real-time quantitative PCR for confirmation of pan-*Neisseria* microarray v.2 data

Quantitative real time PCR (qRT-PCR) was used to verify the changes in a representative sample of key genes showing increased, reduced, or similar expression between the different iron donor conditions tested (listed for each comparison in Dataset S2). qRT-PCR primers (Table S2.4) were tested for specificity in PCR assays using genomic templates. Using the same RNA prepared for the expression profiling, 5 µg of total RNA from three biological replicates was further purified from genomic DNA contamination using DNA-*free* (Ambion). The RNA was then reverse transcribed using SuperScript III using the same reagents as were used for the samples assessed by microarray. After reverse transcription cDNA was purified using Qiaquick PCR purification kit (Qiagen), according to manufacturer’s instructions, and equivalent concentrations of the three different cDNA samples were pooled, due to the lack of sample cDNA. 50 ng of cDNA and 200 nM of primer were used in qRT-PCR using a Rotor-gene 3000 (Corbett Life Science; Cambridge, Cambridgeshire) and SYBR Green master mix, according to manufacturer specifications (Stratagene).

The relative transcript level of each gene was normalized to *nmb0956* (*sucB*), on the basis of the microarray data which showed it to be invariant between all conditions tested. Quantitative values were obtained by using the comparative threshold cycle (ΔΔ*C_T_*) method, as described in Livak *et al.* [98]. Each gene was assayed in triplicate from the pooled cDNA, and the mean *C_T_* value was used for further comparison. Non-template controls were run with each set of reactions to identify any primer dimer artefacts. The results confirmed the microarray data for all genes tested, and are shown in Dataset S2.

### Comparison of the transcriptional profile of *N. meningitidis* strain MC58 in the presence and absence of iron

Transcriptional profiles in the presence and absence of iron were determined similarly, and are described fully in Dataset S1.

### Serum Killing Assay

Serum bactericidal activity was determined in normal human serum (NHS) isolated from one donor, although similar results were obtained with NHS from another donor. RBM agar cultures 4 hours old were used to prepare bacterial suspensions for each iron source at an OD_600_ of 0.3 (approximately 2×10^6^ CFU/ml) in RPMI. Assays were performed with 100 µl of bacterial suspension, 90 µl PBS (Oxoid) (containing 0.9 mM MgCl_2_ and 0.7 mM CaCl_2_), and 10 µl NHS or heat-inactivated (HI) NHS (56°C for 30 min) to a final serum concentration of 5%. To ensure the bacteria were not clumping bacterial suspension for each iron source was added to RPMI without serum and the number of CFU counted. The CFU were similar for each showing that clumping was not occurring. After incubation for 60 min at 37°C with 5% CO_2_, serial dilutions were made for quantitative culture on GC media. To calculate the percentage of survival, colony counts in triplicate were compared to HI-NHS controls. Data are expressed as percent survival in NHS compared to that in HI-NHS [(CFU in NHS/CFU in HI-NHS)×100]. Statistics were performed using an unpaired 2-tailed t-test.

## Supporting Information

Dataset S1 (1.83 MB PDF)

Dataset S2 (0.03 MB PDF)

Table S1 Genes up-regulated in the presence of Haemoglobin compared to Transferrin. ^1^ Fold ratio is the relative transcript abundance in the presence of Haemoglobin compared to the presence of Transferrin. ^2^ The number of comparisons in which this gene was reliably detected. ^3^ A measure of the number of comparisons in which the gene was changed in the same direction. a-all one direction, b-one in opposite direction, c-two in opposite direction.
(0.03 MB PDF)

Table S2 Genes up-regulated in the presence of Transferrin compared to Haemoglobin. ^1^ Fold ratio is the relative transcript abundance in the presence of Transferrin compared to the presence of Haemoglobin. ^2^ The number of comparisons in which this gene was reliably detected. ^3^ A measure of the number of comparisons in which the gene was changed in the same direction. a-all one direction, b-one in opposite direction, c-two in opposite direction.
(0.02 MB PDF)

Table S3 Genes up-regulated in the presence of Haemoglobin compared to Lactoferrin. ^1^ Fold ratio is the relative transcript abundance in the presence of Haemoglobin compared to the presence of Lactoferrin. ^2^ The number of comparisons in which this gene was reliably detected. ^3^ A measure of the number of comparisons in which the gene was changed in the same direction. a-all one direction, b-one in opposite direction, c-two in opposite direction.
(0.02 MB PDF)

Table S4 Genes up-regulated in the presence of Lactoferrin compared to Haemoglobin. ^1^ Fold ratio is the relative transcript abundance in the presence of Lactoferrin compared to the presence of Haemoglobin. ^2^ The number of comparisons in which this gene was reliably detected. ^3^ A measure of the number of comparisons in which the gene was changed in the same direction. a-all one direction, b-one in opposite direction, c-two in opposite direction.
(0.03 MB PDF)

Table S5 Genes up-regulated in the presence of Transferrin compared to Lactoferrin. ^1^ Fold ratio is the relative transcript abundance in the presence of Transferrin compared to the presence of Lactoferrin. ^2^ The number of comparisons in which this gene was reliably detected. ^3^ A measure of the number of comparisons in which the gene was changed in the same direction. a-all one direction, b-one in opposite direction, c-two in opposite direction.
(0.02 MB PDF)

Table S6 Genes up-regulated in the presence of Lactoferrin compared to Transferrin. ^1^ Fold ratio is the relative transcript abundance in the presence of Lactoferrin compared to the presence of Transferrin. ^2^ The number of comparisons in which this gene was reliably detected. ^3^ A measure of the number of comparisons in which the gene was changed in the same direction. a-all one direction, b-one in opposite direction, c-two in opposite direction.
(0.01 MB PDF)

Table S7 Genes up-regulated in the presence of Haemoglobin compared to Ferric chloride. ^1^ Fold ratio is the relative transcript abundance in the presence of Haemoglobin compared to the presence of Ferric chloride. ^2^ The number of comparisons in which this gene was reliably detected. ^3^ A measure of the number of comparisons in which the gene was changed in the same direction. a-all one direction, b-one in opposite direction.
(0.02 MB PDF)

Table S8 Genes up-regulated in the presence of Ferric chloride compared to Haemoglobin. ^1^ Fold ratio is the relative transcript abundance in the presence of Ferric chloride compared to the presence of Haemoglobin. ^2^ The number of comparisons in which this gene was reliably detected. ^3^ A measure of the number of comparisons in which the gene was changed in the same direction. a-all one direction, b-one in opposite direction, c-two in opposite direction.
(0.02 MB PDF)

Table S9 Genes up-regulated in the presence of Haemoglobin compared to Haemoglobin and Transferrin. ^1^ Fold ratio is the relative transcript abundance in the presence of Haemoglobin compared to the presence of Haemoglobin and Transferrin. ^2^ Fold ratio is the relative transcript abundance in the presence of Haemoglobin compared to the presence of Transferrin. 3 The number of comparisons in which this gene was reliably detected. 4 A measure of the number of comparisons in which the gene was changed in the same direction. a-all one direction, b-one in opposite direction, c-two in opposite direction.
(0.02 MB PDF)

Table S10 Genes up-regulated in the presence of Haemoglobin and Transferrin compared to Haemoglobin. ^1^ Fold ratio is the relative transcript abundance in the presence of Haemoglobin and Transferrin compared to the presence of Haemoglobin. ^2^ Fold ratio is the relative transcript abundance in the presence of Transferrin compared to the presence of Haemoglobin. 3 The number of comparisons in which this gene was reliably detected. 4 A measure of the number of comparisons in which the gene was changed in the same direction. a-all one direction, b-one in opposite direction, c-two in opposite direction.
(0.03 MB PDF)

Table S11 Genes up-regulated by Haemoglobin
(0.02 MB PDF)

Table S12 Genes down-regulated by Haemoglobin
(0.02 MB PDF)

Table S13 Genes up-regulated by Transferrin
(0.01 MB PDF)

Table S14 Genes down-regulated by Transferrin
(0.01 MB PDF)

Table S15 Genes up-regulated by Lactoferrin
(0.01 MB PDF)

Table S16 Genes up-regulated by Lactoferrin
(0.01 MB PDF)
