# Supplementary material for: Host Iron Binding Proteins Acting as Niche Indicators for Neisseria meningitidis
Source: PLoS One. 2009 Apr 8;4(4):e5198. doi: 10.1371/journal.pone.0005198 (PMC2662411; doi:10.1371/journal.pone.0005198)
Supplement: Dataset S1 — (1.83 MB PDF) [file pone.0005198.s001.pdf]

Supplementary Dataset 1

The effect of the presence and absence of iron on the transcriptional profile of *N. meningitidis* strain MC58

Summary

In order to distinguish the responses to iron obtained from specific iron donors from the cellular responses to iron availability in *N. meningitidis* strain MC58, experiments comparing the effects of the presence or absence of iron on gene expression were performed. The response to iron is also of interest because its restriction is often correlated with increased virulence in many bacteria. This study uses different tools and media conditions to previous studies addressing this response, and has been compared with two earlier studies, addressing the transcriptional response of *Neisseria* spp. to the absence of iron, performed on *N. meningitidis* strain MC58 and *N. gonorrhoeae* strain FA1090.

The expression of 192 genes are significantly and reproducibly different between the two conditions. In the absence of iron genes associated with iron-uptake, capsule expression, and virulence are induced. In the presence of iron, transcripts encoding iron-containing proteins of the electron transport chain and iron-storage proteins are increased. Several transcriptional regulators are differentially changed in response to iron availability and these may be responsible for the changes in expression of the iron-responsive genes that are not controlled by Fur.

Comparisons with two previous studies show striking differences between the three sets of results. The differences between species are large with almost no similarity in the reported transcriptional responses. However, the comparison between the two studies of strain MC58 identifies 50 genes that are common to both studies, whilst there are considerably more genes identified that are unique to each. This may indicate that the core response to iron is smaller than previously proposed and that the inconsistent responses are dependent on other environmental factors. We believe the most important difference between the two studies of strain MC58 in this regard is that this study measures the adapted

response of this strain to the absence of iron, while the previous study addressed the transcriptional response during the process of adaptation.

Introduction

This is the third study to describe effects of the presence or absence of iron on gene transcription in the pathogenic *Neisseria* spp., two previous studies have been reported using: *N. meningitidis* strain MC58 [1] and *N. gonorrhoeae* strain FA1090 [2]. Even though this study used the same strain as Grifantini *et al.* [1] it was necessary and useful to repeat the investigation of the response to the presence or absence of iron because:

1. The microarray and hybridization methods must be consistent with the study of responses to specific iron donors to facilitate the comparative analysis of this data.
2. The labelling method used in these experiments is more sensitive and quantitative than those used in the previous study, and has to be similar to permit proper comparisons.
3. The culture conditions differ, such that the iron was not similarly depleted from the starting cultures, the media used was more ‘nutrient rich’, and a different sampling time-point was used.
4. The analysis used in the other experiments cannot be reproduced sufficiently to draw proper comparisons with the experiments addressing specific iron sources. For example, which Cyber-T algorithm and the parameters used in the analysis (window size and degrees of freedom) are not stated, and the raw image data is not available to allow for a similar image extraction and analysis to be conducted.
5. The previous experiments were performed with fewer biological replicates than are needed to determine consistent biological responses.

These differences will be discussed further when the results of the two studies of *N. meningitidis* strain MC58 are compared.

Results

Eleven biological replicates were performed comparing growth in the presence and absence of iron. Two of these were excluded due to poor data depth (< 500 reported probes). Two slides were excluded because changes in the transcription of the *bfrAB* and *tbpB* genes, which are established markers for the

presence and absence of iron respectively, were not seen. Therefore, the final set of seven comparisons included RNA from cultures grown on four separate days from a common stock on independently prepared media to minimize the influence of responses to culture conditions and handling on the final data. The experiment was performed with a dye-balanced design, with four replicates labelled in one orientation, and the remaining three in the other. Genes that were reliably detected on at least three replicates, and with a fold-ratio of > 1.5 were considered to be potentially biologically important, and those with a *p*-value of < 0.05 in the CyberT test (using a sliding window size of 101, and a Bayes confidence estimate value of 9) were considered to be significant. Consistency data for each gene, as described in the accompanying paper, is shown in the results tables. With these parameters there are 136 transcripts are more abundant in the absence of iron (Table S1.1) and 56 in the presence of iron (Table S1.2).

The effects of the absence of iron on the meningococcal transcriptional profile

Transcripts with increased abundance in the absence of iron are shown in Table S1.1. 59 of these genes have annotated functions, and these are predominantly associated with iron transport, the outer membrane, and energy metabolism.

Functions directly related to iron monitoring, acquisition, and recognized neisserial iron-specific responses

The most studied regulator controlling responses to a lack of iron is the ferric uptake regulator; Fur. In this study, expression of *fur* is increased only 1.4 fold ($p = 0.06$) in the absence of iron. When bound to iron, it predominantly acts as a transcriptional repressor, but as iron is depleted the structure of Fur is lost and in most cases it no longer binds the target DNA [3-5]. Fur can also act as an activator, either directly or indirectly, as seen in the Fur knockout mutants of *N. meningitidis* [6] and *E. coli* [7]. Fur is constitutively present in very high copy numbers within the cell in *E. coli* [8,9], so it is not primarily the levels of Fur transcript and protein that report its function, but the number of Fur proteins that are bound to iron that is important in controlling gene transcription levels. However, the reduction of autorepression reflects this altered activity.

Genes encoding iron uptake systems for transferrin (*tbpA* and *tbpB*), lactoferrin (*lbpB*), and siderophores (*frpB*) were induced. The change in *lbpB* expression did not quite meet the inclusion criteria (fold ratio = 1.5 fold, $p = 0.055$). Hybridization to the probe is seen in three slides, two of which show an increase in expression in the absence of iron and one in which its expression is equivalent. This anomalous data point has intensity values just above background levels, while the intensities are greater in the other two comparisons, suggesting this data point may be inaccurate. These genes have all been previously shown to be responsive to iron levels [10-12]. They are all functionally dependent upon the TonB/ExbB/ExbD system to supply energy for iron transport [13-15], and *exbB* and *exbD* are also induced in the absence of iron. The majority of characterized TonB-dependent receptors are involved in iron uptake, and the increase in gene expression of another putative TonB-dependent receptor (NMB0293) may also be functionally linked. Notably, the expression of the haemoglobin receptor, *hmbR*, was not induced, even though it has been described previously to be regulated in response to iron availability in *N. meningitidis* [16]. However, consistent with our data *hmbR* was not reported in the previous microarray-based iron restriction studies in either species [1,2].

The expression of seven iron responsive proteins (Frp) and four associated genes, located in seven genomic locations, have been described previously to be associated with iron restriction [17-20], (NMB0364-NMB0365; NMB0584- NMB0585; NMB1403-NMB1405; NMB1407; NMB1409; NMB1412; and NMB1414- NMB1415), and, apart from NMB1403, NMB1405, NMB1407, NMB1409 and NMB1415, these are also consistently induced in this study. The *frpA* and *frpC* genes encode homologues of RTX proteins that contain a repeat, of unknown function in *Neisseria* spp., but has been shown to be present in exotoxins in many other Gram-negative species, including HlyA in *E. coli* and RtxA in *Vibrio* spp. [21,22].

Non-Fur regulators involved in the response to the lack of iron

There are many roles for iron within the cell, being involved in the structure and function of other regulators, such as FNR, as well as being integral to components of electron transport chains, providing resistance to oxidative stress, and other general cellular functions. A physiological lack of iron is therefore likely to have a range of direct and indirect effects that are not limited to the acquisition of iron itself. Consistent with this, Grifantini *et al.* predicted that 50% of the genes they found to be regulated by iron restriction were not directly regulated by Fur [1].

Up-regulation of *mdaB* (NMB1857), a gene probably involved in resistance to intracellular stress from reduced quinone levels within the cell, is associated with the presence of the *crgA* gene [23]. *crgA* encodes the contact-regulated gene, thought to be involved in regulation of itself and *mdaB*. *crgA* is never detected in the microarray data (Saunders, unpublished observations), possibly because its mRNA is present at very low copy numbers within the cell. Transcript levels were measured by quantitative real-time PCR (qRT-PCR), which showed increased transcript (8.6-fold) in the absence of iron, consistent with the observed *mdaB* changes. Wild-type MC58 expresses Fur and the iron-uptake genes *tbpAB* and *lbpAB*, at higher levels than those seen in a *crgA* deletion mutant (Saunders, unpublished data) suggesting that it may be involved in the response to iron. *crgA* may be responding to intracellular redox levels through monitoring the abundance a redox protein (e.g. quinone) in order to control expression of *mdaB* and iron-responsive genes because the redox state will be directly related to the ability to use iron-containing electron transport chains.

Annotated regulators that are increased in the absence of iron in this study include: Hfq (NMB0748), which has been found to be important in *E. coli* responses to iron through interactions with the non-coding RNA *ryhB*, which is repressed by Fur in the presence of iron [24,25]; a putative cold-shock regulator (NMB0838); ribonuclease inhibitor barstar (NMB0646); and the DNA binding protein HU-β (NMB1230). However, the regulatory effects of these genes have yet to be characterized in *Neisseria* spp., or in the iron responses of other species.

Metabolic responses

The absence of iron is associated with changes in metabolism with an increase in transcription of genes associated with non-oxidative phosphorylation. There is increased production of L-lactate and D-lactate through up-regulation of *lld* and *gloA* respectively. It is likely there is increased fermentation as the mRNA levels of an alcohol dehydrogenase (NMB1395) and the putative carboxyphosphonoenolpyruvate phosphonomutase increase. Therefore the absence of iron is associated with a physiological response consistent with an inability to use oxygen as a terminal electron acceptor.

Cell surface structures and potential virulence-associated gene responses

There are large changes in the transcription of genes encoding the capsule and proteins associated with the external cellular surface, indicating that significant remodelling of the meningococcal cell surface

occurs. The capsule is the major virulence determinant of *N. meningitidis* and is important in preventing desiccation during transfer between hosts and in prevention of killing by human serum, either through complement attachment, phagocytosis or insertion of the membrane attack complex [26,27]. Expression of *porA*, one of the major meningococcal surface proteins, is also increased.

Virulence factors induced in the absence of iron include a haemolysin (NMB1646), and a putative toxin-activating protein (NMB1210), required for converting excreted toxins into their active form. Up-regulation of the IgA specific endopeptidase is observed, which is involved in the cleavage of IgA molecules [28] and LAMP-1 [29,30] thereby preventing their effector function. Increased expression of this gene is also seen in invasive isolates of *N. meningitidis* [31]. Other virulence factors observed include NMB1994, a putative adhesin, which is regulated in a deletion mutant of PhoPQ [32], although *phoPQ* expression is not reported in this study. Expression of *sodC*, a superoxide dismutase, is also increased, as distinguished from the *sodB* transcript, which is increased in the presence of iron. SodC has not been shown to be important for resistance to internal oxidative stress like SodB, but is important for resistance to external oxidative stress and in preventing phagocytosis by macrophages and monocytes [33,34].

Effects of the presence of iron on the meningococcal transcriptional profile

In the presence of iron the consistently increased transcripts include those encoding the bacterioferritin proteins involved in iron-storage. This confirms that under these conditions iron is no longer a limiting resource. Of the 56 genes increased there are 21 of currently unknown function. The remainder mostly encode genes pertaining to energy metabolism and protein synthesis genes (Table S1.2).

Cellular metabolic responses

There is increased transcription of genes involved in protein synthesis in the presence of iron, particularly the genes involved in synthesis of ribosomal RNA as well as for several proteins that are associated with the ribosomes (NMB0876, NMB1321, NMB1323, and NMB2057), although not the ribosomal protein operon. As well as an increase in translation machinery there is up-regulation of genes involved in protein folding, including: *clpP*, *dsbC* and *groEL*.

Electron transport chains are dependent on the presence of iron because the redox cores of these proteins contain iron. In *E. coli* the transcription of genes encoding proteins with iron cores are up-regulated in the presence of iron [24]. Similarly, transcripts encoding components of electron transport chains (complexes I, II, and IV) are increased in the presence of iron in *N. meningitidis*. These include *nuoA* of the NADH dehydrogenase complex and *fixN* from the cytochrome C oxidase complex, which have previously been associated with the Fur-dependent response in *N. meningitidis* [6]. Related changes in sugar metabolism are also observed. Transcripts encoding subunits of the succinate dehydrogenase complex (*sdhABD*) are increased and have been shown to be regulated by the small iron-responsive RNA NrrF [35]. The activity of these proteins determines the activity of the TCA cycle and complex II of the electron transport chain. Also, *pdhA*, a component of the pyruvate dehydrogenase complex, is increased, which catalyzes the formation of acetyl-CoA from pyruvate at the entry point of glycolysis into the TCA cycle.

Another reflection of increased metabolic activity is increased expression of genes required for biosynthesis of the branched chain amino acids, leucine, and isoleucine. *N. meningitidis* uses leucine as an indicator of the metabolic state of the cell [36], and a proportion of the phenotype seen in the presence of iron may be mediated through this.

Transcriptional regulators

Only one transcriptional regulator NMB0398, an ArsR-family regulator, was found to have greater expression under iron-rich conditions. This regulator has yet to be functionally investigated in this species.

Quantitative PCR to confirm microarray expression data

qRT-PCR was used to validate the microarray data, addressing representative functionally relevant transcripts that are increased in iron-deplete conditions (*ilvD-1* and *bfrB*), iron-supplemented conditions (*fur*, *tbpB*, *iga*, NMB1857), or for which expression was unchanged (*sucB*). The qRT-PCR results were similar to those obtained from the microarrays (Table 3), indicating that the microarray results give an accurate report of transcript levels within the bacterium.

Regulation of transcriptional units and the location of putative Fur sites

Genes were assigned to transcriptional units based upon the predicted direction of transcription of surrounding genes, the proximity to nearby genes (normally < 100 bp), the position of rho-independent transcriptional terminators, and a separate analysis of the co-regulation of genes in a comparison of the transcriptional profiles of *N. meningitidis* strain MC58 comparing growth on GC and RBM agar (Jordan, Capper & Saunders, unpublished). The iron-regulated putative transcriptional units are shown in Table 4, which also indicates whether these genes are associated with putative Fur boxes in the region of -400 to +200 bp of the first initiation codon. Identification of the Fur box used the $(NATWAT)_3$ consensus sequence of Escolar *et al.* [37], allowing up to three mismatches. This concensus sequence has been shown to predict Fur binding in *Neisseria* by Grifantini *et al.* using EMSA studies [1]. Due to degeneracy in the concensus sequence the search allowing three mismatches requires at least 9 of 12 of the most highly conserved AT residues to be present, compared to the less stringent search used by Grifantini *et al.* [1], which allowed up to six mismatches to the consensus. The lower stringency used in the previous study identified Fur binding sites in front of 31% of all the group B meningococcal genes, which is inconsistent with the scale of both their and our iron depletion responses, and the data from a knockout mutant of Fur [1,6]. The more stringent criteria used in this study identifies Fur sites in the 5' regions of only 13.5% of all genes, which includes 73 of the iron-regulated genes in this study, representing 38% of all iron-regulated genes. In total 42 of 114 putative transcriptional units that contain genes up-regulated in the absence of iron have putative Fur sites, while seven of 50 putative transcriptional units with greater expression in the presence of iron have putative Fur sites. This is consistent with the concept that Fur is predominantly a repressor. The lack of Fur sites in the putative promoter regions for 119 genes reflects that the response to the lack of iron is complex and involves additional regulators.

Comparison of the iron depletion response with that previously described by Grifantini *et al.*

This study differs in several ways from the work by Grifantini *et al.*, which also compared the transcriptional profiles of *N. meningitidis* strain MC58 in the presence and absence of iron [1]. The primary reason for repeating this study was to provide a robust dataset for comparison with the

responses to specific iron sources. However, it also provides an opportunity to corroborate, and also to explore any substantive differences with the previous study.

Potentially important differences between the experimental conditions used in this study that may account for some of the differences observed include:

1. That the bacteria were grown on solid media, whereas the previous study used liquid media.
2. That this study includes a greater number of biological replicates (7 vs. 3).
3. That this study looked at the growth after 16 hours while Grifantini *et al.* compared the growth of *N. meningitidis* at several time points during the first 5 hours, which although comparatively short will also include changes associated with growth-phase.
4. That this study observed the transcriptional profile of bacteria at a time-point associated with adapted growth, rather than during the process of adaptation.
5. That this study used a culture on a defined medium that inherently lacks iron in order to address the specific response to iron restriction. In contrast, Grifantini *et al.* included the iron chelator desferal, which may also bind copper, nickel, and zinc.

The first and second points are important because we have consistently found that expression profiles from liquid batch cultures are less reproducible between biological replicates than growth on solid media (Capper & Saunders, unpublished), and the use of only three biological replicates with liquid media does not optimally control for culture-to-culture behavioural differences. The influences of the third and fourth points above are illustrated by the way that transcripts showed increases and decreases at different time points in the previous study. Differences in the transcriptional profile during adaptation to the absence of iron rather than the adapted response to the absence of iron have also been shown in *C. jejuni* [38].

Genes that are common components of the responses to iron in these two studies

The genes that are present in the results of both studies and are up-regulated in the presence or absence of iron are shown in Tables 5 and 6 respectively, and the numbers of differences are illustrated in Figure 1.

There is only limited similarity between the genes responsive to iron in these two studies, with only 50 of the 192 genes in this study, and 234 genes in the Grifantini *et al.* study, observed to be significantly

regulated in the response to iron in both studies. These are stronger candidates to be part of a core iron restriction response.

Only eight genes are found to be up-regulated in both studies in the presence of iron. These are the genes encoding the ArsR family transcriptional regulator (NMB0398), bacterioferritin A and B, *sodB* and several conserved hypothetical proteins, including NMB1437 and NMB1438, identified by Grifantini *et al.* as being important for survival in response to reactive oxygen species [39]. NMB1436, which also forms part of this transcriptional unit, showed detectable increased expression in the presence of iron in only two of the seven slides.

Forty-two genes are increased in both studies in the absence of iron. This includes genes involved in iron uptake (*tbpA*, *tbpB*, *lbpB*, *frpB*, *exbD*), and the iron-responsive *frpA* and *frpC* transcriptional unit genes (NMB0364, NMB0365, NMB0584, NMB1412, NMB1414). There is increased transcription of two *mdaB* homologues (NMB0977, NMB1857) and a homologue in *Helicobacter pylori* may be involved in host colonization and resistance to oxidative stress [40]. Transcription of NMB1857 is associated with *crgA* expression [23], which is up-regulated in this study and may be an important regulator of the iron response.

Inconsistencies between the two *N. meningitidis* strain MC58 studies

It is important to note that the Grifantini *et al.* study addressed the responses, in a comparatively early time series, to the addition of iron (in which depletion of other ions was not corrected) rather than comparing stable expression in the presence or absence of iron. Grifantini *et al.* identified a large number of differentially regulated genes at the 30 minute time point that were not found to be changed in this study. These include induction of the functionally inactive *rpoN* transcript, presumably indicative of cellular stress. At this specific time point 49 genes were identified that were not changed in their study at other time-points. Therefore, a component of these ‘early iron response’ genes may actually be in response to an additional source of stress following the addition of iron, rather than a specific adaptation to the presence of iron.

Grifantini *et al.* observed increases in *fnr* and *aniA* transcripts, which are involved in anaerobiosis [41-43], in the absence and presence of iron respectively, but these transcripts were not detected in this study. FNR, which activates *aniA* [44], has an iron-sulphur core and the loss of *fnr* auto-repression may

be as a result of the loss of this core. In the microaerobic conditions of liquid culture the observed *fnr* response may reflect a ‘false signal’, due to the loss of this iron-sulphur core, that are not a feature of growth on aerobic solid media. *fbpA*, *secY*, *recN*, and *fur* are amongst the genes previously reported to be iron-regulated and found to be altered by Grifantini *et al.*, but are unchanged in this study.

Seventeen genes are down regulated in the presence of iron in this study but up-regulated in the study by Grifantini *et al.*, the majority of which are involved in protein synthesis, transcription or energy metabolism. Nine of these genes were only previously changed at one time-point, several at the same time point as the *rpoN* transcript, suggesting these may have a different role during adaptation than in the stable presence or absence of iron.

Only 50 of the 327 (15%) differentially transcribed genes were common to the two studies of *N. meningitidis* MC58. These potentially constitute a core response to iron restriction. This highlights the critical importance of the experimental conditions in determining transcriptional responses. The different media, solid vs. liquid growth, and the time at which the profiles were studied, as well as RNA extraction, microarray manufacture, microarray analysis, will all have potentially contributed to these differences. The differences in growth phase and media may explain the high numbers of protein synthesis and metabolism genes that were different in the results of the two studies.

Comparison of the iron depletion response with that previously described by Ducey *et al.*

The data can also be compared to a study by Ducey *et al.* on the effect of iron limitation in *N. gonorrhoeae* [2]. In their study, the effect of iron limitation was studied by growing iron depleted *N. gonorrhoeae* in chemically defined medium broth with or without 10 μM ferric nitrate and extracting the RNA at mid-log phase. This experiment was performed in liquid media, in which expression is less reproducible than on solid media, as already described, and includes a degree of oxygen limitation. Some differences between the findings of the two comparisons may be as a result of this. However, a comparison between our data and this study identifies large differences in the response of the two neisserial species to iron.

Based upon these two studies the responses to the presence and absence of iron have little in common between these two closely related species, which is consistent with the conclusion made by Ducey *et al.* following their comparison to the data of Grifantini *et al.*. Only 13 (6.4%) of the 203 genes identified by Ducey *et al.* show changes in the same direction in the presence or absence of iron in this study. This increases to 12.5% when the gonococcal specific transcripts and transcripts for which there is no data in this study are excluded. However, as the two organisms are so closely related a more comparable response might have been expected. When Ducey *et al.* compared their data to that of Grifantini *et al.*, they found that only 16.4% of their genes were differentially regulated in the same direction, which is similarly low, although these are more comparable studies because they are both in liquid and both are at mid-log phase at one point.

The data showing the genes that are up-regulated in the presence and absence of iron in both studies are shown in Tables 7 and 8, and the numbers of genes that are differently regulated between the two species are shown in Figure 2.

Only two conserved hypothetical genes (NMB0117 and NMB2140) were more highly expressed in the presence of iron in both studies. The common response to low iron involves genes in iron uptake (*tbpB*, *tbpA*, *frpB* and *exbB*) and the DNA binding protein HU-β. There are also six hypothetical genes including NMB0744, which has weak homology to a haem binding protein.

The two pathogenic *Neisseria* spp. are generally similar in terms of gene content, and so these differences in the response to iron were unexpected. *N. gonorrhoeae* may have a fundamentally different physiology such that iron availability may not act as a signal in the same way as in *N. meningitidis.* Alternatively the differences might be due to the iron concentrations used in the different experiments. This study and that of Grifantini *et al.* used 100 μM iron in, while Ducey *et al.* used 10 μM iron and 10 μM iron may still be a relatively iron-restricted concentration. We found *N. gonorrhoeae* strains required between 2 and 5 μM iron for growth on RBM. and possibly what is observed is an intermediate transcriptional response used when iron is neither limiting or excessive. Notably, Ducey *et al.* did not see changes in the bacterioferritin genes in the presence of iron was still limiting.

In addition Ducey *et al.*'s two cultures were shown to have very different growth rates at the mid-log phase point used for their comparison as many of the genes seen to be changed in their study were

associated with changes in transcription and the ribosomal proteins, which is consistent with the substantial influence of differing growth rates rather than the response to iron.

Conclusions

This study provides further evidence that the meningococcal response to iron limitation has parallels with that of other bacteria, in which the absence of iron causes up-regulation of genes encoding iron acquisition and virulence proteins and the down-regulation of genes encoding iron-containing metabolic proteins. The stable absence of iron is not associated with a significant change in the levels of Fur transcript, although several Fur-controlled genes are regulated indicating its altered activity. Putative Fur binding sites were identified upstream of 38% of the genes regulated in this response, which is more consistent with the results of a Fur knockout study [2], than those of Grifantini *et al.*, which identified binding sites upstream of 50% of their iron response [1]. This suggests that the response to iron restriction involves other regulators; and this study identifies a candidates including: Hfq, HU-β, NMB0838 and NMB0398.

This is the third comparison of the neisserial response to iron restriction. 26% of the genes regulated in this study are similarly regulated in the previous study of the effects of iron restriction on MC58 [1]. However, the previous comparison measured the adaptive response to the addition of iron rather the adaptive response of growth in the absence of iron, which may underlie the substantial differences between these two studies. Neither this study nor that of Grifantini *et al.* shows similarity to the results of Ducey *et al.* studying iron restriction in *N. gonorrhoeae* [2], possibly because of the low iron concentrations used in the gonococcal study, or because there are fundamental differences between the physiological response to iron in these species.

Materials and Methods

N. meningitidis strain MC58 *siaD*$^-$ [45] was passaged from the -80°C freezer on GC agar with the Kellogg supplement and ferric nitrate [46] at 37°C with 5% (v/v) CO_2. 10-20 colonies were then transferred to RBM agar (as described in accompanying paper) either supplemented with 100 μM $FeCl_3$ or with no iron and incubated overnight.

The microarray, RNA extraction, hybridization conditions and data analysis were performed in the same way as those described in the accompanying paper.

References

1. Grifantini R, Sebastian S, Frigimelica E, Draghi M, Bartolini E, et al. (2003) Identification of iron-activated and -repressed Fur-dependent genes by transcriptome analysis of *Neisseria meningitidis* group B. PNAS 100: 9542-9547.
2. Ducey TF, Carson MB, Orvis J, Stintzi AP, Dyer DW (2005) Identification of the iron-responsive genes of *Neisseria gonorrhoeae* by microarray analysis in defined medium. J Bacteriol 187: 4865-4874.
3. Bindereif A, Neilands JB (1985) Promoter mapping and transcriptional regulation of the iron assimilation system of plasmid ColV-K30 in *Escherichia coli* K-12. J Bacteriol 162: 1039-1046.
4. Bagg A, Neilands JB (1987) Ferric uptake regulation protein acts as a repressor, employing iron (II) as a cofactor to bind the operator of an iron transport operon in *Escherichia coli*. Biochemistry 26: 5471-5477.
5. de Lorenzo V, Wee S, Herrero M, Neilands JB (1987) Operator sequences of the aerobactin operon of plasmid ColV-K30 binding the ferric uptake regulation (*fur*) repressor. J Bacteriol 169: 2624-2630.
6. Delany I, Grifantini R, Bartolini E, Rappuoli R, Scarlato V (2006) Effect of *Neisseria meningitidis fur* mutations on global control of gene transcription. J Bacteriol 188: 2483-2492.
7. Dubrac S, Touati D (2000) Fur positive regulation of iron superoxide dismutase in *Escherichia coli*: functional analysis of the *sodB* promoter. J Bacteriol 182: 3802-3808.
8. Zheng M, Doan B, Schneider TD, Storz G (1999) OxyR and SoxRS regulation of *fur*. J Bacteriol 181: 4639-4643.
9. Watnick PI, Eto T, Takahashi H, Calderwood SB (1997) Purification of *Vibrio cholerae fur* and estimation of its intracellular abundance by antibody sandwich enzyme-linked immunosorbent assay. J Bacteriol 179: 243-247.
10. Tsai J, Dyer DW, Sparling PF (1988) Loss of transferrin receptor activity in *Neisseria meningitidis* correlates with inability to use transferrin as an iron source. Infect Immun 56: 3132-3138.
11. Schryvers AB, Morris LJ (1988) Identification and characterization of the human lactoferrin-binding protein from *Neisseria meningitidis*. Infect Immun 56: 1144-1149.
12. Dyer DW, West EP, McKenna W, Thompson SA, Sparling PF (1988) A pleiotropic iron-uptake mutant of *Neisseria meningitidis* lacks a 70-kilodalton iron-regulated protein. Infect Immun 56: 977-983.
13. Stojiljkovic I, Srinivasan N (1997) *Neisseria meningitidis tonB*, *exbB*, and *exbD* genes: Ton-dependent utilization of protein-bound iron in Neisseriae. J Bacteriol 179: 805-812.
14. Carson SDB, Klebba PE, Newton SMC, Sparling PF (1999) Ferric Enterobactin Binding and Utilization by *Neisseria gonorrhoeae*. J Bacteriol 181: 2895-2901.
15. Larsen RA, Thomas MG, Postle K (1999) Protonmotive force, ExbB and ligand-bound FepA drive conformational changes in TonB. Mol Microbiol 31: 1809-1824.
16. Stojiljkovic I, Larson J, Hwa V, Anic S, So M (1996) HmbR outer membrane receptors of pathogenic *Neisseria* spp.: iron-regulated, hemoglobin-binding proteins with a high level of primary structure conservation. J Bacteriol 178: 4670-4678.

17. Thompson SA, Wang LL, Sparling PF (1993) Cloning and nucleotide sequence of *frpC*, a second gene from *Neisseria meningitidis* encoding a protein similar to RTX cytotoxins. Mol Microbiol 9: 85-96.
18. Thompson SA, Sparling PF (1993) The RTX cytotoxin-related FrpA protein of *Neisseria meningitidis* is secreted extracellularly by meningococci and by HlyBD+ Escherichia coli. Infect Immun 61: 2906-2911.
19. Guibourdenche M, Hoiby EA, Riou JY, Varaine F, Joguet C, et al. (1996) Epidemics of serogroup A *Neisseria meningitidis* of subgroup III in Africa, 1989-94. Epidemiol Infect 116: 115-120.
20. Osicka R, Kalmusova J, Krizova P, Sebo P (2001) *Neisseria meningitidis* RTX protein FrpC induces high levels of serum antibodies during invasive disease: polymorphism of *frpC* alleles and purification of recombinant FrpC. Infect Immun 69: 5509-5519.
21. Welch RA, Bauer ME, Kent AD, Leeds JA, Moayeri M, et al. (1995) Battling against host phagocytes: the wherefore of the RTX family of toxins? Infect Agents Dis 4: 254-272.
22. Lin W, Fullner KJ, Clayton R, Sexton JA, Rogers MB, et al. (1999) Identification of a *Vibrio cholerae* RTX toxin gene cluster that is tightly linked to the cholera toxin prophage. Proc Natl Acad Sci U S A 96: 1071-1076.
23. Ieva R, Alaimo C, Delany I, Spohn G, Rappuoli R, et al. (2005) CrgA is an inducible LysR-type regulator of *Neisseria meningitidis*, acting both as a repressor and as an activator of gene transcription. J Bacteriol 187: 3421-3430.
24. Masse E, Gottesman S (2002) A small RNA regulates the expression of genes involved in iron metabolism in *Escherichia coli*. Proc Natl Acad Sci U S A 99: 4620-4625.
25. Geissmann TA, Touati D (2004) Hfq, a new chaperoning role: binding to messenger RNA determines access for small RNA regulator. Embo J 23: 396-405.
26. Kahler CM, Martin LE, Shih GC, Rahman MM, Carlson RW, et al. (1998) The (alpha2-->8)-linked polysialic acid capsule and lipooligosaccharide structure both contribute to the ability of serogroup B *Neisseria meningitidis* to resist the bactericidal activity of normal human serum. Infect Immun 66: 5939-5947.
27. Ram S, Mackinnon FG, Gulati S, McQuillen DP, Vogel U, et al. (1999) The contrasting mechanisms of serum resistance of *Neisseria gonorrhoeae* and group B *Neisseria meningitidis*. Mol Immunol 36: 915-928.
28. Koomey JM, Gill RE, Falkow S (1982) Genetic and biochemical analysis of gonococcal IgA1 protease: cloning in *Escherichia coli* and construction of mutants of gonococci that fail to produce the activity. Proc Natl Acad Sci U S A 79: 7881-7885.
29. Lin L, Ayala P, Larson J, Mulks M, Fukuda M, et al. (1997) The *Neisseria* type 2 IgA1 protease cleaves LAMP1 and promotes survival of bacteria within epithelial cells. Mol Microbiol 24: 1083-1094.
30. Hauck CR, Meyer TF (1997) The lysosomal/phagosomal membrane protein h-lamp-1 is a target of the IgA1 protease of *Neisseria gonorrhoeae*. FEBS Lett 405: 86-90.
31. Vitovski S, Read RC, Sayers JR (1999) Invasive isolates of *Neisseria meningitidis* possess enhanced immunoglobulin A1 protease activity compared to colonizing strains. Faseb J 13: 331-337.
32. Newcombe J, Jeynes JC, Mendoza E, Hinds J, Marsden GL, et al. (2005) Phenotypic and transcriptional characterization of the meningococcal PhoPQ system, a magnesium-sensing two-component regulatory system that controls genes involved in remodeling the meningococcal cell surface. J Bacteriol 187: 4967-4975.
33. Dunn KL, Farrant JL, Langford PR, Kroll JS (2003) Bacterial [Cu,Zn]-cofactored superoxide dismutase protects opsonized, encapsulated *Neisseria meningitidis* from phagocytosis by human monocytes/macrophages. Infect Immun 71: 1604-1607.

34. Wilks KE, Dunn KL, Farrant JL, Reddin KM, Gorringe AR, et al. (1998) Periplasmic superoxide dismutase in meningococcal pathogenicity. Infect Immun 66: 213-217.
35. Mellin JR, Goswami S, Grogan S, Tjaden B, Genco CA (2007) A Novel Fur and Iron-regulated Small RNA, NrrF, is Required for Indirect Fur-Mediated Regulation of the *sdhA* and *sdhC* genes in *Neisseria meningitidis*. J Bacteriol.
36. Ren J, Sainsbury S, Combs SE, Capper RG, Jordan PW, et al. (2007) The structure and transcriptional analysis of a global regulator from *Neisseria meningitidis*. J Biol Chem.
37. Escolar L, Perez-Martin J, de Lorenzo V (1998) Binding of the *fur* (ferric uptake regulator) repressor of *Escherichia coli* to arrays of the GATAAT sequence. J Mol Biol 283: 537-547.
38. Palyada K, Threadgill D, Stintzi A (2004) Iron acquisition and regulation in *Campylobacter jejuni*. J Bacteriol 186: 4714-4729.
39. Grifantini R, Frigimelica E, Delany I, Bartolini E, Giovinazzi S, et al. (2004) Characterization of a novel *Neisseria meningitidis* Fur and iron-regulated operon required for protection from oxidative stress: utility of DNA microarray in the assignment of the biological role of hypothetical genes. Mol Microbiol 54: 962-979.
40. Wang G, Maier RJ (2004) An NADPH quinone reductase of *Helicobacter pylori* plays an important role in oxidative stress resistance and host colonization. Infect Immun 72: 1391-1396.
41. Clark VL, Campbell LA, Palermo DA, Evans TM, Klimpel KW (1987) Induction and repression of outer membrane proteins by anaerobic growth of *Neisseria gonorrhoeae*. Infect Immun 55: 1359-1364.
42. Lissenden S, Mohan S, Overton T, Regan T, Crooke H, et al. (2000) Identification of transcription activators that regulate gonococcal adaptation from aerobic to anaerobic or oxygen-limited growth. Mol Microbiol 37: 839-855.
43. Mellies J, Jose J, Meyer TF (1997) The *Neisseria gonorrhoeae* gene *aniA* encodes an inducible nitrite reductase. Mol Gen Genet 256: 525-532.
44. Whitehead RN, Overton TW, Snyder LA, McGowan SJ, Smith H, et al. (2007) The small FNR regulon of *Neisseria gonorrhoeae*: comparison with the larger *Escherichia coli* FNR regulon and interaction with the NarQ-NarP regulon. BMC Genomics 8: 35.
45. Ahmed S (2005) LPS Mediated Serum Resistance in *Neisseria meningitidis*. Oxford.
46. Kellogg DS, Jr., Peacock WL, Jr., Deacon WE, Brown L, Pirkle DI (1963) *Neisseria Gonorrhoeae*. I. Virulence Genetically Linked To Clonal Variation. J Bacteriol 85: 1274-1279.

Figure 1: Venn diagrams highlighting the number of genes that are similarly and differently identified to be regulated in the response to the presence or absence of iron in the results of this study and that of Grifantini *et al*. [1]. **a** – Genes up-regulated in the presence of iron, **b** – Genes up-regulated in the absence of iron.

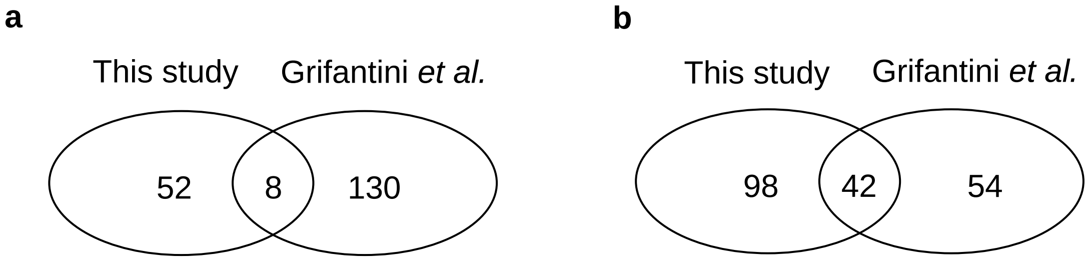

Figure 2: Venn diagrams highlighting the levels of similarity between results of this study and those of the study by Ducey *et al*. [2]. **a** – Genes up-regulated in the presence of iron, **b** – Genes up-regulated in the absence of iron.

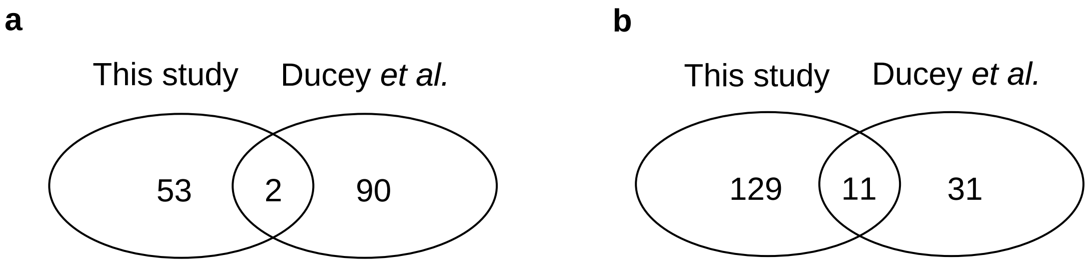

Table S1.1: Genes up-regulated in the absence of iron

Fold Ratio (Fe-/Fe+)[1]	CyberT *p*-value	NMB Synonym	Gene	Gene Annotation	Assays[2]	Consistency[3]	TIGR family
1.8	0.045	NMB0576	hemA	Glutamyl-tRNA reductase	3	a	Biosynthesis of cofactors, prosthetic groups, and carriers, Heme, porphyrin, and cobalamin
1.6	0.027	NMB1807	ponA	Penicillin-binding protein 1	4	a	Cell envelope, Biosynthesis and degradation of murein sacculus and peptidoglycan
2	<0.001	NMB0068	siaC	Polysialic acid capsule biosynthesis protein SiaC	7	a	Cell envelope, Biosynthesis and degradation of surface polysaccharides and lipopolysaccharides
1.7	0.001	NMB0069	siaB	Polysialic acid capsule biosynthesis protein SiaB	6	a	Cell envelope, Biosynthesis and degradation of surface polysaccharides and lipopolysaccharides
1.6	0.003	NMB0070	synX	Polysialic acid capsule biosynthesis protein SynX	7	a	Cell envelope, Biosynthesis and degradation of surface polysaccharides and lipopolysaccharides
1.8	0.024	NMB1994		Adhesin/invasin	4	a	Cell envelope, Surface structures
1.9	0.001	NMB0838		Transcriptional regulator, possibly cold shock related	5	a	Cellular processes, Adaptations to atypical conditions
1.7	0.036	NMB0342	ispA	Intracellular septation protein A	4	a	Cellular processes, Cell division
2.2	0.016	NMB1398	sodC	Cu-Zn-superoxide dismutase	7	b	Cellular processes, Detoxification
2.3	<0.001	NMB0364, NMB0584, NMB1412, NMB1414		FrpC operon protein	6	a	Cellular processes, Pathogenesis
2.1	<0.001	NMB0365		Iron-regulated protein FrpC	6	a	Cellular processes, Pathogenesis
1.8	<0.001	NMB0585	frpA	Hypothetical protein	7	a	Cellular processes, Pathogenesis
3	<0.001	NMB0977		Modulator of drug activity B	5	a	Cellular processes, Toxin production and resistance
2.1	0.003	NMB1210		Toxin-activating protein	4	a	Cellular processes, Toxin production and resistance
1.6	0.001	NMB1646		Haemolysin	6	a	Cellular processes, Toxin production and resistance
3.2	<0.001	NMB1857	mdaB	Modulator of drug activity B	6	a	Cellular processes, Toxin production and resistance
2	0.001	NMB1230	hupB	DNA-binding protein HU-beta	7	b	DNA metabolism, Chromosome-associated proteins
2.1	<0.001	NMB0826		C-5 cytosine-specific DNA methylase	7	a	DNA metabolism, DNA replication, recombination, and repair
1.5	0.03	NMB0831		Hypothetical protein	3	a	DNA metabolism, Restriction/modification
1.6	0.045	NMB1290		C-5 cytosine-specific DNA-methylase	7	b	DNA metabolism, Restriction/modification
1.5	0.044	NMB1375		Restriction system methylase	7	c	DNA metabolism, Restriction/modification
1.7	0.029	NMB2065		HemK protein	4	b	DNA metabolism, Restriction/modification
1.5	0.028	NMB0123		Ferredoxin, 4Fe-4S bacterial type	7	b	Energy metabolism, Electron transport
1.9	0.01	NMB0250	nuoH	NADH dehydrogenase I, H subunit	4	a	Energy metabolism, Electron transport
1.5	0.035	NMB1134, NMB1172	fdx	Ferredoxin, 2Fe-2S type	4	a	Energy metabolism, Electron transport

1.7	0.013	NMB0430		Carboxyphosphonoenolpyruvate phosphonomutase	3	a	Energy metabolism, Fermentation
2.1	<0.001	NMB1395		Alcohol dehydrogenase, zinc-containing	6	a	Energy metabolism, Fermentation
2.2	<0.001	NMB1377		L-lactate dehydrogenase	5	a	Energy metabolism, Glycolysis/gluconeogenesis
1.6	0.029	NMB0340	gloA	Lactoylglutathione lyase	5	b	Energy metabolism, Other
1.5	0.001	NMB1493	cstA	Carbon starvation protein A	7	a	Energy metabolism, Other
1.9	0.002	NMB1458	fumC	Fumarate hydratase, class II	5	a	Energy metabolism, TCA cycle
1.6	0.023	NMB0028		Hypothetical protein	5	a	Hypothetical proteins
1.7	0.038	NMB0032		Hypothetical protein	4	a	Hypothetical proteins
1.6	0.016	NMB0200		Hypothetical protein	6	a	Hypothetical proteins
1.5	0.007	NMB0252		Hypothetical protein	5	a	Hypothetical proteins
1.6	0.001	NMB0272		Hypothetical protein	6	a	Hypothetical proteins
1.8	0.032	NMB0346		Hypothetical protein	3	a	Hypothetical proteins
1.9	0.005	NMB0350		Hypothetical protein	3	a	Hypothetical proteins
1.9	0.041	NMB0376, unannotated between NMB0651/52		Hypothetical protein	4	b	Hypothetical proteins
3	0.004	NMB0417	dcaB	Hypothetical protein	3	a	Hypothetical proteins
1.7	0.003	NMB0449		Hypothetical protein	5	a	Hypothetical proteins
1.7	0.001	NMB0450		Hypothetical protein	6	a	Hypothetical proteins
1.8	0.001	NMB0467		Hypothetical protein	5	a	Hypothetical proteins
1.6	0.005	NMB0498		Hypothetical protein	6	a	Hypothetical proteins
1.7	0.015	NMB0502		Hypothetical protein	3	a	Hypothetical proteins
2.3	0.002	NMB0504, unannotated between NMB1776/77		Hypothetical protein	4	a	Hypothetical proteins
1.8	0.001	NMB0510		Hypothetical protein	7	a	Hypothetical proteins
1.6	0.004	NMB0512		Hypothetical protein	6	a	Hypothetical proteins
1.9	<0.001	NMB0644		Hypothetical protein	6	a	Hypothetical proteins
1.6	0.003	NMB0650, NMB0651		Hypothetical protein	7	a	Hypothetical proteins
1.9	0.004	NMB0654		Hypothetical protein	5	a	Hypothetical proteins
1.9	0.011	NMB0656		Hypothetical protein	4	a	Hypothetical proteins
1.6	0.003	NMB0660		Hypothetical protein	7	a	Hypothetical proteins
4	<0.001	NMB0744		Hypothetical protein	5	a	Hypothetical proteins
1.6	<0.001	NMB0856		Hypothetical protein	7	a	Hypothetical proteins
1.7	0.001	NMB0857		Hypothetical protein	7	a	Hypothetical proteins
1.8	0.001	NMB0859		Hypothetical protein	7	a	Hypothetical proteins
1.5	0.004	NMB0861		Hypothetical protein	7	b	Hypothetical proteins

1.8	<0.001	NMB0862, unannotated between NMB0865/66		Hypothetical protein	7	a	Hypothetical proteins
1.7	<0.001	NMB0863		Hypothetical protein	7	a	Hypothetical proteins
1.8	<0.001	NMB0864		Hypothetical protein	6	a	Hypothetical proteins
2.4	0.007	NMB0865		Hypothetical protein	5	b	Hypothetical proteins
1.7	0.001	NMB0865		Putative periplasmic protein	7	a	Hypothetical proteins
2.6	<0.001	NMB0866		Hypothetical protein	7	a	Hypothetical proteins
2.3	0.002	NMB0904		Hypothetical protein	3	a	Hypothetical proteins
1.6	0.037	NMB0969, NMB1769		IS1016C2 transposase	4	a	Hypothetical proteins
1.7	<0.001	NMB1008		Hypothetical protein	6	a	Hypothetical proteins
1.5	0.003	NMB1020		Hypothetical protein	7	b	Hypothetical proteins
2.9	<0.001	NMB1107		Hypothetical protein	6	a	Hypothetical proteins
1.7	0.004	NMB1124, NMB1162		Hypothetical protein	6	a	Hypothetical proteins
1.6	0.05	NMB1132, NMB1170		Hypothetical protein	3	a	Hypothetical proteins
2	0.015	NMB1330		Hypothetical protein	5	b	Hypothetical proteins
1.6	0.001	NMB1350		Hypothetical protein	7	a	Hypothetical proteins
1.8	0.036	NMB1410		Hypothetical protein	3	a	Hypothetical proteins
1.7	0.008	NMB1490		Hypothetical protein	5	a	Hypothetical proteins
1.6	0.016	NMB1545, NMB1632		Hypothetical protein	5	a	Hypothetical proteins
1.7	0.001	NMB1597		Hypothetical protein	6	a	Hypothetical proteins
3	<0.001	NMB1598		Hypothetical protein	6	a	Hypothetical proteins
2.3	<0.001	NMB1599		Hypothetical protein	6	a	Hypothetical proteins
1.5	0.002	NMB1771		Hypothetical protein	6	a	Hypothetical proteins
1.6	0.021	NMB1777		Hypothetical protein	5	a	Hypothetical proteins
2.3	<0.001	NMB1854		Hypothetical protein	7	a	Hypothetical proteins
2.2	0.005	NMB1915		Hypothetical protein	3	a	Hypothetical proteins
1.7	0.025	NMB2014		Hypothetical protein	6	b	Hypothetical proteins
1.9	<0.001	NMB2085		Hypothetical protein	7	a	Hypothetical proteins
1.5	0.022	NMB2118		Hypothetical protein	5	a	Hypothetical proteins
2.4	0.002	NMB2152		Hypothetical protein	5	a	Hypothetical proteins
2.2	0.002	unannotated between NMB0247/48		Hypothetical protein	4	a	Hypothetical proteins
1.6	0.003	unannotated between NMB0349/50		Hypothetical protein	5	a	Hypothetical proteins
1.6	0.007	unannotated between NMB0583/84 and NMB1411/12		Hypothetical protein	6	b	Hypothetical proteins
2.1	<0.001	unannotated between NMB0649/50		Hypothetical protein	5	a	Hypothetical proteins

1.6	0.01	unannotated between NMB1396/97		Hypothetical protein	5	b	Hypothetical proteins
3.3	0.001	NMB0035		Conserved hypothetical protein	6	a	Hypothetical proteins, Conserved
1.8	0.001	NMB0047		Conserved hypothetical protein	7	b	Hypothetical proteins, Conserved
1.5	0.019	NMB0415	dcaA	Conserved hypothetical protein	3	a	Hypothetical proteins, Conserved
2	0.007	NMB0486, NMB0970, NMB1741		Conserved hypothetical protein	4	a	Hypothetical proteins, Conserved
1.9	0.003	NMB0562		Conserved hypothetical protein	5	a	Hypothetical proteins, Conserved
1.9	0.007	NMB0571		Conserved hypothetical protein	3	a	Hypothetical proteins, Conserved
2.4	0.003	NMB0599		Conserved hypothetical protein	4	a	Hypothetical proteins, Conserved
1.8	0.001	NMB1026		Conserved hypothetical protein	5	a	Hypothetical proteins, Conserved
2.8	0.002	NMB1059		Conserved hypothetical protein	6	b	Hypothetical proteins, Conserved
1.8	0.031	NMB1336		Conserved hypothetical protein	4	b	Hypothetical proteins, Conserved
1.9	0.008	NMB1550, NMB1627		Conserved hypothetical protein	4	a	Hypothetical proteins, Conserved
1.5	0.005	NMB1726		Conserved hypothetical protein	6	a	Hypothetical proteins, Conserved
2.1	<0.001	NMB1796		Conserved hypothetical protein	5	a	Hypothetical proteins, Conserved
1.6	0.011	NMB1890		Conserved hypothetical protein	6	c	Hypothetical proteins, Conserved
2	<0.001	NMB1979		Conserved hypothetical protein	7	a	Hypothetical proteins, Conserved
1.8	<0.001	NMB2078		Conserved hypothetical protein	6	a	Hypothetical proteins, Conserved
1.9	0.001	NMB0748	hfq	Host factor-I	7	a	Mobile and extrachromosomal element functions, Prophage functions
1.9	0.012	NMB1083		Bacteriophage DNA transposition protein B	3	a	Mobile and extrachromosomal element functions, Prophage functions
1.8	0.002	NMB0700	iga	IgA-specific serine endopeptidase	5	a	Protein fate, Degradation of proteins, peptides, and glycopeptides
1.5	0.045	NMB0922		Alpha-2,3-sialyltransferase	5	b	Protein fate, Protein modification and repair
1.6	0.005	NMB0147	rplV	50S ribosomal protein L22	7	a	Protein synthesis, Ribosomal proteins: synthesis and modification
1.5	0.006	NMB0152	rplN	50S ribosomal protein L14	7	b	Protein synthesis, Ribosomal proteins: synthesis and modification
1.5	0.006	NMB0164	rpmJ	50S ribosomal protein L36	5	a	Protein synthesis, Ribosomal proteins: synthesis and modification
1.9	<0.001	NMB0163	infA	Translation initiation factor IF-1	7	a	Protein synthesis, Translation factors
1.6	0.004	NMB2084	cysS	Cysteinyl-tRNA synthetase	7	a	Protein synthesis: tRNA aminoacylation
1.8	<0.001	NMB0646		Ribonuclease inhibitor barstar	7	a	Regulatory functions, Other
1.6	0.043	NMB1007		Putative phage repressor	4	a	Regulatory functions, Other
1.8	0.007	NMB1538	rpoD	RNA polymerase sigma factor RpoD	4	a	Transcription, Transcription factors
1.7	0.007	NMB0072	ctrB	Capsule polysaccharide export inner-membrane protein CtrB	5	a	Transport and binding proteins, Carbohydrates, organic alcohols, and acids

2.1	0.007	NMB0074	ctrD	Capsule polysaccharide export ATP-binding protein CtrD	3	a	Transport and binding proteins, Carbohydrates, organic alcohols, and acids
2.1	0.01	NMB0293		TonB-dependent receptor	3	a	Transport and binding proteins, Cations and iron carrying compounds
8.2	<0.001	NMB0460	tbpB	Transferrin-binding protein B	6	a	Transport and binding proteins, Cations and iron carrying compounds
2.5	0.001	NMB0461	tbpA	Transferrin-binding protein A	4	a	Transport and binding proteins, Cations and iron carrying compounds
2.5	0.023	NMB1988	frpB	Iron-regulated outer membrane protein FrpB	4	b	Transport and binding proteins, Cations and iron carrying compounds
2.3	<0.001	NMB1728	exbD	Biopolymer transport protein ExbD	7	a	Transport and binding proteins, Other
2.8	0.001	NMB1729	exbB	Biopolymer transport protein ExbB	3	a	Transport and binding proteins, Other
1.6	0.006	NMB1429	porA	Outer membrane protein PorA	7	a	Transport and binding proteins, Porins
1.7	<0.001	NMB1732		Transporter, putative	7	a	Transport and binding proteins, Unknown substrate
1.8	0.005	NMB0097		Secretion protein	6	a	Unknown function, General
1.5	0.011	NMB2009		ATP-dependent RNA helicase HrpA	3	a	Unknown function, General
2	0.002	NMB0344		BolA-YrbA family protein	4	a	Unknown function, General
2	<0.001	NMB0412		Cell division protein FtsL-related protein	6	a	Unknown function, General
1.8	0.039	NMB1403		FrpA/C-related protein	3	a	Unknown function, General
1.8	<0.001	NMB2008		ABC transporter, ATP-binding protein-related protein	7	a	Unknown function, General

[1] Fold ratio is the relative transcript abundance in the absence of iron compared to the presence of iron.
[2] The number of comparisons in which this gene was reliably detected.
[3] A measure of the number of comparisons in which the gene was changed in the same direction. a-all one direction, b-one in opposite direction, c-two in opposite direction.

Table S1.2: Genes up-regulated in the presence of iron

Fold Ratio (Fe+/Fe-)[1]	CyberT *p*-value	NMB Synonym	Gene	Gene Annotation	Assays[2]	Consistency[3]	TIGR family
1.8	0.002	NMB1498	lysC	Aspartokinase, alpha and beta subunits	6	a	Amino acid biosynthesis, Aspartate family
1.8	0.025	NMB1070	leuA	2-isopropylmalate synthase	4	a	Amino acid biosynthesis, Pyruvate family
1.9	<0.001	NMB1150, NMB1188	ilvD	Dihydroxy-acid dehydratase	7	a	Amino acid biosynthesis, Pyruvate family
1.6	<0.001	NMB1574	ilvC	Ketol-acid reductoisomerase	7	a	Amino acid biosynthesis, Pyruvate family
1.7	0.002	NMB0011	murA	UDP-N-acetylglucosamine 1-carboxyvinyltransferase	5	a	Cell envelope, Biosynthesis and degradation of murein sacculus and peptidoglycan
1.8	<0.001	NMB2090	gmhA	Phosphoheptose isomerase	7	a	Cell envelope, Biosynthesis and degradation of surface polysaccharides and lipopolysaccharides
1.5	0.014	NMB0333	pilG	Pilus assembly protein PilG	7	a	Cell envelope, Surface structures
1.7	0.001	NMB1462		Transglycosylase	6	a	Central intermediary metabolism, Other
2.2	0.003	NMB0208		Ferredoxin, 4Fe-4S bacterial type	4	a	Energy metabolism, Electron transport
2.2	0.014	NMB0241	nuoA	NADH dehydrogenase I, A subunit	4	a	Energy metabolism, Electron transport
1.8	0.001	NMB1725	fixN	Cytochrome c oxidase, subunit I	6	a	Energy metabolism, Electron transport
2.6	<0.001	NMB1968	aldA	Aldehyde dehydrogenase A	5	a	Energy metabolism, Fermentation
1.8	0.002	NMB1060	fbp	Fructose-1,6-bisphosphatase	5	a	Energy metabolism, Glycolysis/gluconeogenesis
2	0.004	NMB1997	hagH	Hydroxyacylglutathione hydrolase	5	a	Energy metabolism, Other
1.5	0.032	NMB1341	pdhA	Pyruvate dehydrogenase, E1 component	6	b	Energy metabolism, Pyruvate dehydrogenase
1.5	0.038	NMB0949	sdhD	Succinate dehydrogenase, membrane anchor protein	4	a	Energy metabolism, TCA cycle
1.7	0.049	NMB0950	sdhA	Succinate dehydrogenase, flavoprotein subunit	7	c	Energy metabolism, TCA cycle
1.6	0.003	NMB0951	sdhB	Succinate dehydrogenase, iron-sulfur protein	7	b	Energy metabolism, TCA cycle
1.7	0.006	NMB1592		Hypothetical protein	7	b	Hypothetical proteins
1.8	0.006	NMB0057, NMB0299, NMB1657, NMB2017		ComEA-related protein	6	b	Hypothetical proteins
2.9	<0.001	NMB1035		Hypothetical protein	6	a	Hypothetical proteins
4.1	<0.001	NMB1048		Hypothetical protein	7	a	Hypothetical proteins
2.1	<0.001	NMB1126, NMB1164		Hypothetical protein	5	a	Hypothetical proteins
1.7	0.004	NMB1245		Hypothetical protein	6	b	Hypothetical proteins
1.6	0.002	NMB1503		Hypothetical protein	6	a	Hypothetical proteins
1.9	<0.001	NMB1523, NMB1533		Lip / H8 outer membrane protein	7	a	Hypothetical proteins
2.1	0.007	NMB1534		Hypothetical protein	7	b	Hypothetical proteins
1.6	0.041	NMB1875		Hypothetical protein	3	a	Hypothetical proteins
2	0.045	NMB1899		Hypothetical protein	3	a	Hypothetical proteins

1.6	0.012	unannotated between NMB1277/78		Hypothetical protein	7	a	Hypothetical proteins
1.7	0.001	unannotated between NMB1986/87		Hypothetical protein	7	b	Hypothetical proteins
1.6	0.041	NMB0436		Conserved hypothetical protein	7	b	Hypothetical proteins, Conserved
1.6	0.013	NMB1306		Conserved hypothetical protein	4	a	Hypothetical proteins, Conserved
2.2	0.001	NMB1378		Conserved hypothetical protein	6	b	Hypothetical proteins, Conserved
1.6	0.02	NMB1438		Conserved hypothetical protein	6	b	Hypothetical proteins, Conserved
1.7	0.018	NMB1475		Conserved hypothetical protein	6	b	Hypothetical proteins, Conserved
1.8	0.022	NMB1620		Conserved hypothetical protein	3	a	Hypothetical proteins, Conserved
1.7	0.006	NMB1951		Conserved hypothetical protein	4	a	Hypothetical proteins, Conserved
1.5	0.012	NMB2089		Conserved hypothetical protein	6	b	Hypothetical proteins, Conserved
2.1	<0.001	NMB2140		Conserved hypothetical protein	7	a	Hypothetical proteins, Conserved
2.6	<0.001	NMB2142		Conserved hypothetical protein	6	a	Hypothetical proteins, Conserved
1.5	0.012	NMB1312	clpP	ATP-dependent Clp protease, proteolytic subunit	7	a	Protein fate, Degradation of proteins, peptides, and glycopeptides
2.7	0.001	NMB0550	dsbC	Thiol:disulfide interchange protein DsbC	3	a	Protein fate, Protein folding and stabilization
1.5	0.011	NMB1972	groEL	Chaperonin, 60 kDa	7	b	Protein fate, Protein folding and stabilization
1.5	0.02	NMB0876	rplY	50S ribosomal protein L25	7	c	Protein synthesis, Ribosomal proteins: synthesis and modification
1.7	<0.001	NMB1321	rpsR	30S ribosomal protein S18	6	a	Protein synthesis, Ribosomal proteins: synthesis and modification
1.7	<0.001	NMB1323	rpsF	30S ribosomal protein S6	7	a	Protein synthesis, Ribosomal proteins: synthesis and modification
1.9	<0.001	NMB2057	rplM	50S ribosomal protein L13	7	a	Protein synthesis, Ribosomal proteins: synthesis and modification
1.5	0.003	NMB0875	prsA	Ribose-phosphate pyrophosphokinase	7	b	Purines, pyrimidines, nucleosides, and nucleotides, Purine ribonucleotide biosynthesis
2.3	<0.001	NMB0398		Transcriptional regulator, ArsR family	6	a	Regulatory functions, Other
2.5	<0.001	16S rRNA		16S ribosomal RNA sequence	7	a	Ribosomal RNA
1.9	<0.001	23S rRNA		23S ribosomal RNA sequence	7	a	Ribosomal RNA
1.5	0.045	NMB0683	nusB	N utilization substance protein B	5	a	Transcription, Transcription factors
1.6	0.008	NMB1647		Amino acid symporter	6	a	Transport and binding proteins, Amino acids, peptides and amines
3.7	<0.001	NMB1206	bfrB	Bacterioferritin B	7	a	Transport and binding proteins, Cations and iron carrying compounds
3.4	<0.001	NMB1207	bfrA	Bacterioferritin A	7	a	Transport and binding proteins, Cations and iron carrying compounds

[1] Fold ratio is the relative transcript abundance in the presence of iron compared to the absence of iron.

[2] The number of comparisons in which this gene was reliably detected.
[3] A measure of the number of comparisons in which the gene was changed in the same direction. a-all one direction, b-one in opposite direction, c-two in opposite direction.

Table 3: Comparison of qRT-PCR and microarray transcript fold ratios. Fold changes shown are ((iron-replete transcript level)/(iron-deplete transcript level)).

Gene	qRT-PCR fold ratio	Microarray fold ratio
NMB0205	-1.5	-1.4
NMB0460	-5.7	-8.2
NMB0700	-5.8	-1.8
NMB0956	1.0	1.1
NMB1150	2.2	1.9
NMB1206	1.5	3.6
NMB1856	-8.6	n/a
NMB1857	-7.4	-3.2

Table 4: Iron regulated transcriptional units and the presence of putative Fur sites.

Transcriptional Unit[1]	Regulation by presence (+) or absence (-) of iron[2]	Position of Fur site[3]
NMB0011	+	
NMB0028-NMB0027	-	-34
NMB0032	-	
NMB0034-NMB0035	-	
NMB0047	-	
NMB0057	+	
NMB0070-NMB0067	-	-41
NMB0071-NMB0074	-	-71
NMB0096-NMB0098	-	+125
NMB0123	-	
NMB0136-NMB0169	-	
NMB0200	-	+66
NMB0208	+	
NMB0241-NMB0245	+	-119
NMB0249-NMB0254	-	
NMB0272-NMB0273	-	
NMB0293-NMB0294	-	+9
NMB0299	+	
NMB0333-NMB0330	+	
NMB0338-NMB0340	-	+185
NMB0342-NMB0345	-	
NMB0346	-	
NMB0350	-	
NMB0364-NMB0365	-	-286
NMB0377-NMB0373	-	
NMB0398-NMB0399	+	-58
NMB0410-NMB0415	-	-228
NMB0416-NMB0417	-	
NMB0430-NMB0433	-	-113
NMB0436	+	
NMB0449	-	
NMB0450-NMB0451	-	
NMB0460-NMB0461	-	-19
NMB0467	-	
NMB0486-NMB0482	-	
NMB0498-NMB0500	-	
NMB0501-NMB0503	-	

NMB0504	-	
NMB0509-NMB0510	-	
NMB0511-NMB0513	-	-196
NMB0550	+	
NMB0563-NMB0562	-	
NMB0573-NMB0571	-	
NMB0576	-	
NMB0584-NMB0585	-	-285
NMB0603-NMB0596	-	
NMB0643-NMB0644	-	
NMB0645-NMB0646	-	
NMB0649-NMB0651	-	
NMB0652-NMB0654	-	
NMB0655-NMB0656	-	-199
NMB0660	-	-249
NMB0685-NMB0683	+	
NMB0700	-	
NMB0744	-	
NMB0748	-	
NMB0826-NMB0827	-	
NMB0831-NMB0833	-	
NMB0838	-	
NMB0863-NMB0856	-	+102
NMB0866-NMB0864	-	
NMB0875	+	
NMB0876	+	
NMB0908-NMB0901	-	-91
NMB0922	-	
NMB0948-NMB0954	+	
NMB0969	-	
NMB0970-NMB0974	-	
NMB0977	-	
NMB1007-NMB1011	-	
NMB1021-NMB1018	-	
NMB1026-NMB1028	-	
NMB1036-NMB1034	+	
NMB1048	+	
NMB1059	-	
NMB1060	+	
NMB1070	+	
NMB1083-NMB1085	-	

NMB1107	-	-18
NMB1126-NMB1124	-	
NMB1131-NMB1134	-	
NMB1150	+	
NMB1164-NMB1161	+	
NMB1169-NMB1172	-	
NMB1188	+	
NMB1207-NMB1206	+	
NMB1209-NMB1210	-	
NMB1230	-	
NMB1248-NMB1245	+	
NMB1290-NMB1289	-	
NMB1306-NMB1307	+	
NMB1312	+	
NMB1323-NMB1320	+	
NMB1330	-	-29, +46
NMB1334-NMB1337	-	
NMB1340-NMB1344	+	
NMB1350	-	+51
NMB1375-NMB1376	-	
NMB1377	-	-40
NMB1378-NMB1379	+	-224
NMB1395	-	-91
NMB1398	-	
NMB1402-NMB1405	-	-59
NMB1410	-	-7, +38
NMB1412	-	-285
NMB1414-NMB1415	-	
NMB1429	-	-143
NMB1436-NMB1438	+	-152
NMB1458	-	
NMB1462	+	
NMB1475	+	
NMB1490	-	
NMB1493-NMB1494	-	
NMB1498	+	
NMB1503-NMB1505	+	
NMB1523-NMB1524	+	
NMB1534-NMB1531	+	
NMB1535-NMB1538	-	
NMB1543-NMB1545	-	

NMB1549-NMB1551	-	
NMB1577-NMB1574	+	
NMB1592	+	
NMB1595-NMB1597	-	
NMB1598-NMB1600	-	-342, -160, +32, +54, +126, +152
NMB1619-NMB1620	+	
NMB1627-NMB1626	-	
NMB1634-NMB1632	-	
NMB1646	-	
NMB1647	+	
NMB1657	+	
NMB1725-NMB1722	+	
NMB1726-NMB1727	-	
NMB1730-NMB1728	-	-8
NMB1732	-	
NMB1741-NMB1745	-	
NMB1769	-	
NMB1773-NMB1771	-	
NMB1780-NMB1777	-	
NMB1796	-	-37
NMB1807	-	
NMB1854-NMB1855	-	
NMB1857	-	
NMB1875-NMB1876	+	
NMB1890-NMB1891	-	
NMB1898-NMB1900	+	
NMB1914-NMB1915	-	
NMB1950-NMB1951	+	
NMB1968	+	
NMB1973-NMB1972	+	
NMB1978-NMB1981	-	
NMB1988	-	-54
NMB1994	-	
NMB1996-NMB1997	+	
NMB2007-NMB2009	-	
NMB2014-NMB2012	-	
NMB2017	+	
NMB2057-NMB2056	+	
NMB2066-NMB2065	-	
NMB2078	-	
NMB2086-NMB2083	-	

NMB2089-NMB2092	+	
NMB2117-NMB2118	-	-79
NMB2140-NMB2145	+	-228
NMB2152	-	

[1] Transcriptional units were determined *in silico*. See text for details.
[2] Indicates whether the transcriptional unit contains one or more genes up-regulated in the presence or absence of iron.
[3] Position of putative Fur binding site relative to the initiation codon. Fur sites were determined by presence of $(NATWAT)_3$ sequence with up to 3 mismatches.

Table 5: Genes consistently up-regulated during iron restriction in both this study and that of Grifantini *et al*. [1].

NMB Synonym	Gene	Gene Annotation
NMB0035		Conserved hypothetical protein
NMB0082	lipA	Capsule polysaccharide modification protein LipA
NMB0132	rpoB	DNA-directed RNA polymerase, beta subunit
NMB0152	rplN	50S ribosomal protein L14
NMB0163	infA	Translation initiation factor IF-1
NMB0164	rpmJ	50S ribosomal protein L36
NMB0250	nuoH	NADH dehydrogenase I, H subunit
NMB0340	gloA	Lactoylglutathione lyase
NMB0364		FrpC operon protein
NMB0365	frpC	FrpC protein
NMB0394		Quinolinate synthetase A
NMB0415		Conserved hypothetical protein
NMB0430		Carboxyphosphonoenolpyruvate phosphonomutase
NMB0460	tbp2	Transferrin-binding protein 2
NMB0461	tbp1	Transferrin-binding protein 1
NMB0467		Hypothetical protein
NMB0584		FrpC operon protein
NMB0613		Hypothetical protein
NMB0617	rho	Transcription termination factor Rho
NMB0721	infC	Translation initiation factor 3
NMB0744		Hypothetical protein
NMB0803		Conserved hypothetical protein
NMB0838		Cold-shock domain family protein
NMB0861		Hypothetical protein
NMB0864		Hypothetical protein

NMB0865		Hypothetical protein
NMB0977		Modulator of drug activity B
NMB1043		Hypothetical protein
NMB1255		Glycosyltransferase
NMB1377	lldD	L-lactate dehydrogenase
NMB1395		Alcohol dehydrogenase, zinc-containing
NMB1412		FrpC operon protein
NMB1414		FrpC operon protein
NMB1458	fumC	Fumarate hydratase, class II
NMB1490		Hypothetical protein
NMB1541	lbpB	Lactoferrin-binding protein B
NMB1728	exbD	Biopolymer transport protein ExbD
NMB1796		Conserved hypothetical protein
NMB1857	mdaB	Modulator of drug activity B
NMB1911	rpmF	50S ribosomal protein L32
NMB1988	frpB	Iron-regulated outer membrane protein FrpB
NMB2078		Conserved hypothetical protein

Table 6: Genes up-regulated in the presence of iron in both this study and that of Grifantini *et al*. [1].

**NMB Synonym**	**Gene**	**Gene Annotation**
NMB0398		Transcriptional regulator, ArsR family
NMB0884	sodB	Superoxide dismutase
NMB1206	bfrB	Bacterioferritin B
NMB1207	bfrA	Bacterioferritin A
NMB1378		Conserved hypothetical protein
NMB1437		Conserved hypothetical protein
NMB1438		Conserved hypothetical protein
NMB2057	rplM	50S ribosomal protein L13

Table 7: Genes up-regulated by iron restriction in both this study and that of Ducey *et al*. [2].

NGO Synonym	NMB Synonym	Gene	Gene Annotation
NG0863	NMB1059		Conserved hypothetical protein
NG1495	NMB0461	tbpA	Transferrin-binding protein A
NG0322	NMB0744		Conserved hypothetical protein
NG0108	NMB1796		Conserved hypothetical protein
NG2093	NMB1988	frpB	Ferric enterobactin receptor
NG1652	NMB0900		Hypothetical protein
NG1657	NMB0344		Conserved hypothetical protein
NG0165	NMB0861		Conserved hypothetical protein
NG0777	NMB1230	hupB	DNA-binding protein Hu
NG1378	NMB1729	exbB	Transport protein
NG1496	NMB0460	tbpB	Transferrin-binding protein B

Table 8: Genes up-regulated in the presence of iron in both this study and that of Ducey *et al*. [2].

NGO Synonym	NMB Synonym	Gene	Gene Annotation
NG1948	NMB2140		Conserved hypothetical protein
NG1864	NMB0117		Conserved hypothetical protein
