# Supplementary material for: Host Iron Binding Proteins Acting as Niche Indicators for Neisseria meningitidis
Source: PLoS One. 2009 Apr 8;4(4):e5198. doi: 10.1371/journal.pone.0005198 (PMC2662411; doi:10.1371/journal.pone.0005198)
Supplement: Dataset S2 — (0.03 MB PDF) [file pone.0005198.s002.pdf]

Supplementary Dataset 2

Validation of microarray data using qRT-PCR

Quantitative real-time PCR (qRT-PCR) was used to validate the microarray data, analyzing transcript levels for genes significantly changed in each direction or whose expression was unchanged for each comparison. The transcripts studied are shown with the results below. The qRT-PCR results correlated well with those obtained from the microarrays (Hb-Tf, 0.98; Hb-Lf, 0.86; Tf-Lf, 0.80), indicating that the microarray results give an accurate report of transcript levels within the bacterium. qRT-PCR demonstrated that the absence of haemoglobin is associated with the up-regulation of Lrp (NMB1650) in the Hb-Lf comparison.

Table 2.1: Comparison of qRT-PCR and microarray transcript fold ratios for haemoglobin versus transferrin comparison. Fold changes shown are ((Haemoglobin transcript level)/(Transferrin transcript level)).

Gene	qRT-PCR fold ratio	Microarray fold ratio
NMB0460	-8.5	-7.2
NMB0546	7.4	4.8
NMB0700	-3.6	-2.2
NMB0740	1.9	1.9
NMB0956	1.0	1.1
NMB1622	-2.3	-1.9
NMB2039	2.2	2.2

Table 2.2: Comparison of qRT-PCR and microarray transcript fold ratios for haemoglobin versus lactoferrin comparison. Fold changes shown are ((Haemoglobin transcript level)/(Lactoferrin transcript level)).

Gene	qRT-PCR fold ratio	Microarray fold ratio
NMB0460	-2.5	-3.3
NMB0546	8.2	4.6
NMB0942	-3.1	-5.5
NMB0956	1.0	1.0
NMB0992	3.9	1.6
NMB1206	6.5	2.8
NMB1650	4.3	n/a
NMB1768	-2.3	-2.2
NMB2039	2.9	2.1

Table 2.3: Comparison of qRT-PCR and microarray transcript fold ratios for lactoferrin versus transferrin comparison. Fold changes shown are ((Lactoferrin transcript level)/(Transferrin transcript level)).

Gene	qRT-PCR fold ratio	Microarray fold ratio
NMB0460	-10.2	-3.1
NMB0700	2.3	1.6
NMB0740	-2.4	-1.5
NMB0942	3.4	4.8
NMB0956	1.0	1.1

Table 2.4: Oligonucleotide primers used for qRT-PCR

Gene	Forward Primer	Reverse Primer
NMB0205	TTGGAAGAGGGTGTGGAAAT	CGCCTTTGTCCAACTCATAA
NMB0460	CCACCACCACGCAATACTAC	CAAAGGGATGTTCCTTGGTT
NMB0546	CTGTATCGTGAGTGCCGATT	CGTAGATGGCAATCCACTGT
NMB0700	TCGTAACAGAAGTCGCACCT	TTCAAATTGCGTACCTGCTC
NMB0740	AAACCGTCAGGCAGCTTTAT	TCGCCTTGTTTAATGTCCAA
NMB0942	GGCAAAGAATATCCGCTGTT	AACGTTGGTTGAATTTGCTG
NMB0956	GCGTGCGAAGTACAAAGAAA	AACAGAAGCATTCACAACCG
NMB0992	ACCACAAACGTAACCAACGA	GAAGCTGTTGTACCGGGTTT
NMB1150	GACTTGATTGACGCGATGAT	GTTCATCGAGTTTGCCGTAA
NMB1206	AACTGAATATCGGCACGGA	TAGTCTTGAGCCTCTTCGCA
NMB1622	CGTACTTGGCACATCCAATC	CGTTGGAACTTGGGATCTTT
NMB1650	GTCGGGCGAATACGACTATC	TTTGACCTCCTCCATCACAA
NMB1768	ATTATCAGTGATGCGGTCCA	CCATGAGCGTTATCCAGTTG
NMB1856	CCGAACAAACCTCCGATAAA	ATTGTTTCCCAGTTCCTCCA
NMB1857	GACACGGCAAACTCTACCAA	CGCATTCCAAGTCAGTGAAA
NMB2039	GAACAGCGTCCTGAAAGACA	TCGTAGCGTACGGAAATGAG
