# Supplementary material for: Host Iron Binding Proteins Acting as Niche Indicators for Neisseria meningitidis
Source: PLoS One. 2009 Apr 8;4(4):e5198. doi: 10.1371/journal.pone.0005198 (PMC2662411; doi:10.1371/journal.pone.0005198)
Supplement: Table S1 — Genes up-regulated in the presence of Haemoglobin compared to Transferrin. 1 Fold ratio is the relative transcript abundance in the presence of Haemoglobin compared to the presence of Transferrin. 2 The number of comparisons in which this gene was reliably detected. 3 A measure of the number of comparisons in which the gene was changed in the same direction. a-all one direction, b-one in opposite direction, c-two in opposite direction. (0.03 MB PDF) [file pone.0005198.s003.pdf]

Table S1: Genes up-regulated in the presence of Haemoglobin compared to Transferrin

Fold Ratio Hb/Tf [1]	CyberT *p*-value	Fold Ratio (Fe-/Fe+)	NMB Synonym	Gene	Gene Annotation	Assays[2]	Consistency[3]	TIGR Family
1.6	0.01	1	NMB0745	folK	2-amino-4-hydroxy-6-hydroxymethyldihydropteridine-pyrophosphokinase	4	a	Biosynthesis of cofactors, prosthetic groups, and carriers, Folic acid
1.6	0.006	1.1	NMB2156	rfaC	Heptosyltransferase I	5	a	Cell envelope, Biosynthesis and degradation of surface polysaccharides and lipopolysaccharides
1.5	0.003	1.3	NMB0345		Cell-binding factor	5	a	Cell envelope, Other
1.7	<0.001	1.1	NMB0946		Peroxiredoxin 2 family protein-glutaredoxin	6	a	Cell envelope, Other
1.7	0.019	0.9	NMB0018	pilE	Type IV pilin class II	6	b	Cell envelope, Surface structures
1.8	0.036	1.1	NMB1484	surE	Stationary-phase survival protein SurE	3	a	Cellular processes, Adaptations to atypical conditions
1.8	0.032	1.2	NMB0442, NMB0926, NMB1465, NMB1636		Opa protein	4	b	Cellular processes, Pathogenesis
2.5	<0.001	1.1	NMB0585, NMB1403, NMB1405, NMB1407		Hypothetical protein	5	a	Cellular processes, Pathogenesis
2.5	<0.001	1.2	NMB1415	frpC	Iron-regulated protein FrpC	5	a	Cellular processes, Pathogenesis
2.3	<0.001	1.4	NMB1768		Haemagglutinin/haemolysin-related protein	5	a	Cellular processes, Toxin production and resistance
1.5	0.016	1.4	NMB0614		Oxidoreductase	4	a	Central intermediary metabolism, Other
1.8	0.014	1.1	NMB0727		N-6 adenine-specific DNA methylase	3	a	DNA metabolism, Restriction/modification
1.9	0.032	0.9	NMB1622	norB	Nitric oxide reductase	3	a	Energy metabolism, Anaerobic
1.8	0.002	1.4	NMB1366		Thioredoxin	3	a	Energy metabolism, Electron transport
1.8	0.006	1.3	NMB1696	acp-2	Acyl carrier protein	4	a	Fatty acid and phospholipid metabolism, Biosynthesis
1.8	0.001	1.3	NMB0119		Hypothetical protein	6	b	Hypothetical proteins
1.7	0.004	1.4	NMB0120		Hypothetical protein	6	b	Hypothetical proteins
1.7	0.005	1.2	NMB0129		Hypothetical protein	5	a	Hypothetical proteins
1.7	0.037	0.8	NMB0297		Hypothetical protein	6	c	Hypothetical proteins
2.2	0.001	0.3	NMB0320		Hypothetical protein	3	a	Hypothetical proteins
1.6	0.01	1.4	NMB0363, unannotated between NMB0583/84		Hypothetical protein	4	a	Hypothetical proteins
1.6	0.002	0.8	NMB0429		Hypothetical protein	6	a	Hypothetical proteins
1.8	0.012	1.3	NMB0491		Hypothetical protein	6	c	Hypothetical proteins
1.9	0.037		NMB0511		FhaB cassette	4	b	Hypothetical proteins

2.8	<0.001	1.4	NMB0858		Hypothetical protein	4	a	Hypothetical proteins
2	0.001	1.1	NMB0899		Hypothetical protein	6	b	Hypothetical proteins
1.7	<0.001	0.9	NMB0945		Hypothetical protein	6	a	Hypothetical proteins
1.6	0.003	1	NMB1006		Hypothetical protein	5	a	Hypothetical proteins
1.7	0.01	1.2	NMB1008		Hypothetical protein	5	b	Hypothetical proteins
1.5	0.023	1	NMB1056		Hypothetical protein	6	b	Hypothetical proteins
1.7	0.024	0.9	NMB1211		Hypothetical protein	5	b	Hypothetical proteins
1.6	0.007	1.3	NMB1334		Hypothetical protein	3	a	Hypothetical proteins
1.8	0.002	1.4	NMB1408		Hypothetical protein	3	a	Hypothetical proteins
1.7	0.01	0.7	NMB1746		Hypothetical protein	4	a	Hypothetical proteins
2.1	<0.001	1.2	NMB1844		Hypothetical protein	6	a	Hypothetical proteins
1.5	0.028	1	NMB1850		Hypothetical protein	4	a	Hypothetical proteins
1.6	0.014	1.2	NMB1853		Hypothetical protein	4	a	Hypothetical proteins
1.6	0.03	1.1	NMB2013		Hypothetical protein	6	b	Hypothetical proteins
2.5	0.028		NMB2073		Hypothetical protein	3	b	Hypothetical proteins
1.6	0.001	1.1	NMB2113		Hypothetical protein	6	a	Hypothetical proteins
1.6	0.007	1.1	unannotated between NMB0820/21		Hypothetical protein	5	a	Hypothetical proteins
1.7	<0.001	1.3	unannotated between NMB0863/64		Hypothetical protein	6	a	Hypothetical proteins
1.7	0.013	1.3	unannotated between NMB1563/64		Hypothetical protein	3	a	Hypothetical proteins
2.3	<0.001		NMB0034		Conserved hypothetical protein	4	a	Hypothetical proteins, Conserved
1.9	0.037		NMB0121		Conserved hypothetical protein	3	a	Hypothetical proteins, Conserved
2.5	<0.001	1.3	NMB0317		Conserved hypothetical protein	5	a	Hypothetical proteins, Conserved
1.8	0.003	1.4	NMB1731		Conserved hypothetical protein	4	a	Hypothetical proteins, Conserved
1.5	0.044		NMB1923		Conserved hypothetical protein	3	a	Hypothetical proteins, Conserved
3.1	0.049		NMB1081		Transposase	3	b	Mobile and extrachromosomal element functions, Prophage functions
1.6	0.006	1.4	NMB0162	secY	Preprotein translocase SecY subunit	6	b	Protein fate, Protein and peptide secretion and trafficking
1.8	0.002	1.4	NMB0561	grpE	GrpE protein	4	a	Protein fate, Protein folding and stabilization
1.8	<0.001	1.2	NMB0791		Peptidyl-prolyl cis-trans isomerase	6	a	Protein fate, Protein folding and stabilization
1.6	0.002	1.3	NMB0130	rplJ	50S ribosomal protein L10	5	a	Protein synthesis, Ribosomal proteins: synthesis and modification
1.7	0.006	1.4	NMB0131	rplL	50S ribosomal protein L7-L12	6	b	Protein synthesis, Ribosomal proteins: synthesis and modification
1.5	0.005	1.1	NMB0143	rplD	50S ribosomal protein L4	5	a	Protein synthesis, Ribosomal proteins: synthesis and modification
1.6	0.01	1.2	NMB0148	rpsC	30S ribosomal protein S3	5	a	Protein synthesis, Ribosomal proteins: synthesis and modification

1.7	0.011	1.3	NMB0149	rplP	50S ribosomal protein L16	5	a	Protein synthesis, Ribosomal proteins: synthesis and modification
1.6	0.013	1.2	NMB0150	rpmC	50S ribosomal protein L29	5	b	Protein synthesis, Ribosomal proteins: synthesis and modification
1.7	0.004	1	NMB0153	rplX	50S ribosomal protein L24	6	a	Protein synthesis, Ribosomal proteins: synthesis and modification
1.7	0.007	1.1	NMB0165	rpsM	30S ribosomal protein S13	6	b	Protein synthesis, Ribosomal proteins: synthesis and modification
1.5	0.014	1.1	NMB0167	rpsD	30S ribosomal protein S4	5	b	Protein synthesis, Ribosomal proteins: synthesis and modification
1.6	0.004	1.3	NMB0169	rplQ	50S ribosomal protein L17	6	a	Protein synthesis, Ribosomal proteins: synthesis and modification
1.7	0.011	0.7	NMB0722	rpmI	50S ribosomal protein L35	6	b	Protein synthesis, Ribosomal proteins: synthesis and modification
2.1	0.001	0.9	NMB0941	rpmJ	50S ribosomal protein L36	6	b	Protein synthesis, Ribosomal proteins: synthesis and modification
1.9	<0.001	0.9	NMB0942	rpmE	50S ribosomal protein L31	6	a	Protein synthesis, Ribosomal proteins: synthesis and modification
1.6	0.048	1.2	NMB0720	thrS	Threonyl-tRNA synthetase	3	b	Protein synthesis, tRNA aminoacylation
1.7	0.01	1.4	NMB0205	fur	Ferric uptake regulation protein	6	b	Regulatory functions, Other
1.6	0.011		NMB1249		Nitrate-nitrite sensory protein NarQ	3	a	Regulatory functions, Other
1.7	0.002	1	NMB1843	farR	Transcriptional regulator, MarR family	6	a	Regulatory functions, Other
2.7	<0.001	1.3	NMB1967		Transcriptional regulator, AraC family	3	a	Regulatory functions, Other
1.8	0.001	1.4	NMB0645		Ribonuclease	4	a	Transcription, Degradation of RNA
1.5	0.006	1.2	NMB0168	rpoA	DNA-directed RNA polymerase, alpha subunit	5	a	Transcription, DNA-dependent RNA polymerase
1.6	0.023		NMB0632	fbpC	Iron(III) ABC transporter, ATP-binding protein	3	a	Transport and binding proteins, Cations and iron carrying compounds
1.8	0.004	1.4	NMB0633	fbpB	Iron(III) ABC transporter, permease protein	4	a	Transport and binding proteins, Cations and iron carrying compounds
2.8	<0.001	1.2	NMB0634	fbpA	Iron(III) ABC transporter, periplasmic binding protein	6	a	Transport and binding proteins, Cations and iron carrying compounds
3	<0.001	1.3	NMB0752		Bacterioferritin-associated ferredoxin	4	a	Transport and binding proteins, Cations and iron carrying compounds
4.3	<0.001		NMB1540	lbpA	Lactoferrin-binding protein A	4	a	Transport and binding proteins, Cations and iron carrying compounds
1.9	<0.001	1.2	NMB1730	tonB	TonB protein	5	a	Transport and binding proteins, Cations and iron carrying compounds
1.6	0.036	1.3	NMB0624		Glycosyltransferase	3	a	Unknown function, General
2.1	0.001	1	NMB1409		FrpA/C-related protein	5	b	Unknown function, General
1.9	0.001	1.3	NMB2016		Type IV pilin-related protein	6	a	Unknown function, General
