# Supplementary material for: Host Iron Binding Proteins Acting as Niche Indicators for Neisseria meningitidis
Source: PLoS One. 2009 Apr 8;4(4):e5198. doi: 10.1371/journal.pone.0005198 (PMC2662411; doi:10.1371/journal.pone.0005198)
Supplement: Table S2 — Genes up-regulated in the presence of Transferrin compared to Haemoglobin. 1 Fold ratio is the relative transcript abundance in the presence of Transferrin compared to the presence of Haemoglobin. 2 The number of comparisons in which this gene was reliably detected. 3 A measure of the number of comparisons in which the gene was changed in the same direction. a-all one direction, b-one in opposite direction, c-two in opposite direction. (0.02 MB PDF) [file pone.0005198.s004.pdf]

Table S2: Genes up-regulated in the presence of Transferrin compared to Haemoglobin

Fold Ratio Tf/Hb[1]	CyberT *p*-value	Fold Ratio (Fe+/Fe-)	NMB Synonym	Gene	Gene Annotation	Assays[2]	Consistency[3]	TIGR family
1.7	0.008	0.9	NMB1839	fhs	Formate--tetrahydrofolate ligase	4	a	Amino acid biosynthesis, Aspartate family
2.2	<0.001	0.7	NMB1036	leuC	3-isopropylmalate dehydratase, large subunit	4	a	Amino acid biosynthesis, Pyruvate family
1.6	0.006	1	NMB1559	gshB	Glutathione synthetase	5	a	Biosynthesis of cofactors, prosthetic groups, and carriers, Glutathione and analogs
1.7	0.047	0.8	NMB1864	hemL	Glutamate-1-semialdehyde 2,1-aminomutase	3	a	Biosynthesis of cofactors, prosthetic groups, and carriers, Heme, porphyrin, and cobalamin
1.6	0.008	1	NMB1651	alr	Alanine racemase	4	a	Cell envelope, Biosynthesis and degradation of murein sacculus and peptidoglycan
1.7	0.018	1	NMB0285		O-antigen acetylase	4	a	Cell envelope, Biosynthesis and degradation of surface polysaccharides and lipopolysaccharides
1.5	0.011	0.8	NMB1898	mlp	Lipoprotein	5	b	Cell envelope, Other
1.6	0.003	1.1	NMB0992	hsf	Adhesin	4	a	Cell envelope, Surface structures
1.7	0.002	1.2	NMB0051		Twitching motility protein	5	a	Cellular processes, Chemotaxis and motility
1.5	0.021	1.1	NMB0280		Organic solvent tolerance protein	4	a	Cellular processes, Detoxification
1.7	0.004	0.7	NMB2091		Haemolysin	5	a	Cellular processes, Toxin production and resistance
1.6	0.004	0.9	NMB0494		DNA helicase	4	a	DNA metabolism, DNA replication, recombination, and repair
1.9	<0.001	1.3	NMB0740	recN	DNA repair protein RecN	6	a	DNA metabolism, DNA replication, recombination, and repair
1.8	0.023	0.9	NMB1261		Type III restriction system methylase	4	b	DNA metabolism, Restriction/modification
4.8	<0.001	1.1	NMB0546		Alcohol dehydrogenase, propanol preferring	4	a	Energy metabolism, Fermentation
2.3	<0.001	0.7	NMB0954	gltA	Citrate synthase	4	a	Energy metabolism, TCA cycle
2.6	0.006	0.8	NMB0957	lpd	2-oxoglutarate dehydrogenase, E3 component, lipoamide dehydrogenase	5	b	Energy metabolism, TCA cycle
2.2	0.001	0.7	NMB0213		Hypothetical protein	5	a	Hypothetical proteins
1.7	0.003	1.4	NMB0555		Hypothetical protein	5	a	Hypothetical proteins
2	0.019	0.9	NMB0570		Hypothetical protein	3	a	Hypothetical proteins
2.1	0.001	1.1	NMB0676		Hypothetical protein	4	a	Hypothetical proteins
1.6	0.009	1.5	NMB0793		Hypothetical protein	6	b	Hypothetical proteins
1.6	0.009	0.9	NMB0820		Hypothetical protein	4	a	Hypothetical proteins
1.6	<0.001	0.9	NMB1117		Hypothetical protein	5	a	Hypothetical proteins
1.5	0.028	1.5	NMB1426		Hypothetical protein	4	a	Hypothetical proteins

1.7	0.021	0.9	NMB1427		Hypothetical protein	4	b	Hypothetical proteins
2.2	<0.001	0.9	unannotated between NMB1000/01		Hypothetical protein	4	a	Hypothetical proteins
2.2	0.003	1.2	unannotated between NMB1000/01		Hypothetical protein	4	a	Hypothetical proteins
2.3	0.005	0.7	NMB0800		Conserved hypothetical protein	3	a	Hypothetical proteins, Conserved
2	0.001	1	NMB1436		Conserved hypothetical protein	5	a	Hypothetical proteins, Conserved
1.5	0.024		NMB0913	pemK	PemK protein	3	a	Mobile and extrachromosomal element functions, Plasmid functions
2	0.001	0.8	NMB0556		Repressor protein	5	a	Mobile and extrachromosomal element functions, Prophage functions
1.5	0.003	0.9	NMB0214	prlC	Oligopeptidase A	5	a	Protein fate, Degradation of proteins, peptides, and glycopeptides
1.9	0.004	0.8	NMB1832	lspA	Lipoprotein signal peptidase	4	a	Protein fate, Protein and peptide secretion and trafficking
1.6	0.008	0.9	NMB2056	rpsI	30S ribosomal protein S9	4	a	Protein synthesis, Ribosomal proteins: synthesis and modification
1.7	0.002	1	NMB0124, NMB0139	tufA	Translation elongation factor Tu	4	a	Protein synthesis, Translation factors
1.5	0.011	0.9	NMB1595	alaS	Alanyl-tRNA synthetase	6	b	Protein synthesis, tRNA aminoacylation
1.8	0.044	1.1	NMB0284	purB	Adenylosuccinate lyase	4	a	Purines, pyrimidines, nucleosides, and nucleotides, Purine ribonucleotide biosynthesis
2	<0.001	0.7	NMB0810		Transcriptional regulator, TetR family	4	a	Regulatory functions, Other
2.2	<0.001	0.7	NMB2039	porB	Major outer membrane protein PIB	5	a	Transport and binding proteins, Porins
1.8	0.016	1.4	NMB1299		Sodium dependent transporter	3	a	Transport and binding proteins, Unknown
1.6	0.014	0.8	NMB1516	fixS	FixS protein	3	a	Unknown function, General
