# Supplementary material for: Host Iron Binding Proteins Acting as Niche Indicators for Neisseria meningitidis
Source: PLoS One. 2009 Apr 8;4(4):e5198. doi: 10.1371/journal.pone.0005198 (PMC2662411; doi:10.1371/journal.pone.0005198)
Supplement: Table S3 — Genes up-regulated in the presence of Haemoglobin compared to Lactoferrin. 1 Fold ratio is the relative transcript abundance in the presence of Haemoglobin compared to the presence of Lactoferrin. 2 The number of comparisons in which this gene was reliably detected. 3 A measure of the number of comparisons in which the gene was changed in the same direction. a-all one direction, b-one in opposite direction, c-two in opposite direction. (0.02 MB PDF) [file pone.0005198.s005.pdf]

Table S3: Genes up-regulated in the presence of Haemoglobin compared to Lactoferrin

Fold Ratio Hb/Lf[1]	CyberT *p*-value	Fold Ratio (Fe-/Fe+)	NMB Synonym	Gene	Gene Annotation	Assays[2]	Consistency[3]	TIGR family
2	0.008		NMB2040	thiC	Thiamine biosynthesis protein ThiC	3	a	Biosynthesis of cofactors, prosthetic groups, and carriers, Thiamine
1.6	0.03		NMB0416	murF	UDP-N-acetylmuramoylalanyl-D-glutamyl-2,6-diaminopimelate--D-alanyl-D-alanyl ligase	4	b	Cell envelope, Biosynthesis and degradation of murein sacculus and peptidoglycan
1.6	0.009	1.3	NMB0345		Cell-binding factor	6	b	Cell envelope, Other
1.5	0.04	1	NMB0382	rmpM	Outer membrane protein class 4	4	a	Cell envelope, Other
2	0.007	1.1	NMB0946		Peroxiredoxin 2 family protein-glutaredoxin	6	b	Cell envelope, Other
1.8	0.032	1	NMB0547		Pilin	5	b	Cell envelope, Surface structures
2.3	0.01	1.3	NMB0712	rpoH	RNA polymerase sigma-32 factor	3	a	Cellular processes, Adaptations to atypical conditions
2.2	0.007	1.4	NMB1768		Haemagglutinin/haemolysin-related protein	6	b	Cellular processes, Toxin production and resistance
2.5	0.001	1.4	NMB0804		NAD(P)H nitroreductase	4	a	Central intermediary metabolism, Other
1.8	0.013	1.4	NMB1366		Thioredoxin	5	b	Energy metabolism, Electron transport
2	0.035	1.3	NMB1394	eda	4-hydroxy-2-oxoglutarate aldolase-2-deydro-3-deoxyphosphogluconate aldolase	4	b	Energy metabolism, Entner-Doudoroff
1.9	0.023	1.1	NMB1388	pgi-2	Glucose-6-phosphate isomerase	3	a	Energy metabolism, Glycolysis/gluconeogenesis
1.5	0.008	1.2	NMB0129		Hypothetical protein	6	a	Hypothetical proteins
1.5	0.016	1.4	NMB0755		Hypothetical protein	5	a	Hypothetical proteins
2.4	0.002	1.4	NMB0858		Hypothetical protein	5	b	Hypothetical proteins
2.5	0.004	1.1	NMB0899		Hypothetical protein	5	b	Hypothetical proteins
2.3	<0.001	0.9	NMB0945		Hypothetical protein	6	a	Hypothetical proteins
1.6	0.013	1	NMB1056		Hypothetical protein	6	a	Hypothetical proteins
1.5	0.028	1	NMB1455		Hypothetical protein	4	a	Hypothetical proteins
1.6	0.011	0.9	NMB1508		Hypothetical protein	4	a	Hypothetical proteins
2.2	<0.001	1.2	NMB1844		Hypothetical protein	6	a	Hypothetical proteins
1.5	0.019	1.1	NMB2013		Hypothetical protein	6	a	Hypothetical proteins
1.6	0.004	1.3	NMB2121		Hypothetical protein	6	a	Hypothetical proteins
3.1	0.01	0.9	NMB2130		Hypothetical protein	3	a	Hypothetical proteins
1.5	0.037	1.3	unannotated between NMB0863/64		Hypothetical protein	6	b	Hypothetical proteins
2	0.017	1	NMB1308		Conserved hypothetical protein	5	b	Hypothetical proteins, Conserved
4.6	<0.001	0.6	NMB1475		Conserved hypothetical protein	4	a	Hypothetical proteins, Conserved

2.4	0.04	1.2	NMB1557		Conserved hypothetical protein	3	b	Hypothetical proteins, Conserved
1.5	0.003	1.4	NMB0162	secY	Preprotein translocase SecY subunit	6	a	Protein fate, Protein and peptide secretion and trafficking
1.7	0.013	1.4	NMB0561	grpE	GrpE protein	4	a	Protein fate, Protein folding and stabilization
1.7	0.015	0.8	NMB1973	groES	Chaperonin, 10 kDa	4	a	Protein fate, Protein folding and stabilization
1.6	0.007	1.3	NMB0130	rplJ	50S ribosomal protein L10	6	a	Protein synthesis, Ribosomal proteins: synthesis and modification
2.2	0.012	0.8	NMB0145	rplB	50S ribosomal protein L2	3	a	Protein synthesis, Ribosomal proteins: synthesis and modification
1.9	0.005	1.5	NMB0146	rpsS	30S ribosomal protein S19	5	a	Protein synthesis, Ribosomal proteins: synthesis and modification
1.6	0.033	1.2	NMB0151	rpsQ	30S ribosomal protein S17	6	b	Protein synthesis, Ribosomal proteins: synthesis and modification
1.6	0.008	1.1	NMB0167	rpsD	30S ribosomal protein S4	6	b	Protein synthesis, Ribosomal proteins: synthesis and modification
2	0.013	1.3	NMB0169	rplQ	50S ribosomal protein L17	6	b	Protein synthesis, Ribosomal proteins: synthesis and modification
1.5	0.025	0.7	NMB0722	rpmI	50S ribosomal protein L35	5	a	Protein synthesis, Ribosomal proteins: synthesis and modification
4.9	<0.001	0.9	NMB0941	rpmJ	50S ribosomal protein L36	6	a	Protein synthesis, Ribosomal proteins: synthesis and modification
5.5	<0.001	0.9	NMB0942	rpmE	50S ribosomal protein L31	6	a	Protein synthesis, Ribosomal proteins: synthesis and modification
1.7	0.039	1.1	NMB2101	rpsB	30S ribosomal protein S2	5	b	Protein synthesis, Ribosomal proteins: synthesis and modification
1.6	0.032	0.8	NMB2102	tsf	Elongation factor TS	4	a	Protein synthesis, Translation factors
1.8	0.008	1.4	NMB0205	fur	Ferric uptake regulation protein	3	a	Regulatory functions, Other
1.5	0.04	1.2	NMB1007		Transcriptional regulator	4	a	Regulatory functions, Other
1.6	0.034		NMB1249		Nitrate-nitrite sensory protein NarQ	5	a	Regulatory functions, Other
2	0.012	1.4	NMB0633	fbpB	Iron(III) ABC transporter, permease protein	5	b	Transport and binding proteins, Cations and iron carrying compounds
2	0.016	1.2	NMB0634	fbpA	Iron(III) ABC transporter, periplasmic binding protein	4	b	Transport and binding proteins, Cations and iron carrying compounds
2.4	0.008	1.3	NMB0752		Bacterioferritin-associated ferredoxin	4	b	Transport and binding proteins, Cations and iron carrying compounds
1.7	0.026	1.2	NMB1730	tonB	TonB protein	6	b	Transport and binding proteins, Cations and iron carrying compounds
1.7	0.029	1	NMB1409		FrpA/C-related protein	5	b	Unknown function, General
1.7	0.003	1.3	NMB2016		Type IV pilin-related protein	6	a	Unknown function, General
