# Supplementary material for: Host Iron Binding Proteins Acting as Niche Indicators for Neisseria meningitidis
Source: PLoS One. 2009 Apr 8;4(4):e5198. doi: 10.1371/journal.pone.0005198 (PMC2662411; doi:10.1371/journal.pone.0005198)
Supplement: Table S4 — Genes up-regulated in the presence of Lactoferrin compared to Haemoglobin. 1 Fold ratio is the relative transcript abundance in the presence of Lactoferrin compared to the presence of Haemoglobin. 2 The number of comparisons in which this gene was reliably detected. 3 A measure of the number of comparisons in which the gene was changed in the same direction. a-all one direction, b-one in opposite direction, c-two in opposite direction. (0.03 MB PDF) [file pone.0005198.s006.pdf]

Table S4: Genes up-regulated in the presence of Lactoferrin compared to Haemoglobin

Fold Ratio Lf/Hb[1]	CyberT *p*-value	Fold Ratio (Fe+/Fe-)	NMB Synonym	Gene	Gene Annotation	Assays[2]	Consistency[3]	TIGR family
1.7	0.025	1	NMB2029	thrB	Homoserine kinase	5	b	Amino acid biosynthesis, Aspartate family
1.8	0.029	0.8	NMB2079	asd	Aspartate-semialdehyde dehydrogenase	5	a	Amino acid biosynthesis, Aspartate family
1.8	0.038		NMB0630	hisH	Amidotransferase HisH	4	a	Amino acid biosynthesis, Histidine family
2.0	0.026	0.8	NMB1864	hemL	Glutamate-1-semialdehyde 2,1-aminomutase	5	b	Biosynthesis of cofactors, prosthetic groups, and carriers, Heme, porphyrin, and cobalamin
1.7	0.014	0.8	NMB2030	ubiG	3-demethylubiquinone-9 3-methyltransferase	4	a	Biosynthesis of cofactors, prosthetic groups, and carriers, Menaquinone and ubiquinone
1.7	0.003	0.8	NMB1658	dfp	DNA/pantothenate metabolism flavoprotein	6	a	Biosynthesis of cofactors, prosthetic groups, and carriers, Pantothenate and coenzyme A
1.7	0.014	1	NMB1651	alr	Alanine racemase	5	b	Cell envelope, Biosynthesis and degradation of murein sacculus and peptidoglycan
1.5	0.013		NMB2032	lgtG	Lipopolysaccharide glycosyl transferase	3	a	Cell envelope, Biosynthesis and degradation of surface polysaccharides and lipopolysaccharides
1.6	0.023	0.8	NMB1898	mlp	Lipoprotein	4	a	Cell envelope, Other
1.6	0.012	1.1	NMB0992	hsf	Adhesin	6	b	Cell envelope, Surface structures
1.9	0.014	0.9	NMB1811	pilP	PilP protein	6	b	Cell envelope, Surface structures
1.9	0.016	0.8	NMB1987	thdF	Thiophene and furan oxidation protein ThdF	4	b	Cellular processes, Detoxification
1.7	0.003	0.8	NMB0468	speA	Biosynthetic arginine decarboxylase	6	a	Central intermediary metabolism: Polyamine biosynthesis
1.7	0.017	0.8	NMB1363	xseA	Exodeoxyribonuclease, large subunit	3	a	DNA metabolism, Degradation of DNA
1.8	0.001	0.7	NMB0399	xthA	Exodeoxyribonuclease III	6	a	DNA metabolism, DNA replication, recombination, and repair
2.3	0.001	1.3	NMB0740	recN	DNA repair protein RecN	6	a	DNA metabolism, DNA replication, recombination, and repair
1.5	0.017	0.9	NMB1903	dnaA	Chromosomal replication initiator protein DnaA	5	a	DNA metabolism, DNA replication, recombination, and repair
1.6	0.012	0.8	NMB1734	grx	Glutaredoxin	6	b	Energy metabolism, Electron transport
1.5	0.043	0.9	NMB2052	petB	Ubiquinol-cytochrome c reductase, cytochrome b	6	c	Energy metabolism, Electron transport
1.5	0.006	1	NMB2053	petA	Ubiquinol-cytochrome c reductase, iron-sulfur subunit	5	a	Energy metabolism, Electron transport
4.6	<0.001	1.1	NMB0546		Alcohol dehydrogenase, propanol preferring	6	a	Energy metabolism, Fermentation
2.1	0.004	0.7	NMB1604	gpm	Phosphoglycerate mutase	5	b	Energy metabolism, Glycolysis/gluconeogenesis
2.5	0.001	0.7	NMB2060	gpsA	Glycerol-3-phosphate dehydrogenase	5	a	Energy metabolism, Other

					(NAD\+)			
1.7	0.002	0.8	NMB0957	lpd	2-oxoglutarate dehydrogenase, E3 component, lipoamide dehydrogenase	5	a	Energy metabolism, TCA cycle
1.8	0.002	0.9	NMB0247		Hypothetical protein	5	b	Hypothetical proteins
1.7	0.003	1.1	NMB0676		Hypothetical protein	6	a	Hypothetical proteins
1.8	0.006	0.8	NMB0907		Hypothetical protein	5	b	Hypothetical proteins
2.7	<0.001	0.8	NMB1117		Hypothetical protein	6	a	Hypothetical proteins
1.6	0.012	0.7	NMB1221		Hypothetical protein	5	a	Hypothetical proteins
2.0	0.004	0.9	NMB2137		Hypothetical protein	5	a	Hypothetical proteins
1.5	0.015	0.7	unannotated between NMB0524/25		Hypothetical protein	5	a	Hypothetical proteins
3.0	<0.001	1.2	unannotated between NMB1000/01		Hypothetical protein	6	b	Hypothetical proteins
2.1	0.002	0.9	unannotated between NMB1000/01		Hypothetical protein	6	b	Hypothetical proteins
1.8	0.005	1.1	NMB0048, unannotated between NMB1541/42		Conserved hypothetical protein	5	a	Hypothetical proteins, Conserved
1.8	0.048	1	NMB0276		Conserved hypothetical protein	5	b	Hypothetical proteins, Conserved
1.6	0.03	0.8	NMB0410		Conserved hypothetical protein	6	b	Hypothetical proteins, Conserved
2.0	0.011	1.4	NMB0674		Conserved hypothetical protein	3	a	Hypothetical proteins, Conserved
1.8	0.039	0.7	NMB0800		Conserved hypothetical protein	5	b	Hypothetical proteins, Conserved
2.0	0.006	0.7	NMB1354		Conserved hypothetical protein	4	a	Hypothetical proteins, Conserved
1.8	0.015	1	NMB1436		Conserved hypothetical protein	5	b	Hypothetical proteins, Conserved
1.9	0.003	0.7	NMB1437		Conserved hypothetical protein	5	a	Hypothetical proteins, Conserved
1.9	0.01	1.4	NMB0914	pemI	PemI protein	4	a	Mobile and extrachromosomal element functions, Plasmid functions
2.1	0.003	0.8	NMB0556		Repressor protein	5	a	Mobile and extrachromosomal element functions, Prophage functions
1.6	0.014	0.7	NMB0896		Integrase - phage related	5	a	Mobile and extrachromosomal element functions, Prophage functions
1.8	<0.001	1	NMB1802	gcp	O-sialoglycoprotein endopeptidase	6	a	Protein fate, Degradation of proteins, peptides, and glycopeptides
1.7	0.049		NMB0622	lolA	Outer membrane lipoprotein carrier protein	3	a	Protein fate, Protein and peptide secretion and trafficking
2.2	0.004	0.6	NMB0550	dsbC	Thiol:disulfide interchange protein DsbC	5	b	Protein fate, Protein folding and stabilization
2.1	0.028	1	NMB1262	ppiA	Peptidyl-prolyl cis-trans isomerase	3	a	Protein fate, Protein folding and stabilization
1.7	0.009	0.8	NMB2056	rpsI	30S ribosomal protein S9	5	b	Protein synthesis, Ribosomal proteins: synthesis and modification
1.7	0.018	1	NMB0124,	tufA	Translation elongation factor Tu	4	b	Protein synthesis, Translation factors

			NMB0139					
1.8	0.008	0.7	NMB0814	hisS-1	Histidyl-tRNA synthetase	6	b	Protein synthesis, tRNA aminoacylation
1.6	0.025	0.9	NMB1595	alaS	Alanyl-tRNA synthetase	5	b	Protein synthesis, tRNA aminoacylation
1.8	0.002	0.7	NMB0810		Transcriptional regulator, TetR family	5	a	Regulatory functions, Other
1.7	0.002	0.7	NMB1049		Transcriptional regulator	6	a	Regulatory functions, Other
1.5	0.021	0.8	NMB1989		Iron(III) ABC transporter, periplasmic binding protein	5	b	Transport and binding proteins, Cations and iron carrying compounds
1.5	0.015		NMB0445		Bicyclomycin resistance protein	4	a	Transport and binding proteins, Other
2.1	0.008	0.7	NMB2039	porB	Major outer membrane protein PIB	6	b	Transport and binding proteins, Porins
1.9	0.002	1.4	NMB1299		Sodium dependent transporter	4	a	Transport and binding proteins, Unknown
1.6	0.007	0.7	NMB0041		ABC transporter, periplasmic solute-binding protein	5	a	Transport and binding proteins, Unknown substrate
1.5	0.044	0.9	NMB0374		MafB-related protein	5	b	Unknown function, General
1.6	0.006	1.1	NMB1753		VapD-related protein	5	a	Unknown function, General
1.8	0.011	0.8	NMB1909		Maf/YceF/YhdE family protein	5	a	Unknown function, General
2.0	0.026	1.3	NMB2041		Thiamin pyrophosphokinase-related protein	3	a	Unknown function, General
