# Supplementary material for: Host Iron Binding Proteins Acting as Niche Indicators for Neisseria meningitidis
Source: PLoS One. 2009 Apr 8;4(4):e5198. doi: 10.1371/journal.pone.0005198 (PMC2662411; doi:10.1371/journal.pone.0005198)
Supplement: Table S5 — Genes up-regulated in the presence of Transferrin compared to Lactoferrin. 1 Fold ratio is the relative transcript abundance in the presence of Transferrin compared to the presence of Lactoferrin. 2 The number of comparisons in which this gene was reliably detected. 3 A measure of the number of comparisons in which the gene was changed in the same direction. a-all one direction, b-one in opposite direction, c-two in opposite direction. (0.02 MB PDF) [file pone.0005198.s007.pdf]

Table S5: Genes up-regulated in the presence of Transferrin compared to Lactoferrin

Fold Ratio Tf/Lf [1]	CyberT *p*-value	Fold Ratio (Fe+/Fe-)	NMB Synonym	Gene	Gene Annotation	Assays[2]	Consistency[3]	TIGR family
2.1	<0.001	1.4	NMB1036	leuC	3-isopropylmalate dehydratase, large subunit	5	a	Amino acid biosynthesis, Pyruvate family
1.7	0.001	0.5	NMB0981	serB	Phosphoserine phosphatase	6	a	Amino acid biosynthesis, Serine family
1.5	0.033	0.7	NMB0712	rpoH	RNA polymerase sigma-32 factor	5	a	Cellular processes, Adaptations to atypical conditions
1.7	0.044	0.6	NMB0702		Competence protein ComA	3	b	Cellular processes, DNA transformation
1.6	0.027	1.1	NMB1032	nlaIVR	Type II restriction enzyme NlaIV	5	b	DNA metabolism, Restriction/modification
1.6	0.017	0.2	NMB0391	pgmB	Beta-phosphoglucomutase	4	a	Energy metabolism, Sugars
1.6	0.022	1.4	NMB0484, NMB0972, NMB1743		Hypothetical protein	5	b	Hypothetical proteins
2.1	0.034	1	NMB0730		Hypothetical protein	3	b	Hypothetical proteins
1.5	0.025	1.2	NMB0820		Hypothetical protein	5	a	Hypothetical proteins
1.5	0.007	1	NMB0928		Hypothetical protein	5	a	Hypothetical proteins
1.7	0.018	0.4	NMB0990, unannotated between NMB1086/87		Hypothetical protein	4	b	Hypothetical proteins
1.5	0.041	0.9	NMB1370		Hypothetical protein	6	c	Hypothetical proteins
1.7	0.032	0.3	NMB1693		Hypothetical protein	4	a	Hypothetical proteins
1.6	0.019	1.2	NMB2137		Hypothetical protein	6	c	Hypothetical proteins
1.5	0.026	0.7	NMB0343		Conserved hypothetical protein	4	b	Hypothetical proteins, Conserved
1.5	0.023	0.5	NMB1059		Conserved hypothetical protein	6	c	Hypothetical proteins, Conserved
1.5	0.013	0.7	NMB1062		Conserved hypothetical protein	4	a	Hypothetical proteins, Conserved
2	0.006	0.6	NMB1336		Conserved hypothetical protein	4	b	Hypothetical proteins, Conserved
1.6	0.014	0.6	NMB0700	iga	IgA-specific serine endopeptidase	6	b	Protein fate, Degradation of proteins, peptides, and glycopeptides
1.5	0.039	0.7	NMB0131	rplL	50S ribosomal protein L7-L12	5	b	Protein synthesis, Ribosomal proteins: synthesis and modification
5	<0.001	1.1	NMB0941	rpmJ	50S ribosomal protein L36	6	a	Protein synthesis, Ribosomal proteins: synthesis and modification
4.8	<0.001	1.1	NMB0942	rpmE	50S ribosomal protein L31	6	a	Protein synthesis, Ribosomal proteins: synthesis and modification
1.7	0.01	0.8	NMB1141, NMB1179		RNA methyltransferase, TrmH family	4	a	Protein synthesis, tRNA and rRNA base modification

2.1	0.013	0.8	NMB0638	galU	UTP--glucose-1-phosphate uridylyltransferase	3	a	Purines, pyrimidines, nucleosides, and nucleotides, Sugar-nucleotide biosynthesis and conversions
2.7	0.008	1.1	NMB0114		Nitrogen regulation protein NtrY	3	a	Regulatory functions, Other
2.1	0.003	1.1	NMB1017	sbp	Sulfate ABC transporter, periplasmic sulfate-binding protein	5	a	Transport and binding proteins, Anions
1.6	0.003	0.7	NMB1497		TonB-dependent receptor	6	a	Transport and binding proteins, Cations and iron carrying compounds
1.6	0.006	1	NMB0004		EpiH/GdmH-related protein	4	a	Unknown function, General
