# Supplementary material for: Host Iron Binding Proteins Acting as Niche Indicators for Neisseria meningitidis
Source: PLoS One. 2009 Apr 8;4(4):e5198. doi: 10.1371/journal.pone.0005198 (PMC2662411; doi:10.1371/journal.pone.0005198)
Supplement: Table S6 — Genes up-regulated in the presence of Lactoferrin compared to Transferrin. 1 Fold ratio is the relative transcript abundance in the presence of Lactoferrin compared to the presence of Transferrin. 2 The number of comparisons in which this gene was reliably detected. 3 A measure of the number of comparisons in which the gene was changed in the same direction. a-all one direction, b-one in opposite direction, c-two in opposite direction. (0.01 MB PDF) [file pone.0005198.s008.pdf]

# Table S6: Genes up-regulated in the presence of Lactoferrin compared to Transferrin

| Fold Ratio Lf/Tf [1] | CyberT *p*-value | Fold Ratio (Fe-/Fe+) | NMB Synonym | Gene | Gene Annotation | Assays[2] | Consistency[3] | TIGR family |
|---|---|---|---|---|---|---|---|---|
| 1.9 | 0.043 | 1 | NMB0678 | trpA | Tryptophan synthase, alpha subunit | 3 | b | Amino acid biosynthesis, Aromatic amino acid family |
| 1.5 | 0.012 | 1.3 | NMB0740 | recN | DNA repair protein RecN | 6 | b | DNA metabolism, DNA replication, recombination, and repair |
| 1.7 | 0.007 | 0.5 | NMB0208 | | Ferredoxin, 4Fe-4S bacterial type | 6 | b | Energy metabolism, Electron transport |
| 1.8 | <0.001 | 1.1 | NMB0546 | | Alcohol dehydrogenase, propanol preferring | 6 | a | Energy metabolism, Fermentation |
| 1.5 | 0.048 | 0.9 | NMB0393 | | Multidrug resistance protein | 5 | c | Transport and binding proteins, Other |
