# Supplementary material for: Host Iron Binding Proteins Acting as Niche Indicators for Neisseria meningitidis
Source: PLoS One. 2009 Apr 8;4(4):e5198. doi: 10.1371/journal.pone.0005198 (PMC2662411; doi:10.1371/journal.pone.0005198)
Supplement: Table S7 — Genes up-regulated in the presence of Haemoglobin compared to Ferric chloride. 1 Fold ratio is the relative transcript abundance in the presence of Haemoglobin compared to the presence of Ferric chloride. 2 The number of comparisons in which this gene was reliably detected. 3 A measure of the number of comparisons in which the gene was changed in the same direction. a-all one direction, b-one in opposite direction. (0.02 MB PDF) [file pone.0005198.s009.pdf]

Table S7: Genes up-regulated in the presence of Haemoglobin compared to Ferric chloride

Fold Ratio Hb/Fe(iii)[1]	CyberT *p*-value	Fold Ratio (Fe-/Fe+)	NMB Synonym	Gene	Gene Annotation	Assays[2]	Consistency[3]	TIGR Family
2.6	0.004	0.9	NMB1929	lgtA	Lacto-N-neotetraose biosynthesis glycosyl transferase	3	a	Cell envelope, Biosynthesis and degradation of surface polysaccharides and lipopolysaccharides
1.5	0.028	1.1	NMB1152, NMB1190	cysJ	Sulfite reductase (NADPH) flavoprotein, alpha component	3	a	Central intermediary metabolism, Sulfur metabolism
1.9	0.016		NMB1257		Site-specific DNA methylase	3	a	DNA metabolism, Restriction/modification
1.6	0.006	1.1	NMB1896	dpnC	Type II restriction enzyme DpnI	7	b	DNA metabolism, Restriction/modification
2	0.003	1	NMB1842		4-hydroxyphenylacetate 3-hydroxylase, small subunit	4	a	Energy metabolism, Amino acids and amines
1.6	0.01	1.1	NMB1407		FrpA-related protein	6	b	Hypothetical proteins
1.6	0.026	0.9	NMB0032		Hypothetical protein	5	a	Hypothetical proteins
1.8	0.003	1.4	NMB0120		Hypothetical protein	7	b	Hypothetical proteins
1.5	0.047	1.3	NMB0201		Hypothetical protein	5	b	Hypothetical proteins
1.6	0.028	1.3	NMB0511		Hypothetical protein	3	a	Hypothetical proteins
1.7	0.047	0.7	NMB1746		Hypothetical protein	5	b	Hypothetical proteins
2.1	0.002	1.2	NMB1844		Hypothetical protein	7	b	Hypothetical proteins
1.7	0.038	0.9	NMB2112		Hypothetical protein	3	a	Hypothetical proteins
1.7	0.032	1	NMB2115		Hypothetical protein	4	a	Hypothetical proteins
1.6	0.033	0.8	unannotated between NMB1676/77		Hypothetical protein	4	a	Hypothetical proteins
2.1	0.014		NMB0036		Conserved hypothetical protein	3	a	Hypothetical proteins, Conserved
1.7	0.02	1.4	NMB0248		Conserved hypothetical protein	3	a	Hypothetical proteins, Conserved
1.6	0.016	0.7	NMB0419		Conserved hypothetical protein	3	a	Hypothetical proteins, Conserved
1.7	0.009	1.1	NMB2153		Conserved hypothetical protein	7	b	Hypothetical proteins, Conserved
1.6	0.044	1.3	NMB1956	rpmE	50S ribosomal protein L31	4	a	Protein synthesis, Ribosomal proteins: synthesis and modification
1.6	0.016		NMB1905	rnpA	Ribonuclease P protein component	3	a	Transcription, RNA processing
1.5	0.038		NMB1315	uraA	Uracil permease	4	a	Transport and binding proteins, Nucleosides, purines and pyrimidines
1.7	0.049	1.1	NMB0496, NMB1780		Haemolysin activator-related protein	3	a	Unknown function, General
