# Supplementary material for: Host Iron Binding Proteins Acting as Niche Indicators for Neisseria meningitidis
Source: PLoS One. 2009 Apr 8;4(4):e5198. doi: 10.1371/journal.pone.0005198 (PMC2662411; doi:10.1371/journal.pone.0005198)
Supplement: Table S8 — Genes up-regulated in the presence of Ferric chloride compared to Haemoglobin. 1 Fold ratio is the relative transcript abundance in the presence of Ferric chloride compared to the presence of Haemoglobin. 2 The number of comparisons in which this gene was reliably detected. 3 A measure of the number of comparisons in which the gene was changed in the same direction. a-all one direction, b-one in opposite direction, c-two in opposite direction. (0.02 MB PDF) [file pone.0005198.s010.pdf]

Table S8: Genes up-regulated in the presence of Ferric chloride compared to Haemoglobin

Fold Ratio Fe(iii)/ Hb [1]	CyberT *p*-value	Fold Ratio (Fe-/Fe+)	NMB Synonym	Gene	Gene Annotation	Assays[2]	Consistency[3]	TIGR Family
1.9	0.001	1.1	NMB1074	argB	Acetylglutamate kinase	5	a	Amino acid biosynthesis, Glutamate family
2.1	0.006	1.4	NMB1036	leuC	3-isopropylmalate dehydratase, large subunit	4	a	Amino acid biosynthesis, Pyruvate family
1.8	0.004	1.2	NMB1577	ilvI	Acetolactate synthase III, large subunit	5	a	Amino acid biosynthesis, Pyruvate family
1.7	0.014	1.3	NMB1055	glyA	Serine hydroxymethyltransferase	7	c	Amino acid biosynthesis, Serine family
1.6	0.014	1.1	NMB0749		Penicillin-binding protein 4	5	b	Cell envelope, Biosynthesis and degradation of murein sacculus and peptidoglycan
2.2	0.003	1.2	NMB1898	mlp	Lipoprotein	3	a	Cell envelope, Other
1.6	0.026	1.1	NMB0018	pilE	Type IV pilin class II	6	b	Cell envelope, Surface structures
1.6	0.037	1.3	NMB0170	minC	Septum site-determining protein MinC	4	a	Cellular processes, Cell division
1.8	0.006	1.4	NMB0427	ftsZ	Cell division protein FtsZ	4	a	Cellular processes, Cell division
1.8	0.002	1	NMB0052	pilT-1	Twitching motility protein PilT-1	5	a	Cellular processes, Chemotaxis and motility
1.6	0.008	1.1	NMB0768	pilT-2	Twitching motility protein PilT-2	6	b	Cellular processes, Chemotaxis and motility
2	0.039	0.8	NMB0641	ppa	Inorganic pyrophosphatase	5	b	Central intermediary metabolism, Phosphorus compounds
1.9	0.007	1.2	NMB1938	atpF	ATP synthase F0, B subunit	4	b	Energy metabolism, ATP-proton motive force interconversion
1.9	0.019	1.2	NMB0243	nuoC	NADH dehydrogenase I, C subunit	5	b	Energy metabolism, Electron transport
1.6	0.048	1.3	NMB1677		Cytochrome c5	4	b	Energy metabolism, Electron transport
1.8	0.011	1.5	NMB2060	gpsA	Glycerol-3-phosphate dehydrogenase	6	b	Energy metabolism, Other
2	0.006	1.1	NMB0247		Hypothetical protein	4	a	Hypothetical proteins
2.3	0.004	1.2	NMB0383		Hypothetical protein	4	b	Hypothetical proteins
1.9	0.016	1.4	NMB0685		Hypothetical protein	4	b	Hypothetical proteins
1.6	0.022	1.2	NMB0819		Hypothetical protein	4	a	Hypothetical proteins
1.7	0.012	1.2	NMB0820		Hypothetical protein	4	a	Hypothetical proteins
1.5	0.013	1	NMB0841		Hypothetical protein	5	a	Hypothetical proteins
1.7	0.004	0.7	NMB0900		Hypothetical protein	6	b	Hypothetical proteins
1.8	0.005	1.2	NMB1117		Hypothetical protein	4	a	Hypothetical proteins
2.3	<0.001	1.4	NMB1221		Hypothetical protein	4	a	Hypothetical proteins
1.7	0.028		NMB1644		Hypothetical protein	4	b	Hypothetical proteins
1.5	0.031	1	NMB2059		Hypothetical protein	5	a	Hypothetical proteins
1.9	0.002	1.2	NMB2137		Hypothetical protein	5	a	Hypothetical proteins
2.1	0.009	0.9	unannotated between NMB1000/01		Hypothetical protein	7	c	Hypothetical proteins

2.3	0.013	0.9	NMB0048, unannotated between NMB1541/42		Conserved hypothetical protein	3	a	Hypothetical proteins, Conserved
1.5	0.043	0.6	NMB0786		Conserved hypothetical protein	4	a	Hypothetical proteins, Conserved
1.6	0.016	1	NMB0952		Conserved hypothetical protein	7	b	Hypothetical proteins, Conserved
1.6	0.036	0.8	NMB1039		Conserved hypothetical protein	5	b	Hypothetical proteins, Conserved
2	0.008	1	NMB1118		Conserved hypothetical protein	4	a	Hypothetical proteins, Conserved
1.7	0.025	1.1	NMB1218		Conserved hypothetical protein	4	b	Hypothetical proteins, Conserved
2.2	<0.001	1	NMB1436		Conserved hypothetical protein	6	a	Hypothetical proteins, Conserved
2.3	<0.001	1.4	NMB1500		Conserved hypothetical protein	5	a	Hypothetical proteins, Conserved
1.8	0.005	0.9	NMB1564		Conserved hypothetical protein	4	a	Hypothetical proteins, Conserved
1.9	0.035	1.4	NMB1840		Conserved hypothetical protein	4	b	Hypothetical proteins, Conserved
1.8	0.002	1.2	NMB1649	dsbB	Disulfide bond formation protein B	6	a	Protein fate, Protein modification and repair
1.6	0.016	1.2	NMB0136	rpsL	30S ribosomal protein S12	6	a	Protein synthesis, Ribosomal proteins: synthesis and modification
1.7	0.023	1	NMB0325	rplU	50S ribosomal protein L21	5	b	Protein synthesis, Ribosomal proteins: synthesis and modification
1.6	0.013	1.1	NMB0592	rpsP	30S ribosomal protein S16	5	a	Protein synthesis, Ribosomal proteins: synthesis and modification
1.7	0.009	1.2	NMB0723	rplT	50S ribosomal protein L20	4	a	Protein synthesis, Ribosomal proteins: synthesis and modification
2.1	<0.001	1.2	NMB2056	rpsI	30S ribosomal protein S9	6	a	Protein synthesis, Ribosomal proteins: synthesis and modification
2.1	0.011	1.4	NMB0814	hisS-1	Histidyl-tRNA synthetase	4	b	Protein synthesis, tRNA aminoacylation
1.8	0.05	1	NMB1291	nrdA	Ribonucleoside-diphosphate reductase, alpha subunit	5	b	Purines, pyrimidines, nucleosides, and nucleotides, 2'-Deoxyribonucleotide metabolism
1.9	0.019	1.3	NMB1307	ndk	Nucleoside diphosphate kinase	4	b	Purines, pyrimidines, nucleosides, and nucleotides, Nucleotide and nucleoside interconversions
1.7	0.006	1.4	NMB1049		frame shifted transcriptional regulator	6	a	Regulatory functions, Other
1.6	0.03	1.1	NMB1591	mtrA	Transcriptional regulator MtrA	4	a	Regulatory functions, Other
1.7	0.005	0.6	NMB1429	porA	Outer membrane protein PorA	6	a	Transport and binding proteins, Porins
1.8	0.002	1.4	NMB2039	porB	Major outer membrane protein PIB	6	a	Transport and binding proteins, Porins
1.7	0.041	1.3	NMB1516	fixS	FixS protein	5	b	Unknown function, General
1.8	0.014	1.1	NMB2104		MafA protein	5	b	Unknown function, General
1.5	0.024	1.2	NMB2105	mafB	MafB protein	6	a	Unknown function, General
