# Supplementary material for: Host Iron Binding Proteins Acting as Niche Indicators for Neisseria meningitidis
Source: PLoS One. 2009 Apr 8;4(4):e5198. doi: 10.1371/journal.pone.0005198 (PMC2662411; doi:10.1371/journal.pone.0005198)
Supplement: Table S9 — Genes up-regulated in the presence of Haemoglobin compared to Haemoglobin and Transferrin. 1 Fold ratio is the relative transcript abundance in the presence of Haemoglobin compared to the presence of Haemoglobin and Transferrin. 2 Fold ratio is the relative transcript abundance in the presence of Haemoglobin compared to the presence of Transferrin. 3 The number of comparisons in which this gene was reliably detected. 4 A measure of the number of comparisons in which the gene was changed in the same direction. a-all one direction, b-one in opposite direction, c-two in opposite direction. (0.02 MB PDF) [file pone.0005198.s011.pdf]

Table S9: Genes up-regulated in the presence of Haemoglobin compared to Haemoglobin and Transferrin

Fold Ratio Hb/Hb +Tf [1]	CyberT *p*-value	Fold Ratio Hb/Tf [2]	Fold Ratio (Fe-/Fe+)	NMB Synonym	Gene	Gene Annotation	Assays[3]	Consistency[4]	TIGR Family
1.8	0.016	0.4	0.7	NMB1797		Penicillin-binding protein 3	3	a	Cell envelope, Biosynthesis and degradation of murein sacculus and peptidoglycan
1.6	0.027	1.3		NMB1273		Alginate O-acetylation protein AlgI	3	a	Cell envelope, Other
1.5	0.023	1.8	1.1	NMB1484	surE	Stationary-phase survival protein SurE	4	a	Cellular processes, Adaptations to atypical conditions
1.6	0.027	1.0	1.0	NMB1567		Macrophage infectivity potentiator	3	a	Cellular processes, Pathogenesis
3.4	<0.001	1.2	3.2	NMB1857	mdaB	Modulator of drug activity B	5	b	Cellular processes, Toxin production and resistance
1.6	0.046	2.3	1.4	NMB1768		Haemagglutinin/haemolysin-related protein	6	a	Cellular processes, Toxin production and resistance
1.8	0.005	1.0	1.3	NMB1451	dnaQ-1	DNA polymerase III, epsilon subunit	4	a	DNA metabolism, DNA replication, recombination, and repair
1.8	0.026	0.6	0.7	NMB0399	xthA	Exodeoxyribonuclease III	3	a	DNA metabolism, DNA replication, recombination, and repair
2.1	0.048	1.4	1.2	NMB0530		Glycosyl hydrolase, family 3	5	b	Energy metabolism, Biosynthesis and degradation of polysaccharides
2.5	0.007	1.1	1.2	NMB0503		Hypothetical protein	5	a	Hypothetical proteins
2.2	0.007	2.7	2.9	NMB1107		Hypothetical protein	4	a	Hypothetical proteins
2.0	0.012	0.6		NMB1002		Hypothetical protein	3	a	Hypothetical proteins
2.0	0.017			NMB1502		Hypothetical protein	4	a	Hypothetical proteins
1.9	0.024	0.9	1.0	NMB1456		Hypothetical protein	3	a	Hypothetical proteins
1.8	0.003	1.0		NMB0504, unannotated between NMB1776/77		Hypothetical protein	5	a	Hypothetical proteins
1.8	0.032	1.3	1.6	NMB0969, NMB1769		IS1016C2 transposase	3	a	Hypothetical proteins
1.7	0.020	1.3		NMB1224		Hypothetical protein	4	a	Hypothetical proteins
1.7	0.047	0.7	0.6	NMB1503		Hypothetical protein	6	c	Hypothetical proteins
1.6	0.043	1.0	1.3	NMB0847		Hypothetical protein	6	b	Hypothetical proteins
1.6	0.016	1.2		NMB1215		Hypothetical protein	6	a	Hypothetical proteins
1.6	0.027	1.0	1.2	NMB0501, NMB1773		Hypothetical protein	6	b	Hypothetical proteins
1.6	0.011	1.4	1.4	NMB0508		Hypothetical protein	6	b	Hypothetical proteins
1.5	0.032	1.0	0.9	NMB0908		Hypothetical protein	6	b	Hypothetical proteins
2.1	0.027	1.0	1.1	NMB1464		Conserved hypothetical protein	3	a	Hypothetical proteins, Conserved

1.7	0.011			NMB0529		Conserved hypothetical protein	6	a	Hypothetical proteins, Conserved
1.5	0.044	0.7	0.6	NMB1438		Conserved hypothetical protein	3	a	Hypothetical proteins, Conserved
1.5	0.021	0.9	1.3	NMB0919, NMB0991, NMB1000, NMB1054, NMB1399, NMB1539, NMB1601, NMB1984, NMB2043		IS1106 transposase	6	b	Mobile and extrachromosomal element functions, Transposon functions
1.9	0.006			NMB1471	trpS	Tryptophanyl-tRNA synthetase	3	a	Protein synthesis, tRNA aminoacylation
2.1	0.021	1.5	1.8	NMB1538	rpoD	RNA polymerase sigma factor RpoD	3	a	Transcription, Transcription factors
1.6	0.007		2.0	NMB0611		Polyamine permease inner membrane protein	6	a	Transport and binding proteins, Amino acids, peptides and amines
3.0	0.013	0.8	1.6	NMB1429	porA	Outer membrane protein PorA	3	a	Transport and binding proteins, Porins
