# Supplementary material for: Host Iron Binding Proteins Acting as Niche Indicators for Neisseria meningitidis
Source: PLoS One. 2009 Apr 8;4(4):e5198. doi: 10.1371/journal.pone.0005198 (PMC2662411; doi:10.1371/journal.pone.0005198)
Supplement: Table S10 — Genes up-regulated in the presence of Haemoglobin and Transferrin compared to Haemoglobin. 1 Fold ratio is the relative transcript abundance in the presence of Haemoglobin and Transferrin compared to the presence of Haemoglobin. 2 Fold ratio is the relative transcript abundance in the presence of Transferrin compared to the presence of Haemoglobin. 3 The number of comparisons in which this gene was reliably detected. 4 A measure of the number of comparisons in which the gene was changed in the same direction. a-all one direction, b-one in opposite direction, c-two in opposite direction. (0.03 MB PDF) [file pone.0005198.s012.pdf]

Table S10: Genes up-regulated in the presence of Haemoglobin and Transferrin compared to Haemoglobin

Fold Ratio Hb+Tf /Hb[1]	CyberT *p*-value	Fold Ratio Tf/Hb[2]	Fold Ratio (Fe-/Fe+)	NMB Synonym	Gene	Gene Annotation	Assays[3]	Consistency[4]	TIGR Family
1.8	0.028	1.1		NMB1876	argA	N-acetylglutamate synthase	5	b	Amino acid biosynthesis, Glutamate family
2.0	0.029	0.9	0.8	NMB1037	gshA	Glutamate--cysteine ligase	4	b	Biosynthesis of cofactors, prosthetic groups, and carriers, Glutathione and analogs
2.2	0.003		3.2	NMB2068		D-amino acid oxidase flavoprotein	3	a	Biosynthesis of cofactors, prosthetic groups, and carriers, Thiamine
1.7	0.036	0.9	1.0	NMB1949		Soluble lytic murein transglycosylase	4	a	Cell envelope, Biosynthesis and degradation of surface polysaccharides and lipopolysaccharides
1.9	0.012	0.9	0.7	NMB1946		Outer membrane lipoprotein	6	c	Cell envelope, Other
1.7	0.010	1.5	2.2	NMB1398	sodC	Cu-Zn-superoxide dismutase	3	a	Cellular processes, Detoxification
1.6	0.016		0.7	NMB0525		Aluminum resistance protein	5	a	Cellular processes, Detoxification
1.6	0.021		0.9	NMB0116	dprA	DNA processing chain A	6	a	Cellular processes, DNA transformation
1.6	0.021	2.5	1.8	NMB0585		Hypothetical protein	5	a	Cellular processes, Pathogenesis
3.1	0.018	1.8		NMB1001		Bacteriophage integrase	3	a	Disrupted reading frame
1.8	0.020	0.8	1.0	NMB1722		Cytochrome C555	4	a	Disrupted reading frame
2.1	0.004	0.9	0.9	NMB1935	atpG	ATP synthase F1, gamma subunit	5	a	Energy metabolism, ATP-proton motive force interconversion
1.7	0.049	0.8	0.5	NMB1725	fixN	Cytochrome c oxidase, subunit I	4	b	Energy metabolism, Electron transport
2.2	0.003	0.4	1.7	NMB0430		Carboxyphosphonoenolpyruvate phosphonomutase	6	b	Energy metabolism, Fermentation
1.9	0.018	0.7		NMB0997	dld	D-lactate dehydrogenase	6	b	Energy metabolism, Glycolysis/gluconeogenesis
1.6	0.009	1.1	1.1	NMB1388	pgi-2	Glucose-6-phosphate isomerase	4	a	Energy metabolism, Glycolysis/gluconeogenesis
2.2	0.026	0.4	1.4	NMB0948	sdhC	Succinate dehydrogenase, cytochrome b556 subunit	6	b	Energy metabolism, TCA cycle
2.0	0.046	0.8	0.8	NMB0433	acnA	Aconitate hydratase 1	4	b	Energy metabolism, TCA cycle
2.5	<0.001	1.7	0.9	NMB1211		Hypothetical protein	3	a	Hypothetical proteins
2.0	0.047	0.6	1.0	NMB0057, NMB0299, NMB1657, NMB2017		ComEA-related protein	6	c	Hypothetical proteins

1.9	0.018	2.1	3.0	NMB1598		Hypothetical protein	4	a	Hypothetical proteins
1.9	0.025	0.7	0.9	NMB0247		Hypothetical protein	6	c	Hypothetical proteins
1.9	0.013	1.3	0.8	NMB1941		Hypothetical protein	3	a	Hypothetical proteins
1.8	0.047	0.9	1.7	NMB1124, NMB1162		Hypothetical protein	3	b	Hypothetical proteins
1.8	0.002	1.3	1.0	NMB0818		Hypothetical protein	5	a	Hypothetical proteins
1.8	0.015	1.0	1.6	NMB0338		Hypothetical protein	3	a	Hypothetical proteins
1.8	0.016	1.6	0.8	NMB0429		Hypothetical protein	6	b	Hypothetical proteins
1.7	0.019	1.6		unannotated between NMB0820/21		Hypothetical protein	3	a	Hypothetical proteins
1.7	0.018	0.9	1.5	NMB1675		Hypothetical protein	5	b	Hypothetical proteins
1.7	0.015	1.5		NMB2080		Hypothetical protein	5	a	Hypothetical proteins
1.7	0.022	3.4	1.6	NMB0856		Hypothetical protein	4	a	Hypothetical proteins
1.7	0.009	1.9	1.4	NMB0755		Hypothetical protein	4	a	Hypothetical proteins
1.7	0.041	0.7	1.1	NMB1440		Hypothetical protein	4	b	Hypothetical proteins
1.6	0.010	1.1	1.1	NMB1397		Hypothetical protein	5	a	Hypothetical proteins
1.6	0.043	1.7	1.5	NMB0311		Hypothetical protein	4	b	Hypothetical proteins
1.6	0.032	1.3	1.1	NMB1212		Hypothetical protein	4	a	Hypothetical proteins
1.5	0.042	3.2		NMB2131		Hypothetical protein	4	a	Hypothetical proteins
2.3	0.008			NMB0830		Conserved hypothetical protein	5	b	Hypothetical proteins, Conserved
2.2	0.010	1.1	0.8	NMB1653		Conserved hypothetical protein	4	b	Hypothetical proteins, Conserved
1.9	<0.001	0.8	1.0	NMB1727		Conserved hypothetical protein	6	a	Hypothetical proteins, Conserved
1.9	0.009	1.3	1.3	NMB0773		Conserved hypothetical protein	3	a	Hypothetical proteins, Conserved
1.8	0.018	1.8	2.1	NMB0361		Conserved hypothetical protein	3	a	Hypothetical proteins, Conserved
1.8	0.024	1.2	1.2	NMB0406		Conserved hypothetical protein	4	a	Hypothetical proteins, Conserved
1.7	0.049	0.8		NMB0840		Conserved hypothetical protein	3	a	Hypothetical proteins, Conserved
1.6	0.040	1.5	1.8	NMB2078		Conserved hypothetical protein	4	b	Hypothetical proteins, Conserved
1.6	0.042	0.7	0.8	NMB0043		Conserved hypothetical protein	4	a	Hypothetical proteins, Conserved
1.5	0.018	2.5	1.3	NMB0317		Conserved hypothetical protein	6	b	Hypothetical proteins, Conserved
1.5	0.019			NMB1137, NMB1175		Conserved hypothetical protein	4	a	Hypothetical proteins, Conserved
2.1	0.030	1.1	1.2	NMB1386, NMB1736		Transposase	5	b	Mobile and extrachromosomal element functions, Transposon functions
3.4	<0.001	1.4	1.2	NMB0463	rpsT	30S ribosomal protein S20	3	a	Protein synthesis, Ribosomal proteins
2.4	0.031	1.4	1.2	NMB0321	rpmB	50S ribosomal protein L28	5	b	Protein synthesis, Ribosomal proteins
2.1	0.006	1.2	0.9	NMB0155	rpsN	30S ribosomal protein S14	4	a	Protein synthesis, Ribosomal proteins
2.1	0.004	1.6	1.5	NMB1911	rpmF	50S ribosomal protein L32	3	a	Protein synthesis, Ribosomal proteins

2.0	0.013	1.3	1.0	NMB1950	rpsU	30S ribosomal protein S21	3	a	Protein synthesis, Ribosomal proteins
2.0	0.003	1.3	1.1	NMB0156	rpsH	30S ribosomal protein S8	4	a	Protein synthesis, Ribosomal proteins
1.8	0.010	1.4	1.3	NMB1956	rpmE	50S ribosomal protein L31	6	b	Protein synthesis, Ribosomal proteins
1.7	0.047	1.9	1.6	NMB0147	rplV	50S ribosomal protein L22	6	b	Protein synthesis, Ribosomal proteins
1.7	0.050	0.8	0.9	NMB1320	rplI	50S ribosomal protein L9	5	b	Protein synthesis, Ribosomal proteins
1.6	0.011	1.6	1.3	NMB0130	rplJ	50S ribosomal protein L10	6	a	Protein synthesis, Ribosomal proteins
1.8	0.016	0.5		NMB1686	prfA	Peptide chain release factor 1	4	a	Protein synthesis, Translation factors
2.0	0.022	1.1	2.6	NMB2083	cysS	Cysteinyl-tRNA synthetase	4	b	Protein synthesis, tRNA aminoacylation
1.7	0.037	0.7	1.1	NMB0111	fmt	Methionyl-tRNA formyltransferase	4	b	Protein synthesis, tRNA aminoacylation
1.5	0.026	1.1	0.9	NMB1425	lysU	Lysyl-tRNA synthetase, heat inducible	4	a	Protein synthesis, tRNA aminoacylation
1.8	0.028	0.9	1.9	NMB1679	trmA	tRNA (uracil-5-)-methyltransferase	5	b	Protein synthesis, tRNA and rRNA base modification
1.5	0.037	0.8	0.8	NMB2047		Hypoxanthine-guanine phosphoribosyltransferase	5	b	Purines, pyrimidines, nucleosides, and nucleotides, Salvage of nucleosides and nucleotides
2.3	0.003	2.3	2.3	NMB0192	rnhB	Ribonuclease HII	4	a	Transcription, Degradation of RNA
2.2	0.023	0.8	0.7	NMB1200	vacB	Ribonuclease II family protein	3	a	Transcription, Degradation of RNA
2.1	0.007	0.9	1.2	NMB0402	putP	Sodium/proline symporter	4	a	Transport and binding proteins, Amino acids, peptides and amines
2.7	0.027	7.2	8.2	NMB0460	tbp2	Transferrin-binding protein 2	3	b	Transport and binding proteins, Cations and iron carrying compounds
1.9	0.048	1.3	1.3	NMB1271		Mercury transport periplasmic protein	3	a	Transport and binding proteins, Cations and iron carrying compounds
1.9	0.006	3.0	1.3	NMB0752		Bacterioferritin-associated ferredoxin	4	a	Transport and binding proteins, Cations and iron carrying compounds
2.1	0.023	1.2	1.3	NMB1240		ABC transporter, ATP-binding protein	4	b	Transport and binding proteins, Unknown substrate
1.5	0.032	0.8	0.7	NMB1975		Sodium- and chloride-dependent transporter	4	a	Transport and binding proteins, Unknown substrate
2.8	0.009	1.3	1.2	NMB1638		YhbX-YhjW-YijP-YjdB family protein	5	b	Unknown function, General
2.1	0.002	0.8		NMB0193	gidA	Glucose inhibited division protein A	4	a	Unknown function, General

1.8	0.044	0.8	1.0	NMB0004		EpiH/GdmH-related protein	5	c	Unknown function, General
1.8	0.015	1.1	1.2	NMB2063		SlyX protein, putative	4	a	Unknown function, General
1.7	0.043	2.8		NMB1669		Iron-starvation protein PigA	3	a	Unknown function, General
