# Supplementary material for: Host Iron Binding Proteins Acting as Niche Indicators for Neisseria meningitidis
Source: PLoS One. 2009 Apr 8;4(4):e5198. doi: 10.1371/journal.pone.0005198 (PMC2662411; doi:10.1371/journal.pone.0005198)
Supplement: Table S11 — Genes up-regulated by Haemoglobin (0.02 MB PDF) [file pone.0005198.s013.pdf]

Table S11: Genes up-regulated by Haemoglobin

Fold Ratio Hb/Tf	Fold Ratio Hb/Lf	Fold Ratio (Fe-/Fe+)	NMB Synonym	Gene	Gene Annotation	TIGR family
1.6	1.7	1	NMB0745	folQ	2-amino-4-hydroxy-6-hydroxymethyldihydropteridine-pyrophosphokinase	Biosynthesis of cofactors, prosthetic groups, and carriers, Folic acid
1.5	1.6	1.3	NMB0345		Cell-binding factor	Cell envelope, Other
1.7	2	1.1	NMB0946		Peroxiredoxin 2 family protein-glutaredoxin	Cell envelope, Other
2	1.7		NMB0799	ftsJ	Cell division protein FtsJ	Cellular processes, Cell division
2.3	2.2	1.4	NMB1768		Haemagglutinin/haemolysin-related protein	Cellular processes, Toxin production and resistance
1.8	1.8	1	NMB1366		Thioredoxin	Energy metabolism, Electron transport
1.7	1.5	1.2	NMB0129		Hypothetical protein	Hypothetical proteins
3.9	1.6	0.7	NMB0730		Hypothetical protein	Hypothetical proteins
2.8	2.4	1.4	NMB0858		Hypothetical protein	Hypothetical proteins
2	2.5	1.1	NMB0899		Hypothetical protein	Hypothetical proteins
1.7	2.3	0.9	NMB0945		Hypothetical protein	Hypothetical proteins
1.6	1.6	1	NMB1006		Hypothetical protein	Hypothetical proteins
1.7	1.5	1.2	NMB1008		Hypothetical protein	Hypothetical proteins
1.5	1.6	1	NMB1056		Hypothetical protein	Hypothetical proteins
1.7	1.8	0.9	NMB1211		Hypothetical protein	Hypothetical proteins
2.1	2.2	1.2	NMB1844		Hypothetical protein	Hypothetical proteins
1.6	1.5	1.1	NMB2013		Hypothetical protein	Hypothetical proteins
1.8	1.7	1.4	NMB0561	grpE	GrpE protein	Protein fate, Protein folding and stabilization
1.8	2	1.2	NMB0791		Peptidyl-prolyl cis-trans isomerase	Protein fate, Protein folding and stabilization
1.6	1.7	0.8	NMB1973	groES	Chaperonin, 10 kDa	Protein fate, Protein folding and stabilization
1.6	1.6	1.3	NMB0130	rplJ	50S ribosomal protein L10	Protein synthesis, Ribosomal proteins: synthesis and modification
1.7	1.6	1.4	NMB0131	rplL	50S ribosomal protein L7-L12	Protein synthesis, Ribosomal proteins: synthesis and modification
1.5	1.6	1.1	NMB0167	rpsD	30S ribosomal protein S4	Protein synthesis, Ribosomal proteins: synthesis and modification
1.6	2	1.3	NMB0169	rplQ	50S ribosomal protein L17	Protein synthesis, Ribosomal proteins: synthesis and modification
1.7	1.5	1.2	NMB0722	rpmI	50S ribosomal protein L35	Protein synthesis, Ribosomal proteins: synthesis and modification
2.1	4.9	0.9	NMB0941	rpmJ	50S ribosomal protein L36	Protein synthesis, Ribosomal proteins: synthesis and modification
1.9	5.5	0.9	NMB0942	rpmE	50S ribosomal protein L31	Protein synthesis, Ribosomal proteins: synthesis and modification
1.7	1.8	1.4	NMB0205	fur	Ferric uptake regulation protein	Regulatory functions, Other
1.6	1.6		NMB1249		Nitrate-nitrite sensory protein NarQ	Regulatory functions, Other

1.6	1.6		NMB0632	fbpC	Iron(III) ABC transporter, ATP-binding protein	Transport and binding proteins, Cations and iron carrying compounds
1.8	2	1.4	NMB0633	fbpB	Iron(III) ABC transporter, permease protein	Transport and binding proteins, Cations and iron carrying compounds
2.8	2	1.2	NMB0634	fbpA	Iron(III) ABC transporter, periplasmic binding protein	Transport and binding proteins, Cations and iron carrying compounds
3	2.4	1.3	NMB0752		Bacterioferritin-associated ferredoxin	Transport and binding proteins, Cations and iron carrying compounds
1.9	1.7	1.2	NMB1730	tonB	TonB protein	Transport and binding proteins, Cations and iron carrying compounds
2.1	1.7	1	NMB1409		FrpA/C-related protein	Unknown function, General
1.9	1.7	1.3	NMB2016		Type IV pilin-related protein	Unknown function, General
