# Supplementary material for: Host Iron Binding Proteins Acting as Niche Indicators for Neisseria meningitidis
Source: PLoS One. 2009 Apr 8;4(4):e5198. doi: 10.1371/journal.pone.0005198 (PMC2662411; doi:10.1371/journal.pone.0005198)
Supplement: Table S12 — Genes down-regulated by Haemoglobin (0.02 MB PDF) [file pone.0005198.s014.pdf]

Table S12: Genes down-regulated by Haemoglobin

Fold Ratio Tf /Hb	Fold Ratio Lf/Hb	Fold Ratio (Fe+/Fe-)	NMB Synonym	Gene	Gene Annotation	TIGR family
1.7	2	1.2	NMB1864	hemL	Glutamate-1-semialdehyde 2,1-aminomutase	Biosynthesis of cofactors, prosthetic groups, and carriers, Heme, porphyrin, and cobalamin
1.6	1.7	1.3	NMB2030	ubiG	3-demethylubiquinone-9 3-methyltransferase	Biosynthesis of cofactors, prosthetic groups, and carriers, Menaquinone and ubiquinone
1.5	1.5		NMB0423	murC	UDP-N-acetylmuramate-alanine ligase	Cell envelope, Biosynthesis and degradation of murein sacculus and peptidoglycan
1.6	1.7	1	NMB1651	alr	Alanine racemase	Cell envelope, Biosynthesis and degradation of murein sacculus and peptidoglycan
1.5	1.6	1.2	NMB1898	mlp	Lipoprotein	Cell envelope, Other
1.6	1.6	0.9	NMB0992	hsf	Adhesin	Cell envelope, Surface structures
1.5	1.6	0.9	NMB0280		Organic solvent tolerance protein	Cellular processes, Detoxification
1.7	1.8	1.4	NMB0399	xthA	Exodeoxyribonuclease III	DNA metabolism, DNA replication, recombination, and repair
1.9	2.3	0.8	NMB0740	recN	DNA repair protein RecN	DNA metabolism, DNA replication, recombination, and repair
1.8	2.2	1.2	NMB1938	atpF	ATP synthase F0, B subunit	Energy metabolism, ATP-proton motive force interconversion
4.8	4.6	0.9	NMB0546		Alcohol dehydrogenase, propanol preferring	Energy metabolism, Fermentation
1.9	1.7	1.3	NMB0334	pgi-1	Glucose-6-phosphate isomerase	Energy metabolism, Glycolysis/gluconeogenesis
2.5	2.3	1.5	NMB2060	gpsA	Glycerol-3-phosphate dehydrogenase (NAD\+)	Energy metabolism, Other
2.6	1.7	1.3	NMB0957	lpd	2-oxoglutarate dehydrogenase, E3 component, lipoamide dehydrogenase	Energy metabolism, TCA cycle
1.6	1.6	0.6	NMB1916	fabH	3-oxoacyl-(acyl-carrier-protein) synthase III	Fatty acid and phospholipid metabolism, Biosynthesis
2.1	1.7	0.9	NMB0676		Hypothetical protein	Hypothetical proteins
1.6	2.2	1.1	NMB1117		Hypothetical protein	Hypothetical proteins
1.6	1.6	1.4	NMB1221		Hypothetical protein	Hypothetical proteins
2.2	2.1	1.1	unannotated between NMB1000/01		Hypothetical protein	Hypothetical proteins
2.2	3	0.9	unannotated between NMB1000/01		Hypothetical protein	Hypothetical proteins
1.9	1.7	0.6	NMB0786		Conserved hypothetical protein	Hypothetical proteins, Conserved
2.3	1.8	1.4	NMB0800		Conserved hypothetical protein	Hypothetical proteins, Conserved
1.6	2	1.3	NMB1354		Conserved hypothetical protein	Hypothetical proteins, Conserved
2	1.8	1	NMB1436		Conserved hypothetical protein	Hypothetical proteins, Conserved
1.9	2	1.4	NMB1437		Conserved hypothetical protein	Hypothetical proteins, Conserved

2	2.1	1.2	NMB0556		Repressor protein	Mobile and extrachromosomal element functions, Prophage functions
1.7	1.6	1.3	NMB0896		Integrase - phage related	Mobile and extrachromosomal element functions, Prophage functions
1.5	1.6	1.2	NMB0214	prlC	Oligopeptidase A	Protein fate, Degradation of proteins, peptides, and glycopeptides
1.7	1.7		NMB0622	lolA	Outer membrane lipoprotein carrier protein	Protein fate, Protein and peptide secretion and trafficking
1.6	2.3		NMB2056	rpsI	30S ribosomal protein S9	Protein synthesis, Ribosomal proteins: synthesis and modification
1.7	1.7	1	NMB0124, NMB0139	tufA	Translation elongation factor Tu	Protein synthesis, Translation factors
1.6	1.8	1.4	NMB0814	hisS-1	Histidyl-tRNA synthetase	Protein synthesis, tRNA aminoacylation
1.5	1.6	1.1	NMB1595	alaS	Alanyl-tRNA synthetase	Protein synthesis, tRNA aminoacylation
1.9	1.7	0.6	NMB0290		Transcriptional regulator	Regulatory functions, Other
2	1.8		NMB0810		Transcriptional regulator, TetR family	Regulatory functions, Other
1.6	2	1.2	NMB2133		Sodium/dicarboxylate symporter family protein	Transport and binding proteins, Carbohydrates, organic alcohols, and acids
2.2	2.1	1.4	NMB2039	porB	Major outer membrane protein PIB	Transport and binding proteins, Porins
1.8	1.9	0.7	NMB1299		Sodium dependent transporter	Transport and binding proteins, Unknown
1.5	1.8	1.3	NMB1909		Maf/YceF/YhdE family protein	Unknown function, General
