# Supplementary material for: Host Iron Binding Proteins Acting as Niche Indicators for Neisseria meningitidis
Source: PLoS One. 2009 Apr 8;4(4):e5198. doi: 10.1371/journal.pone.0005198 (PMC2662411; doi:10.1371/journal.pone.0005198)
Supplement: Table S13 — Genes up-regulated by Transferrin (0.01 MB PDF) [file pone.0005198.s015.pdf]

Table S13: Genes up-regulated by Transferrin

Fold Ratio Tf/Hb	Fold Ratio Tf/Lf	Fold Ratio (Fe+/Fe-)	NMB Synonym	Gene	Gene Annotation	TIGR family
2.2	2.1	1.4	NMB1036	leuC	3-isopropylmalate dehydratase, large subunit	Amino acid biosynthesis, Pyruvate family
1.6	1.5	0.9	NMB0820		Hypothetical protein	Hypothetical proteins
1.8	1.5	1.3	unannotated between NMB1379/80		Hypothetical protein	Hypothetical proteins
