# Supplementary material for: Host Iron Binding Proteins Acting as Niche Indicators for Neisseria meningitidis
Source: PLoS One. 2009 Apr 8;4(4):e5198. doi: 10.1371/journal.pone.0005198 (PMC2662411; doi:10.1371/journal.pone.0005198)
Supplement: Table S14 — Genes down-regulated by Transferrin (0.01 MB PDF) [file pone.0005198.s016.pdf]

Table S14: Genes down-regulated by Transferrin

Fold Ratio Lf/Tf	Fold Ratio Hb/Tf	Fold Ratio (Fe-/Fe+)	NMB Synonym	Gene	Gene Annotation	TIGR family
1.9	2.8	1.2	NMB0634	fbpA	Iron(III) ABC transporter, periplasmic binding protein	Transport and binding proteins, Cations and iron carrying compounds
