# Supplementary material for: Host Iron Binding Proteins Acting as Niche Indicators for Neisseria meningitidis
Source: PLoS One. 2009 Apr 8;4(4):e5198. doi: 10.1371/journal.pone.0005198 (PMC2662411; doi:10.1371/journal.pone.0005198)
Supplement: Table S15 — Genes up-regulated by Lactoferrin (0.01 MB PDF) [file pone.0005198.s017.pdf]

Table S15: Genes up-regulated by Lactoferrin

Fold Ratio Lf/Tf	Fold Ratio Lf/Hb	Fold Ratio (Fe+/Fe-)	NMB Synonym	Gene	Gene Annotation	TIGR Family
1.5	2.3	0.8	NMB0740	recN	DNA repair protein RecN	DNA metabolism, DNA replication, recombination, and repair
1.8	4.6	0.9	NMB0546		Alcohol dehydrogenase, propanol preferring	Energy metabolism, Fermentation
1.8	1.8	1.2	NMB0907		Hypothetical protein	Hypothetical proteins
1.7	2.1	1.4	NMB2039	porB	Major outer membrane protein PIB	Transport and binding proteins, Porins
