# Supplementary material for: Host Iron Binding Proteins Acting as Niche Indicators for Neisseria meningitidis
Source: PLoS One. 2009 Apr 8;4(4):e5198. doi: 10.1371/journal.pone.0005198 (PMC2662411; doi:10.1371/journal.pone.0005198)
Supplement: Table S16 — Genes up-regulated by Lactoferrin (0.01 MB PDF) [file pone.0005198.s018.pdf]

Table S16: Genes up-regulated by Lactoferrin

Fold Ratio Tf/Lf	Fold Ratio Hb/Lf	Fold Ratio (Fe+/Fe-)	NMB Synonym	Gene	Gene Annotation	TIGR Family
1.7	2	0.5	NMB0981	serB	Phosphoserine phosphatase	Amino acid biosynthesis, Serine family
2.1	1.6	1	NMB0730		Hypothetical protein	Hypothetical proteins
1.8	1.5	0.7	NMB0755		Hypothetical protein	Hypothetical proteins
1.5	2.2	0.5	NMB1059		Conserved hypothetical protein	Hypothetical proteins, Conserved
4.3	4.6	1.7	NMB1475		Conserved hypothetical protein	Hypothetical proteins, Conserved
1.6	1.7	0.6	NMB0700	iga	IgA-specific serine endopeptidase	Protein fate, Degradation of proteins, peptides, and glycopeptides
1.5	1.6	0.7	NMB0131	rplL	50S ribosomal protein L7-L12	Protein synthesis, Ribosomal proteins: synthesis and modification
1.7	1.9	0.7	NMB0146	rpsS	30S ribosomal protein S19	Protein synthesis, Ribosomal proteins: synthesis and modification
5	4.9	1.1	NMB0941	rpmJ	50S ribosomal protein L36	Protein synthesis, Ribosomal proteins: synthesis and modification
4.8	5.5	1.1	NMB0942	rpmE	50S ribosomal protein L31	Protein synthesis, Ribosomal proteins: synthesis and modification
1.6	1.5	0.7	NMB1497		TonB-dependent receptor	Transport and binding proteins, Cations and iron carrying compounds
